# Four novel candidate causal variants for deficient homozygous haplotypes in Holstein cattle

**DOI:** 10.1038/s41598-022-09403-6

**Published:** 2022-03-31

**Authors:** Irene M. Häfliger, Mirjam Spengeler, Franz R. Seefried, Cord Drögemüller

**Affiliations:** 1 Institute of Genetics, Vetsuisse Faculty, University of Bern, Bremgartenstrasse 109a, 3001 Bern, Switzerland; 2 Qualitas AG, 6300 Zug, Switzerland

**Keywords:** Agricultural genetics, Animal breeding, Genetic association study, Genetic linkage study, Haplotypes, Sequencing

## Abstract

Mendelian variants can determine both insemination success and neonatal survival and thus influence fertility and rearing success of cattle. We present 24 deficient homozygous haplotype regions in the Holstein population of Switzerland and provide an overview of the previously identified haplotypes in the global Holstein breed. This study encompasses massive genotyping, whole-genome sequencing (WGS) and phenotype association analyses. We performed haplotype screenings on almost 53 thousand genotyped animals including 114 k SNP data with two different approaches. We revealed significant haplotype associations to several survival, birth and fertility traits. Within haplotype regions, we mined WGS data of hundreds of bovine genomes for candidate causal variants, which were subsequently evaluated by using a custom genotyping array in several thousand breeding animals. With this approach, we confirmed the known deleterious *SMC2*:p.Phe1135Ser missense variant associated with Holstein haplotype (HH) 3. For two previously reported deficient homozygous haplotypes that show negative associations to female fertility traits, we propose candidate causative loss-of-function variants: the HH13-related *KIR2DS1:*p.Gln159* nonsense variant and the HH21-related *NOTCH3:*p.Cys44del deletion. In addition, we propose the *RIOX1:*p.Ala133_Glu142del deletion as well as the *PCDH15:*p.Leu867Val missense variant to explain the unexpected low number of homozygous haplotype carriers for HH25 and HH35, respectively. In conclusion, we demonstrate that with mining massive SNP data in combination with WGS data, we can map several haplotype regions and unravel novel recessive protein-changing variants segregating at frequencies of 1 to 5%. Our findings both confirm previously identified loci and expand the spectrum of undesired alleles impairing reproduction success in Holstein cattle, the world's most important dairy breed.

## Introduction

Holstein is by far the most popular breed of cattle in the world, bred and raised for its high milk yield^1^. Especially with the improved reproductive technologies that arose in the last century, the genomic exchange over the world increased and led to the use of a limited number of prominent sires across the globe^1–3^. For Holstein cattle the difference between the absolute population size and the effective population size differs highly in all parts of the world and the inbreeding is increasing after the recent implementation of genomic selection^4^. With the effective population size being much smaller, the decrease in genetic variation and the increase in inbreeding is accelerated^2^. There has been a recognisable decline in reproductive performance in high producing dairy cattle^5,6^, which could be shown to be due to artificial selection on production traits and negative hitchhiking effects^3^. Since fertility problems are one of the most common reasons for culling cattle^7,8^ and animals of the Holstein breed are more likely to be culled than animals of other breeds^8^, there is a high need to improve female fertility, especially in Holstein cattle.

Genetic analyses of female fertility range from genome-wide association studies (GWAS) identifying genomic regions associated with fertility traits^9–12^, to the identification of genomic regions showing reduced homozygosity due to recessive mostly embryonic lethal variants^13^ and to the investigation of effects of specific variants in functional candidate genes^14,15^. Due to the routinely used single nucleotide polymorphism (SNP) array genotyping implemented in breeding programs about a decade ago, comprehensive population-wide genomic data of the current breeding populations are available. VanRaden et al*.* were the first to propose to screen this kind of data to identify genomic regions with fewer homozygous animals than expect. In their article they listed five haplotypes deviating significantly form the Hardy–Weinberg equilibrium (HWE) and thereby potentially harbouring lethal variants in the American Holstein population (Table S1)^13^. Three of these haplotypes, namely Holstein haplotypes 1, 2 and 3 (HH1-HH3), showed significant negative associations to reproduction traits. Interestingly, two subsequently performed similar analyses in the French Holstein population revealed 14 additional haplotype regions while confirming the three previously described haplotypes HH1, 2 and 3 (Table S1)^16,17^. Therefore, performing new analyses regularly as genotyping data are accumulating was recommended^17^. In two similar screenings of the Nordic Holstein population another nineteen haplotype regions in addition to the previously reported HH3 region and the *FANCI*-related brachyspina-associated haplotype HH0, originally designated as HBY, were identified by Sahana et al*.* and Wu et al*.* (Table S1)^18,19^. In the Danish Holstein population a haplotype associated with a large genomic deletion representing a loss-of-function of the *FANCI* gene causing brachyspina^20^, as well as a haplotype associated with a missense variant in the *SLC35A3* gene causing complex vertebral malformation^21^ were identified (Table 1, Table S1). The latter two variants were initially thought to cause congenital malformation, but it was shown that in homozygous state, both mutations typically cause death in utero. Therefore, the two associated haplotypes (HH0 and HHC) were included in the list of 43 known Holstein haplotypes with reduced homozygosity (Table S1).

**Table 1:** Previously identified lethal haplotype regions and their associated candidate causal variants in Holstein cattle.

Haplotype name	Associated disorder	Gene	OMIA	Variant description						Publications
				chr	Position^a^	Description	Transcript of interest^b^	Coding DNA change	Protein change	
HH0	Brachyspina	*FANCI*	000151-9913	21	21,184,869–21,188,198	Gross deletion (frameshift)		3.3 kb deletion		^19,20,27,85,86^
HH1	Embryonic lethality	*APAF1*	000001-9913	5	62,810,245	SNV (nonsense)	NM_001191507.1	c.1702C > T	p.Gln568*	^13,16,22,85^
HH2	Embryonic lethality	*IFT80*	001823-9913	1	107,172,616	SNV (frameshift)	XM_024984168.1	c.747delT	p.Leu250fs	^13,24,28,85^
HH3	Abortion	*SMC2*	001,824-9913	8	93,753,358	SNV (missense)	XM_015472668.2	c.3404T > C	p.Phe1135Ser	^13,18,19,85,87^
HH4	Abortion	*GART*	001826–9913	1	1,997,582	SNV (missense)	NM_001040473.2	c.869A > C	p.Asn290Thr	^16,27,85^
HH5	Abortion	*TFB1M*	001941-9913	9	93,223,651–93,370,998	Gross deletion		139 kb deletion		^26,27,85^
HH6	Embryonic lethality	*SDE2*	002149-9913	16	29,020,700	SNV (start-lost)	NM_001099065.2	c.2T > C	p.Met1?	^27^
HH7	Embryonic lethality	*CENPU*	001830-9913	27	15,123,636	5 bp deletion (splice site)	XM_002698654.5	c.15123637_15123640delTTACT		^17^
HHB	Bovine leukocyte adhesion deficiency (BLAD)	*ITGB2*	000595-9913	1	144,770,078	SNV (missense)	NM_175781.1	c.383A > G	p.Asp128Gly	^51,85^
HHC	Complex vertebral malformation (CVM)	*SLC35A3*	001340-9913	3	43,261,945	SNV (missense)	NM_001105386.1	c.538G > T	p.Val180Phe	^21,85,88^
HHD	Deficiency of uridine monophosphate synthase (DUMPS)	*UMPS*	000262-9913	1	69,151,931	SNV (nonsense)	NM_177508.1	c.1213C > T	p.Arg405*	^85,89,90^
CDH	Cholesterol deficiency; juvenile mortality	*APOB*	001965-9913	11	77,891,733	Large insertion (frameshift)		ERV insertion		^26,29,30,53,85^

^a^According to the reference sequence ARS-UCD1.2^31^.

^b^According to the NCBI Annotation Release 106^91^.

So far, candidate causal variants for seven of these Holstein haplotypes were identified either by whole-genome or whole-exome sequencing affecting different genes of importance for reproduction and/or development: *APAF1* (HH1), *IFT80* (HH2), *SMC2* (HH3), *GART* (HH4), *TFB1M* (HH5), *SDE2* (HH6) and *CENPU* (HH7) (Table 1)^16,17,22–28^. In addition, a haplotype associated with increased juvenile mortality due to cholesterol deficiency (CDH) in the German Holstein population^29^ had been detected to be associated with a transposable element insertion in in the *APOB* gene that causes the metabolic disorder (Table 1, Table S1)^26,29,30^.

Taken together, these findings indicate, that even though Holstein is an international breed characterized by a limited number of founding animals, the national subpopulations carry a different genetic load due to local selection decisions. Therefore, the aim of this study was to explore the accumulated massive genomic and phenotypic data of the local Swiss Holstein cattle population to identify genomic regions impairing fertility and rearing success. We applied a combination of methods including screening for missing homozygosity based on SNP-genotyped trios, trait association studies using estimated breeding values for female fertility traits and linkage disequilibrium analyses exploiting both SNP and whole-genome sequencing (WGS) data. The ultimate goal was to pinpoint potentially causative protein-coding variants explaining reproductive failure due to recessive Mendelian disorders.

## Materials and methods

The SNP array data of the Holstein population used was provided by the breeding association’s swissherdbook and Holstein Switzerland. The quality-controlled (MAF > 0.01 and call rates > 0.9 per SNP and > 0.8 per animal) SNP data included 114 890 SNP combining a variety of SNP arrays with densities ranging from 3 to 150 k. SNP positions have been updated to the latest cattle reference sequence ARS-UCD1.2^31,32^. The software Fimpute v2.2^33^ was used to impute the data in order to correct for false genotypes as well as to increase the SNP data of lower density genotyped animals. The comprehensive data included 52,961 Holstein cattle. From these data, two analysis data sets were formed: the trio-based analysis, where the complete trio was genotyped (sire, dam and offspring), with 17,915 animals and the pgp-based analysis, where the dam is replaced by the maternal grandsire (sire, maternal grandsire and offspring), with 30,315 animals. While the trio-based dataset allows tracking the direct inheritance of alleles, the pgp-based dataset includes some uncertainty. Nevertheless, the pgp-based approach, where all male animals are genotyped, represents more accurately the common genotyping scheme of current breeding programs, as can be seen from the higher number of animals in the dataset. Both datasets include animals born in Switzerland after 2009 and their ancestors’ genotypes.

The described data sets (trio and pgp) were used in the software snp1101^34^ to identify haplotypes with a significant deviation from HWE, including a correction of false discovery rate according to Benjamini-Yekutieli^35^. The chosen window size for the sliding-window approach was 50 SNP, based on the work of Hoff et al*.*^36^. The snp1101 output provides a list with all significant haplotypes, the number of observed and expected homozygous carriers, as well as the allele frequency and the haplotype itself. For all regions of interest, where the observed number of homozygous animals were lower than the number of expected homozygous animals, we have chosen the most significant haplotype (lowest p-value) to reduce the number of false positive carriers. Further, we predicted the diplotypes for these haplotypes in the entire genotyped Holstein population and selected carrier animals for WGS.

Association studies were conducted using the predicted diplotypes and all female reproduction traits from the Swiss routine genetic evaluation system. In total 18 traits were analysed, while traits can be grouped into fertility, birth and survival traits (Table 2). The estimated breeding values (EBV) were deregressed and used as pseudo-phenotypes in linear mixed models (LMM) in the GCTA software^37,38^. The genomic relationship matrix estimated using the method GRM of the GCTA software was included in the model to account for population structure. In addition, for the SNP data described above genome-wide association studies (GWAS) together with deregressed EBVs as pseudo-phenotypes were performed. These single SNP regressions were implemented using the software snp1101^34^, the genomic relationship matrix was calculated with the method proposed by VanRaden^39^ and a Bonferroni-correction was applied (p < 0.0000044).

**Table 2:** Traits of interest.

Trait group	Trait sub-group	Trait	Description	Abbreviation
Fertility traits	Fertility	Non-return rate heifer	Heifers non-return rate after 56 days, binary	NRh
		Non-return rate cow	Cows non-return rate after 56 days, binary	NRc
		Interval first to last insemination heifer	Interval between first and last insemination for heifer, days	IFLh
		Interval first to last insemination cow	Interval between first and last insemination for cows, days	IFLc
		Interval calving to insemination	Interval from calving to first service, days	DFS
Birth traits	Birth history direct	Percentage normal births	Calving ease, scored between 1-without help to 5-dystocia	CEd
		Percentage live births	Percentage of calves born alive	SBd
		Birth weight	Weight of calve at birth, kg	BWd
		Gestation length	Days from successfull insemination to birth	GLd
		Multiple birth	Percentage of multiple births	MBd
	Birth history maternal	Percentage normal births	Calving ease, scored between 1-without help to 5-dystocia	CEm
		Percentage live births	Percentage of calves born alive	SBm
		Birth weight	Weight of calve at birth, kg	BWm
		Gestation length	Days from successfull insemination to birth	GLm
		Multiple birth	Percentage of multiple births	MBm
Survival traits	Rearing success	Survival period 1	Survival from day 3 up to 30th day of life	P1
		Survival heifer period 2	Survival of heifers from day 31 up to 458 days	P2h
		Survival bull period 2	Survival of young bulls from 31 up to 183 days	P2b

Whole-genome sequencing (WGS) was performed in an attempt to sequence animals potentially carrying variants causing reduced homozygosity in the identified genomic regions. Therefore, 37 Holstein animals were selected based on their diplotype status for WGS. Additionally, 656 genomes from other projects were available, leading to 691 publicly available genomes including 244 purebred Holstein cattle (Table S2). Sequence data preparations was done according to previously described methods^40^, with the only difference in the recalibration step, where the known variants from the 1000 Bull Genomes Project run 7 (BQSR file version 2) were used^41^. This resulted in a variant call format (VCF) file encompassing all single-nucleotide variants (SNV) and short indels from the 691 animals. The average read depth per genome were calculated using covstat from goleft v0.1.19^42^ and varied from 3.3× to 72.8×, with an average of 18.7× read depth (Table S1).

In order to design a custom SNP array, we selected candidate SNVs and short indels within the genomic regions of interest defined by the determined haplotype regions including plus/minus 2 Mb flanking sequence up- and downstream. Due to the variation in read depth, missing values and a single homozygous carrier animal were allowed, GATK recommended quality measures for hard filtering needed to be passed and at least a single Holstein animal needed to be carrier of a variant, but no more than 75% of all animals for it to be selected for the array design. Variants were included in a custom array using Axiom Microarray Genotyping Technology, designed under the umbrella of Swiss routine genomic systems. Due to the process of the design almost every second (44%) of the initially selected 465,768 variants could be designed and led to an array including 205,362 novel variants and 112,854 so called “routine variants” from previously applied arrays including common SNP markers considered for genomic selection. Subsequently, this custom array, called SWISScow array, was used routinely in the Swiss genomic breeding program. Therefore, comprehensive genotypes of 13,667 Swiss cattle are available, of which 5603 are Holstein.

Linkage disequilibrium (LD) (r^2^) were estimated between diplotypes for haplotypes and both, custom array genotypes (a) and WGS-driven genotypes (b), in a merged dataset encompassing 5 603 (a) and 37 (b) animals, respectively. These analyses were conducted using plink v1.9^43^.

**Figure 1:**
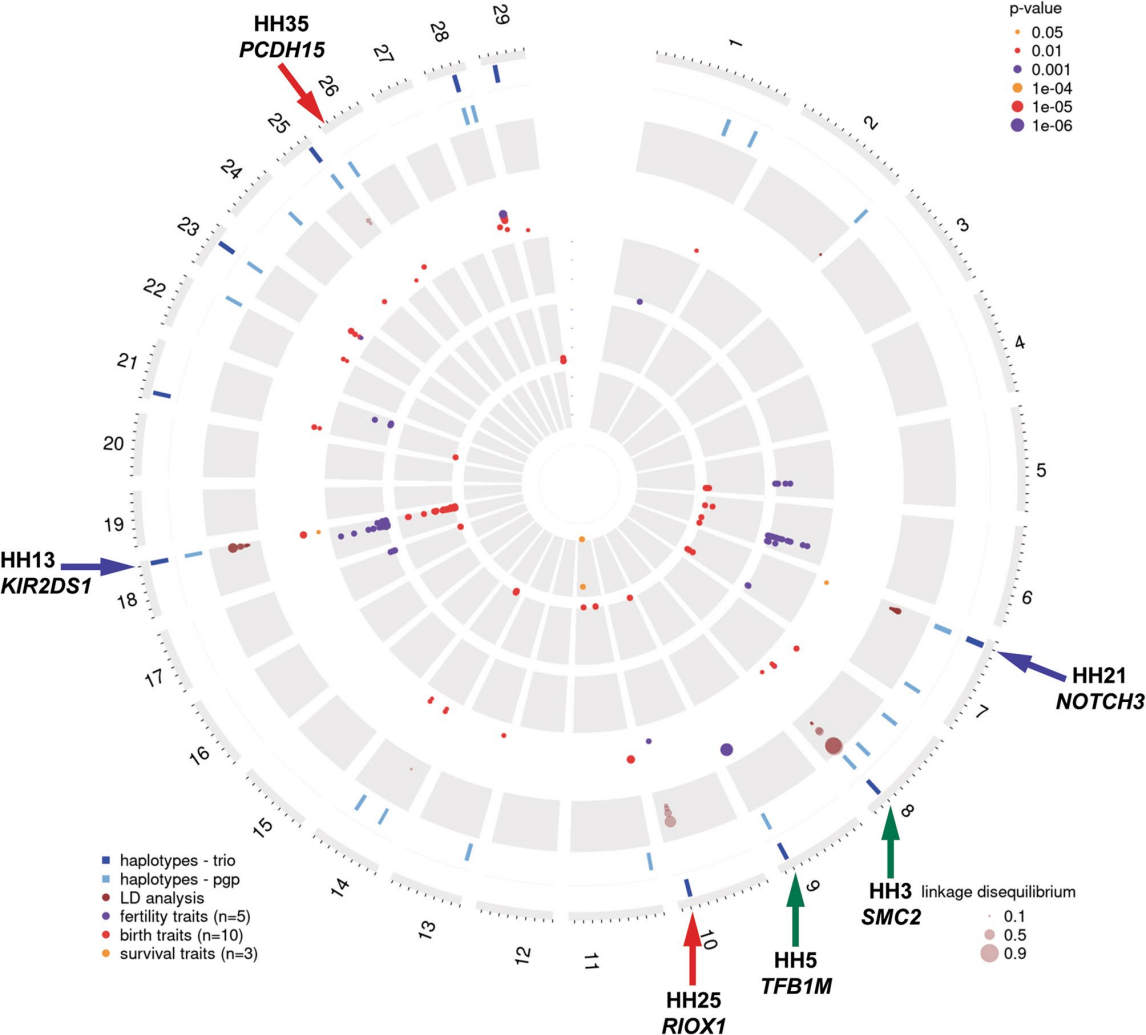
Summary of the SNP and WGS data analyses of the Swiss Holstein population. This plot visualises all the comprehensive genomic analysis. The most outer circles show the haplotypes per chromosome with reduced homozygosity for the trio approach in dark blue and the pgp approach in light blue. LD (r^2^) between haplotypes and markers of the custom SNP array is indicated in the circle with the brown dots. Note the dot size correlates with the LD extent. The fourth circle shows significant results from the haplotype to phenotype association analyses. Note that the three evaluated trait groups are represented by different colours and the dot size correlates with the significance values. The three inner circles present the significant (p < 0.0000043) GWAS results across the fertility (purple), birth (red) and survival (yellow) traits. Scales are based on the − log10(p-value). Note the green arrows indicating previously identified haplotypes and candidate causal variants. The blue arrows indicate the previously identified haplotypes HH21 and HH13^13,16,18^ and the herein described candidate causative variants in the genes *NOTCH3* and *KIR2DS1,* respectively. Finally, the red arrows indicating the newly identified haplotypes HH25 and HH35 harbouring most likely causative variants in the genes *RIOX1* and *PCDH15*.

For visualisation of the results from the different genome-wide analyses the R package OmicCircos was used^44^. Figure 1 summarizes the results from the haplotype identification using the trio and pgp approach, indicates if there are linked marker on the custom array, if the haplotypes show associations to current traits of the breeding program, and if there are significant GWAS associations in the Swiss Holstein population for the trait groups fertility, birth and survival.

In order to improve the interpretation of candidate variants conservation scores PhyloP and PhastCons from the UCSC database were taken into account^45,46^. Therefore, the tool LiftOver from UCSC tools was used to lift the bovine variants from the reference sequence ARS-UCD1.2^31^ to the human genome 38^47^. For these positions, the conservation scores of 99 vertebrates could be extracted directly. Furthermore, for a more comprehensive prediction of the impact of protein-changing variants the tool PROVEAN^48^ was applied. Lastly, the data provided by the 1000 Bull Genomes project run 8 was analysed for the distribution of candidate variants, as it is an international control cohort with a variety of breeds^41^.

## Results

### Detection of novel and previously described haplotypes

All identified haplotypes are described in Table 3, their statistical evidence is summarized in Table S3 and the two outer circles in Fig. 1 demonstrate their distribution across the 29 bovine autosomes. The search for haplotypes deviating from HWE led to the detection of 24 haplotype regions, of which five had been previously described (Table 3, Table S3). These identified haplotypes were named in accordance to 17 previously described Holstein haplotypes (Table S1) and designated as subsequent haplotypes (HH18–HH38) (Table 3, Table S3). The obtained results from the trio-based approach led to the detection of 10 haplotypes, of which 6 were never observed in homozygous state, although at least 10 were expected (Table 3). On the other hand, the pgp-based approach revealed 21 haplotypes, of which 4 never occurred in homozygous state. Combining the outcome of both approaches revealed 7 commonly detected haplotype regions. The most significant deficient homozygous haplotype is the previously described haplotype HH21 for which 157 homozygous carriers were expected, but none were observed (Table 3, Table S3)^18^. The identified haplotypes have an average length of 1.12 Mb and range from 0.24 up to 2.56 Mb and segregate at an average allele frequency of 0.024 ranging from 0.014 to 5.46 (Table S3).

**Table 3:** Haplotypes indicating reduced homozygosity in Swiss Holstein.

Name^a^	chr	Pos in Mb^b^	Analysis	Homozygous haplotype carrier			Allele frequency %	Known and novel associated gene^c^	Associated traits		
				observed	expected	deficient			Fertility	Birth	Survival
HH18	1	10.302–104.376	pgp	7	28	75%	2.39				
HH19^d^	1	139.874–140.643	pgp	12	47	74%	3.00		GLd		
HH20	2	134.477–135.148	pgp	1	14	93%	1.66				
HH21^*e*^	7	7.872–10.433	trio/pgp	0	157	100%	5.46	*NOTCH3*			P2b
HH22	7	93.582–94.642	pgp	2	16	88%	1.73				
HH23	8	15.562–16.863	pgp	5	25	80%	2.20			MBm	
HH24	8	66.699–68.006	pgp	2	59	97%	3.36		NRh	BWm, CEm, MBm	
HH3^f^	8	90.959–92.085	trio/pgp	0	10	100%	1.38	*SMC2*	GLm		
HH5^*g*^	9	90.928–91.824	trio/pgp	0	30	100%	2.39	*TFB1M*	NRh		
HH25	10	86.876–87.773	trio	5	20	75%	1.96	*RIOX1*	DFS		
HH26	11	4.531–5.362	pgp	7	34	79%	2.56		GLd		
HH27	13	7.083–8.297	pgp	2	17	88%	1.82		GLm		
HH28	14	24.591–24.827	pgp	3	20	85%	1.98			SBd, SBm	
HH29	14	58.128–59.238	pgp	14	52	73%	3.14			SBm, CEm	
HH13^h^	18	60.932–62.101	trio	1	17	94%	1.81	*KIR2DS1*		BWd	P1
		62.070–63.045	pgp	2	22	91%	2.06				
HH30	21	7.844–8.671	trio	1	24	96%	2.13			BWd, CEd, CEm	
HH31	22	59.811–60.704	pgp	3	17	82%	1.80		GLd, GLm		
HH32	23	32.773–33.726	pgp	1	13	92%	1.57			BWd, CEm	
		33.545–34.948	trio	0	13	100%	1.55		GLd, IFLc	CEm	
HH33	24	44.693–45.678	pgp	14	42	67%	2.82			SBd	
HH34	25	36.162–37.330	trio /pgp	0	18	100%	1.86		GLm		
HH35	26	3.359–4.235	pgp	7	31	77%	2.44	*PCDH15*		SBm	
HH36	28	28.541–29.736	pgp	2	15	87%	1.71			CEm	
		29.731–30.877	trio	2	26	92%	2.25				
HH37	28	40.348–41.271	pgp	6	66	91%	3.54		GLd, GLm, IFLh	CEm, SBd, MBm	
HH38	29	14.243–15.317	trio	0	14	1.00	1.66			CEm	

*BWd* weight of calve at birth in kg, direct trait, *BWm* weight of calve at birth in kg, maternal trait, *CEd* direct calving ease, scored between 1-without help to 5-dystocia, *CEm* maternal calving ease, scored between 1-without help to 5-dystocia, *DFS* interval from calving to first service in days, *GLd* days from successful insemination to birth, direct trait, *GLm* days from successful insemination to birth, maternal trait, *IFLc* interval between first and last insemination for cows in days, *IFLh* interval between first and last insemination for heifer in days, *MBm* percentage of multiple births, *NRh* heifers non-return rate after 56 days, binary, *P1* survival from day 3 up to 30th day of life, *P2b* survival of young bulls from 31 up to 183 days, *SBd* percentage of calves born alive, direct trait, *SBm* percentage of calves born alive, maternal trait.

^a^HH meaning Holstein haplotype.

^b^According to the reference sequence ARS-UCD1.2^31^.

^c^According to NCBI Annotation Release 106^91^.

^d^Haplotype co-localises with previously described haplotype HHB by Shuster et al*.* and Cole et al*.*^51,85^.

^e^Haplotype described before as 175.5 and 07–126 by VanRaden et al. and Sahana et al.^13,18^.

^f^Haplotype previously described by VanRaden et al., McClure et al., Sahana et al. and Wu et al.^13,18,19,87^.

^g^Haplotype previously described by Schütz et al. and Fritz et al.^26,27^.

^h^Haplotype previously described by Fritz et al.^16^.

### Association studies indicate phenotypic relevance of deficient homozygous haplotypes

Significant associations (p < 0.05) were detected for 21 haplotypes distributed across 17 chromosomes (Fig. 1, Table 3). Nevertheless, three of the detected deficient homozygous haplotypes show no significant associations to any of the 18 considered traits (Table S4). Most of the deficient homozygous haplotypes are associated with various fertility- and birth-related traits, whereas only two detected haplotypes show significant association to two traits of calf survival (Table 3). For example, HH37 shows significant associations to totally six different analysed fertility- and birth-related traits (Table 3). Detailed results from the association studies including all 24 detected deficient homozygous haplotypes and 18 traits of interest are summarised in Table S4. The by far strongest association was detected for the HH5 haplotype to the trait NRh (heifers non-return rate after 56 days) (p = 0.00006), which is in accordance with previously reported findings that a gross genomic deletion on chromosome 9 encompassing the entire *TFB1M* gene causes embryonic lethality^26^. Another example of a deficient homozygous haplotype showing a negative association to non-return rate in heifers is HH24 (p = 0.02), beside further significant associations of that haplotype to three birth-related traits (Table 3). The fertility-related trait interval DFS (calving to first insemination) is negatively associated with the HH25 haplotype and the trait IFLc (interval fist to last insemination) with the HH32 haplotype whereas HH32 and HH30 show a reducing effect on the trait BWm (birth weight) (Table S4). The herein described deficient homozygous haplotype HH21 most likely corresponds to the previously described haplotypes 175.5 (VanRaden et al*.*^13^) and 07-126^18^ and shows a negative association to the survival-related traits P2b (survival from day 3 up to 30 days) (Table 3). The also previously published deficient homozygous haplotype HH3 shows significant association to the birth-related trait GLm (gestation length) (Table 3), which can be explained with the already known *SMC2*-related abortion of homozygotes^24^. Finally, for the further two haplotypes with putative known causal variants (see below) HH35 shows a negative effect for the birth-related trait SBm (live births) while HH13 is associated to BWm (birth weight) as well as the survival related trait P1 (survival from day 3 up to 30th day of life) (Table 3).

In addition, the GWAS results are visualised using Manhattan plots in the supplementary material (Supplementary Figs. S1–S18). The genome-wide significant (p < 0.0000043) results are collected in Table S5 and in summary displayed in the three inner circles of Fig. 1. Among the five studied fertility-related traits we identified clear associations to a locus on chromosome 18 for the three traits DFS (calving to insemination), NRh (non-return rate for heifer) and IFLc (interval between first and last insemination for cows), that co-localize with the region of the herein detected deficient homozygous HH13 (Table S5). In addition, at the same locus interesting associations to five different birth-related traits were observed (Table S5). At the genome region of HH28 on chromosome 14, a GWAS hit to the two birth-related traits direct traits BWd (birth weight) and CEd (percentage normal births) was observed (Table S5). No co-localization between GWAS signals of the three studied survival traits and the herein detected deficient homozygous haplotypes could be identified. Nonetheless, a GWAS hit on chromosome 11 for the calf survival-related trait P2h (survival of heifers from 31 up to 458 days) was observed at the identical genome region on chromosome 11 of the previously described CDH haplotype containing a deleterious *APOB* loss-of-function variant causing rearing loss (Table S1)^29^.

### Identification of four novel potentially causal variants

The generated comprehensive WGS data was mined in genome regions containing deficient homozygous haplotype for variants potentially causing pre- or post-natal lethality or sublethal conditions. By filtering for variants indicating an obvious depletion in homozygosity and showing a strong LD with the haplotype, we successfully uncovered five protein-changing DNA variants as candidates (Table 4). This short list of possibly causal variants includes the previously described missense mutation in the *SMC2* gene associated with HH3-related embryonic lethality^24^ (Table S6). In addition, we detected four novel variants linked to different deficient homozygous haplotypes affecting these four genes: *NOTCH3* (HH21), *RIOX1* (HH25), *KIR2DS1* (HH13), and *PCDH15* (HH35) (Table 4). Unfortunately, only two of these five variants, those affecting *SMC2* and *KIR2DS1*, could be successfully added to the customized SWISScow array and thereby genotyped in several thousand animals of the current Swiss dairy population. In accordance to the results of the haplotype analysis for HH3 and HH13, no single homozygous carrier of the *SMC2* and *KIR2DS1* variants was observed neither in the current population of more than 14 thousand Swiss dairy cattle nor in any other breed of cattle included in the 1000 Bull Genomes project (Table S6).

**Table 4:** Candidate variants potentially explaining the identified haplotype regions.

Haplotype	Gene	OMIM	Variant description				
			Genomic position^a^	Description	Transcript^b^	Coding DNA change	Protein change
HH21^c^	*NOTCH3*	600276	chr7:7913459	3 bp deletion (inframe deletion)	XM_003586246.3	c.129_131delTTG	p.Cys44del
HH3^d^	*SMC2*^e^	605576	chr8:93753358	SNV (missense)	XM_015472668.2	c.3404T > C	p.Phe1135Ser
HH25	*RIOX1*	611919	chr10:84938370	30 bp deletion (inframe deletion)	NM_001099702.1	c.396_425delGGCGCAGACCCCGGCGGCACGCTTGGTGGA	p.Ala133_Glu142del
HH13^f^	*KIR2DS1*	604952	chr18:62758881	SNV (nonsense)	NM_001097567.1	c.475C > T	p.Gln159*
HH35	*PCDH15*	605514	chr26:5325675	SNV (missense)	XM_015460562.2	c.2599C > G	p.Leu867Val

^a^According to the reference sequence ARS-UCD1.2^31^.

^b^According to NCBI Annotation Release 106^91^.

^c^Haplotype described before as 175.5 and 07-126 by VanRaden et al*.* and Sahana et al*.*, respectively^13,18^.

^d^Haplotype previously described by VanRaden et al*.*, McClure et al*.*, Sahana et al*.* and Wu et al*.*^13,18,19,87^.

^e^Variant previously detected by McClure et al*.*^24^.

^f^Haplotype previously described by Fritz et al*.*^16^.

As reported earlier by Ref.^24^ the *SMC2* missense variant p.Phe1135Ser affects an evolutionary conserved residue of the C-terminal P-loop-nucleoside triphosphate hydrolase domain classified as a deleterious change according to in silico predictions. In the studied Swiss Holstein population this variant occurs a low frequency (1.3%) in high LD (r^2^ = 0.85) with the HH3 haplotype on chromosome 8 at ~ 94 Mb (Table S6). The *SMC2* variant was absent from all other studied breeds (Table S6).

**Figure 2:**
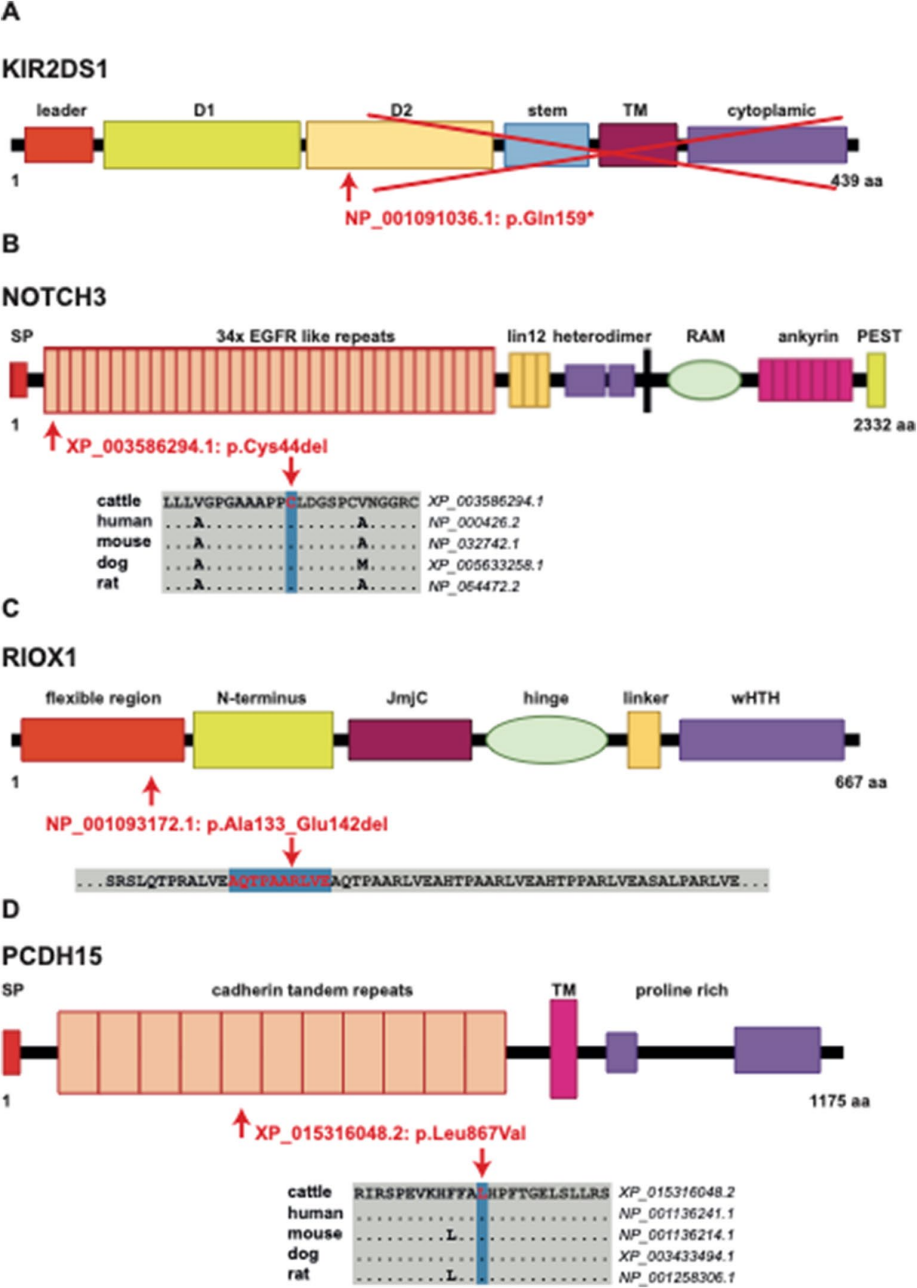
Features of the four novel candidate causal variants. Note the predicted protein changes of the detected variants indicated in red. ((**A**) *KIR2DS1:*p.Gln159*^82^, (**B**) *NOTCH3:*p.Cys44del^76^, (**C**) *RIOX1:*p.Ala133_Glu142del^83^, (**D**) *PCDH15:*p.Leu867Val^84^).

The detected stop-gain variant in the *killer cell immunoglobulin like receptor, two Ig domains and short cytoplasmic tail 1* (*KIR2DS1*) gene showing missing homozygosity is located on chromosome 18 at ~ 62 Mb in LD (r^2^ = 0.49) with the HH13 haplotype. The detected *KIR2DS1* variant located in exon 4 leads to the premature termination of the protein sequence at codon 159 (p.Gln159*) truncating the protein by almost two third including functionally important domains (Fig. 2A). It segregates within the Swiss Holstein population at an allele frequency of 5% and shows a highly significant deviation from HWE (p = 0.0000007) based on a Chi-square test (Table S6). Interestingly the *KIR2DS1* variant obviously occurs in various other breeds beside Holstein cattle (Table S6).

Further, we propose three coding variants in *NOTCH3*, *RIOX1,* and *PCDH15* as candidate causal variants that were so far not evaluated in the broader population due to limitations in the design process of the customized SWISScow array.

Firstly, we identified a disruptive in-frame deletion in the *notch receptor 3* (*NOTCH3*) gene moderately linked (r^2^ = 0.49) with the completely deficient homozygous haplotype HH21 on chromosome 7 at ~ 9 Mb (Table 4). This protein-changing variant knocks-out a highly conserved cysteine residue (p.Cys44del) of an epidermal growth factor-like repeats (EGFRs) that comprise the extracellular domain of the NOTCH3 receptor. The deletion results in a loss of a cysteine residue in the first of these numerous EGFRs (Fig. 2B) and is predicted to be deleterious (Table S6). This variant occurs at a low allele frequency of 0.014 and was never observed in homozygous state in any of the more than 4000 bovine genomes with available WGS data (Table S6). The studied *NOTCH3* variant appears to be very rare, and among the 4109 animals of the 1000 Bull Genomes project, it was found in only three animals of different breeds (Table S6).

Secondly, another disruptive in-frame deletion encompassing 30 nucleotides of the open reading frame of the *ribosomal oxygenase 1* (*RIOX1*) gene was detected that occurs perfectly linked (r^2^ = 1) with the HH25 haplotype on chromosome 10 at ~ 87 Mb (Table S6). Interestingly, within our complete WGS data a single homozygous animal was detected, whereas 16 homozygotes were present in the 1000 Bull Genomes variant catalogue. By visual inspection of the mapping quality of the reads around this variant, we noticed that the mapping algorithm (Supplementary Fig. S19) did not splice many of the reads covering the 30 bp deletion properly. Therefore, we assume that these homozygotes most likely represent false positives and are actually only heterozygous carriers of the *RIOX1* variant. Obviously, the *RIOX1* deletion occurs in many breeds other than Holstein (Table S6). The disruptive in-frame deletion affects the N-terminal region of the RIOX1 protein (p.Ala133_Glu142del) (Fig. 2C).

Lastly, we identified a missense variant in the *protocadherin related 15* (*PCDH15*) gene that is perfectly linked (r^2^ = 1) with the HH35 haplotype on chromosome 26 at ~ 4 Mb (Table S6). Interestingly, for this variant, as well as for the HH35 haplotype we observed some homozygous carriers (Table 3, Table S6). In the 1000 Bull Genomes Project variant catalogue all identified variant carriers were indicated as Holstein, Ayrshire and Norwegian Red (Table S6). Nevertheless, the amino acid exchange (p.Leu867Val) affects a highly conserved residue of a cadherin tandem repeat domain (Fig. 2D) although the computer predicted consequence was inconclusive (Table S6).

## Discussion

After giving an overview of the known haplotypes with missing homozygosity in the worldwide Holstein breed, we have conducted a comprehensive survey of genomic and phenotypic data of the Swiss Holstein cattle population to search for further deficient homozygous haplotypes. We focussed on the detection of hidden recessive variants and found five candidates that cause the observed missing homozygosity. These variants can lead to the exclusion from the population due to natural selection processes, e.g., embryonic lethality, abortions and stillbirth, or due to artificial selection against animals that show illthrift or other non-lethal more subtle conditions, e.g., metabolic disorders that are often not diagnosed.

In previous studies performed in Holstein cattle a simple allele frequency-based approach was applied to identify haplotypes showing a depletion of homozygous animals by applying the assumption of random mating and a Chi-squared test^13,18,49^. Other studies accounted for the mating status by included the haplotype state of the sire and the maternal grandsire^16,50^. In comparison, the herein presented study uses two different approaches, a trio- and pgp-approach, that include genotyped trios and applies a Fisher exact test. This had the advantage that we were able to detect haplotypes that segregate at a lower allele frequency. The pgp-based approach includes almost double the data from the trio-based approach. This is explained by the genotyping management, in which male breeding animals are usually genotyped, but by far not all females. Therefore, we identified almost twice the number of haplotypes with the pgp-approach. Nevertheless, the applied trio-approach is assumed to be more powerful as the parental haplotype inheritance can be traced directly.

Introducing this novel approach, we were able to verify our outcomes with the results of earlier studies in different Holstein cattle populations. With the applied methods, we were able to detect four haplotype regions, namely HH3, HH5, HH13 and HH21 that were previously described to occur in several subpopulations of Holstein cattle^13,16,18,26,51^. Another herein detected haplotype (HH19) co-localizes with the genome regions of the known deficient homozygous haplotype HHB on the proximal end of chromosome 1. However, it obviously differs to the BLAD-associated HHB haplotype as we confirmed by using the customized SWISScow array that the causative *ITGB2* variant does not segregate anymore in the Swiss Holstein population (results not shown) probably due to the strict exclusion of carriers in the early 1990s.

Furthermore, by applying the WGS data screen, we confirmed the known Holstein breed-specific HH3-linked missense variant in *SMC2* on chromosome 8 that also in our data showed complete missing homozygosity^24^. Interestingly, for this deleterious variant we could only detect a single significant phenotypic effect in our additive models for gestation length based on the haplotype, but a suggestive negative effect on non-return rate in heifer. Negative effects on the number of inseminations in heifers within that genomic region were shown in Canadian Holstein cattle^52^. Nevertheless, these findings support a purely recessive embryonic lethal effect of the *SMC2* variant that allows merely the indirect detection in non-return rate^24^. For HH5, we were unfortunately unable to confirm the association with the most likely causative gross deletion encompassing the *TFB1M* gene as we focused in the scope of this project on SNV and short indel genotyping.

Interestingly, we found a co-localization of a GWAS peak for survival during the first 458 days of life in the region of the previously reported CDH haplotype^29^. The CDH haplotype was shown to be linked with a pathogenic endogenous retroviral insertion into the *APOB* gene and thereby leading to a subvital malabsorption of cholesterol, provoking increased juvenile mortality^26,29,30,53^. This haplotype was difficult to detect due to it segregating in homozygous state in the adult population, as the LD between the CDH haplotype and the *APOB* variant was not perfect as the ancestral version of the haplotype still occurred^29,30^. Initially, cholesterol deficiency was thought to be a recessive or codominant inherited disorder, what led to the identification of the responsible locus by applying a case–control based GWAS^29^. Further studies examining heterozygous carriers showed that the *APOB-*associated disorder is not a simple Mendelian disease, as heterozygous animals can also show severe clinical signs of cholesterol deficiency^54^. The identified GWAS peak in the current study underlines these findings with an additive model. For a monogenic recessive lethal disorder, one would not expect to see any effect within an additive model, as all the homozygous affected animals would be more or less excluded from the genotyped population and it can be assumed that these heterozygous animals are phenotypically normal like the homozygotes. It is in line with recent findings in cattle that additive models are shown to have their limitations and non-additive models should be taken into account for further phenotypic evaluations of recessive disorders^55^.

We propose to add four novel candidate causative variants to the list of Holstein haplotypes with known cause for missing homozygosity. We emphasize to highlight two variants in *KIR2DS1* and *NOTCH3* linked to the abovementioned confirmed deficient homozygous haplotypes HH13 and HH21, respectively. Both detected variants segregate in Holstein cattle but, in contrast to the HH3-linked *SMC2* variant, represent derived alleles that predates establishing of modern breed as they rarely occur also in unrelated breeds of cattle.

Interestingly, in human *KIR2DS1* is reported to be associated with reproduction, namely placentation success^56,57^. *KIR2DS1* is an activator receptor, belongs to the KIR family of natural killer cell Ig-like receptors and plays a vital role in the immune system (OMIM #604952). Regarding the involvement in disorders, *KIR2DS1* is known to be associated in human with an autoimmune disease called psoriasis vulgaris^58,59^ and leukaemia^60–62^. In later studies reproductive failure and foetal growth restriction were associated with maternal KIR2DS1 and foetal leukocyte antigen C2 (HLA-C2) imbalance, however, being complicated by the interference of the maternal and the embryonic genotype^56,63–66^. Nonetheless, the function of KIR2DS1 was described as protective against pregnancy loss^66,67^. Moreover a study showed the importance of KIR2DS1/HLA-C2 in response to viral placental infections^68^. The herein detected bovine variant most likely represents a true loss-of-function mutation of *KIR2DS1*, as possibly nonsense-mediated decay selectively recognizes and degrades mRNAs whose open reading frame is truncated by a premature translation termination codon. Therefore, this variant is supposed to lead to pregnancy losses of homozygous embryos. Furthermore, as the function of KIR2DS1 is sensitive to its expression^68^, we speculate that also a negative effect in heterozygous carriers might exist. This hypothesis is supported by GWAS hits in the genome region of HH13 for several fertility-related traits (interval calving to insemination, non-return rate heifer and interval first and last insemination), as well as birth-related traits (percentage normal births, percentage live births, birth weight as well as gestation length for maternal and direct traits). Interestingly, major QTLs for fertility and birth traits were mapped at 60 Mb on chromosome 18 in the German Holstein population^69^.

The *NOTCH3* gene belongs to the mammalian Notch family that includes four proteins, which are transmembrane developmental signalling receptors^70–72^. The Notch signalling pathway has important roles in the development of organs, regulation of cell death, cell survival and cell abundance^70–72^. *NOTCH3* is known to be associated with fatal dominant inherited disorders such as myofibromatosis, cerebral arteriopathy with subcortical infarcts and leukoencephalopathy (CADASIL) and lateral meningocele syndrome (OMIM # 600276), as well as different forms of cancer^73^. In *Notch3*-deficient mice, it was initially shown that *Notch3* seems to be non-essential for proper embryonic development and fertility^74^, however, post-natal pathology in *Notch3*-null mice revealed structural defects in the smooth muscle cells, arterial differentiation and the arterial morphology^75^. The *NOTCH3* variant linked to the HH21 haplotype is predicted to delete a highly conserved cysteine residue in the first of 34 EGFR modules that are characterised by six highly conserved cysteine’s that are essential for the stability of each EGFR-module fold by their three disulphide bonds^76^. EGFR1–3 had been shown to be critical for Notch pathway activation^77^. Interestingly, the most significantly associated trait with HH21 was survival of young bulls from 31 up to 183 days, which indicates a possibly subvital condition caused by the *NOTCH3* variant. So far, the proposed variant was never observed in homozygous state in any of more than 4000 cattle genomes, while a population evaluation in Swiss Holstein is still pending to prove if there are indeed 100% no homozygous carriers as to be assumed from the haplotype analysis.

Lastly, we would like to propose two candidate causal variants in *RIOX1*, and *PCDH15*, that show perfect LD to the deficient homozygous haplotypes HH25 and HH35, respectively. *RIOX1* is an oxygenase that can act as both a histone lysine demethylase and a ribosomal histidine hydroxylase and plays a central role in histone code, chromatin organisation and DNA transcription regulation (OMIM #611919). Due to the basic importance of these processes for DNA and cell function, we speculate that a disruption of the protein by a motif of 10 amino acids might lead to disturbance of the meiosis or to the inactivation of cell differentiation in very early stages of the embryogenesis. This hypothesis would be supported by the haplotype association for HH25 that indicates a prolonged interval calving to first insemination (DFS). Here we also would like to address the fact that such small deletions affecting repetitive DNA motifs clearly show up the limitations of the applied short-read methods for whole-genome sequencing and subsequent indel calling (Supplementary Fig. S19). Nevertheless, because the variant *RIOX1* allele obviously occurs in a variety of breeds, this variant might represent a quite old mutation that arose already before modern breed formation that could also be of less impact. If the proposed variant is indeed reduced in number of homozygous carriers needs to be evaluated by further genotyping.

Finally, we propose a missense variant in *PCDH15* as strong causal candidate for the depletion in homozygosity of HH35. The occurrence of this bovine variant is limited to Holstein and animals of populations with documented introgression of Holstein cattle. It affects an evolutionary conserved residue of a functionally important domain and the affected gene is a member of the cadherin superfamily^78^. It encodes an integral membrane protein that is known to be vital for the maintenance of normal retinal and cochlear function (OMIM #605514). In men, the affected gene *PCDH15* is associated with recessively inherited forms of deafness and neurosensorial Usher syndrome^79^. The HH35 haplotype is not completely deficient homozygous indicating no embryonic lethal effect of the associated *PCDH15* variant. Therefore, we speculate that possibly a subtle disorder leads to the social exclusion of young individuals, probably due to their abnormal behaviour as they have a different perception of the environment than unaffected animals. Alternatively, we hypothesize that possibly not all biological functions of PCDH15 are known and more severe conditions could arise leading to pre- or neonatal lethality and thereby explaining the lack of homozygotes. Nevertheless, we suggest also for this newly described variant in *PCDH15* to follow up by extended genotyping and propose a detailed clinical examination of homozygous living animals to evaluate their individual health status.

At this point we would like to lay out some limitations of the presented study. The imputation step, which is based on pedigree information and allele frequencies, can affect the detection of decreased homozygosity. Therefore, imputation has limited power for recently arisen variants due to the existence of ancestral and derived versions of identical haplotypes. This could potentially lead to the false negative detection of recent haplotype regions and the false positive detection of haplotype regions due to naturally over-represented alleles. Furthermore, the accuracy of imputation depends on the SNP density of the genotype array and number of genotypes available per array^80^. During the design of the custom genotyping array, it was not possible to include all variants of interest. Mainly due to technical reasons, but also due to the more complicated types of variants (e.g. deletions). The manufacturer did not recommend those variants since in silico predicted genotyping quality was expected to be limited. Nevertheless, as shown for the HH3 and HH13 associated variants in *SMC2* and *KIR2DS1*, the custom array was a very efficient tool to monitor several thousands of animals. The displayed results illustrate that even though Holstein is an international breed local selection strategies affect the genomic structure of its subpopulations. From a genome-wide perspective, it might be possible that we have overlooked additional haplotype regions due to the restricted sliding-window length of 50 SNPs, considering that there is a correlation between window size and the detected homozygosity^36^. In addition, the restriction for protein-changing SNVs and indels limits the identification for candidate causal variants. We have not considered non-coding regulatory variants or larger structural variants that have a pathogenic impact, which we do not know yet. An example for this is the HH5 associated large deletion including the gene *TFB1M*^26^, for which we could confirm the haplotype region in our data. Furthermore, it was shown that graph based reference sequences can improve the annotation and could potentially lead to the detection of further protein-changing variants^81^. In order to improve the understanding of the described candidate variants, further studies are recommended to evaluate all homozygous haplotype and variant carriers. As the example of CDH in Holstein cattle has taught, the oversimplification of the depletion of homozygosity by assuming the cause to be a monogenetic recessive disorder might be misleading.

In conclusion, we demonstrate that with mining massive SNP data in combination with WGS data, we mapped 19 novel and 5 previously described haplotype regions and unravel novel recessive protein-changing variants in *NOTCH3*, *RIOX1*, *KIR2DS1* and *PCDH15* segregating at low to moderate frequencies. In addition, our findings confirm previously identified loci such as *SMC2* for HH3. The phenotypic associations support additive effects in the described haplotype regions and variants. Taken together this study expands the spectrum of undesired alleles impairing reproduction success in Holstein cattle, the world's most important dairy breed, enabling improved DNA-based selection in order to reduce reproductive failure and animal loss.

## Supplementary Information

Supplementary Information.

## Data Availability

The whole-genome sequencing data is stored in the European Nucleotide Archive and can be found with the sample IDs available in Table S2. For access to the SNP genotyping data, we ask interested people to contact the authors or the breeding associations directly.
