# Supplementary material for: Four novel candidate causal variants for deficient homozygous haplotypes in Holstein cattle
Source: Sci Rep. 2022 Mar 31;12:5435. doi: 10.1038/s41598-022-09403-6 (PMC8971413; doi:10.1038/s41598-022-09403-6)
Supplement: Supplementary file 1 — Supplementary Information. [file 41598_2022_9403_MOESM1_ESM.pdf]

Figure S1-S18: Manhattan plots and their QQ-plots of the GWAS of the Swiss Holstein population. There is a page for the fertility, birth and growth-related trait group, including a Manhattan plot for every single trait in the group trait according to Table 2.

HO fertility traits

Figure S1: GWAS trait NRh

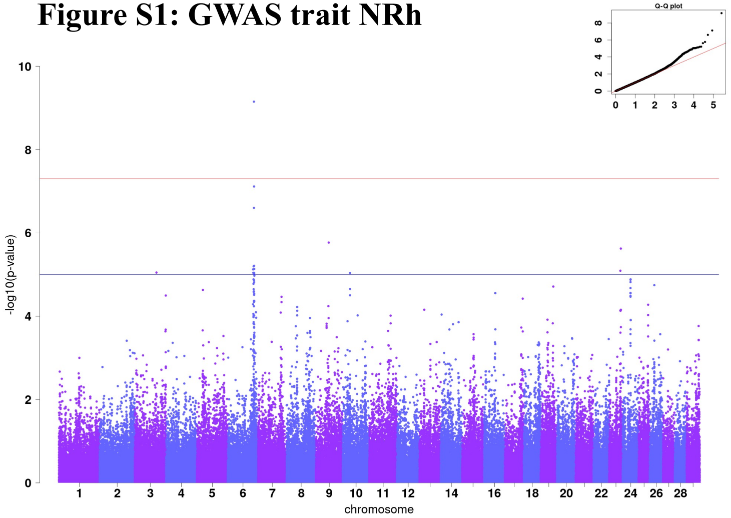

Figure S3: GWAS trait IFLh

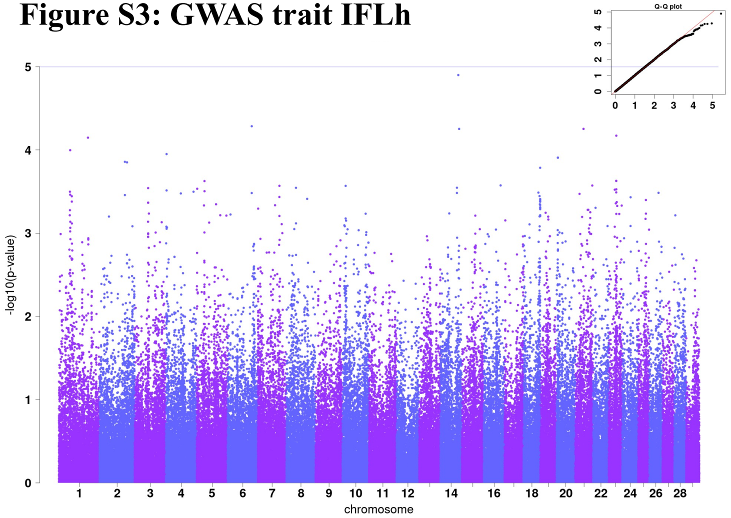

Figure S2: GWAS trait NRc

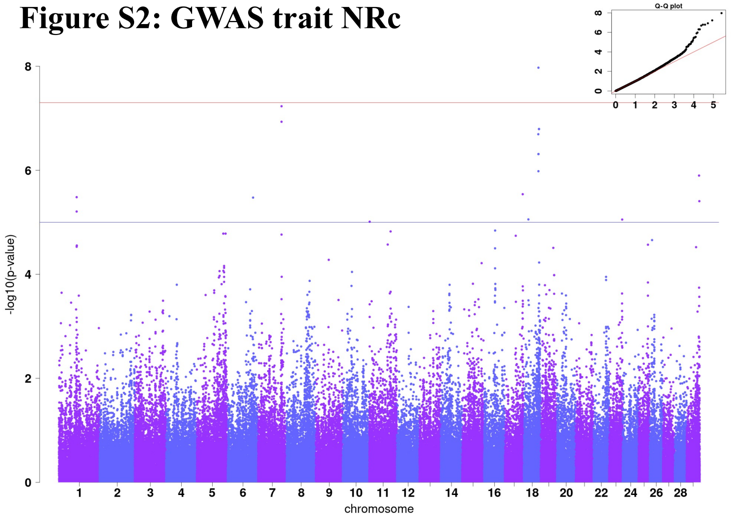

Figure S4: GWAS trait IFLc

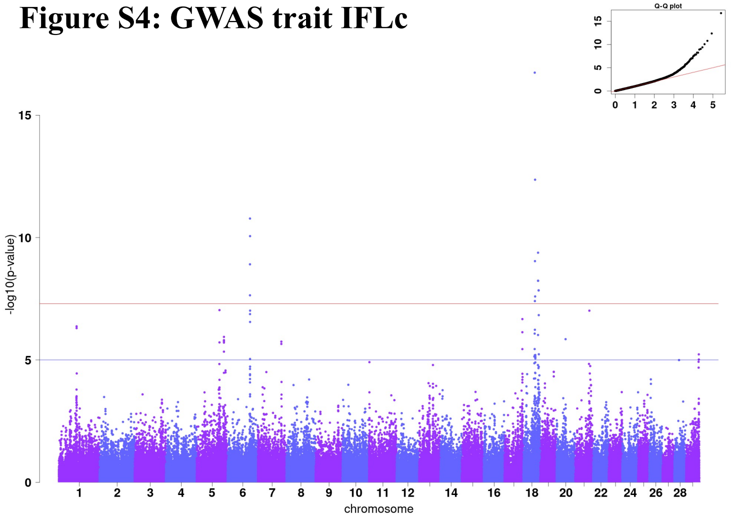

Figure S5: GWAS trait DFS

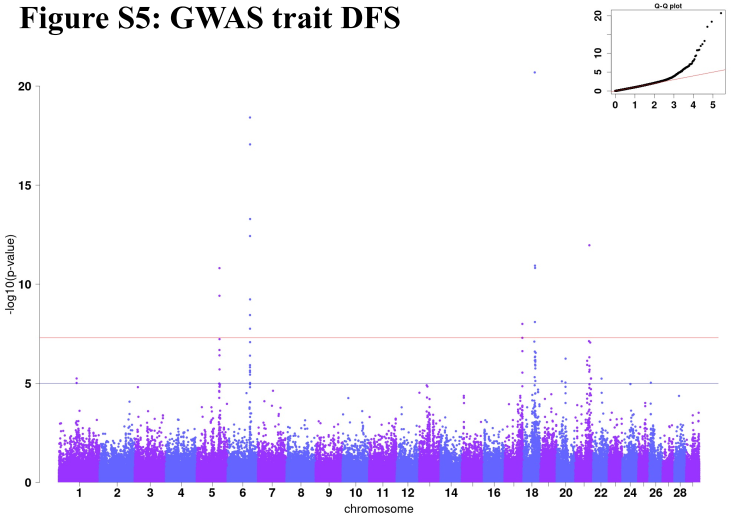

HO birth traits

Figure S6: GWAS trait CEd

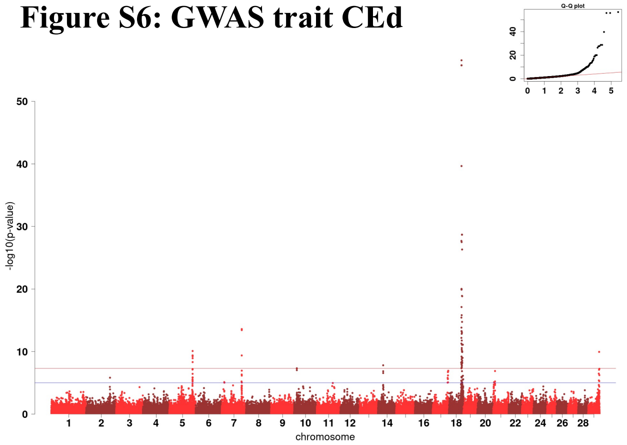

Figure S8: GWAS trait SBd

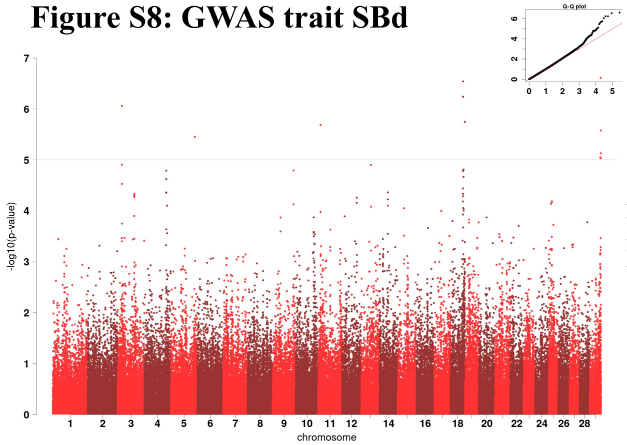

Figure S10: GWAS trait BWd

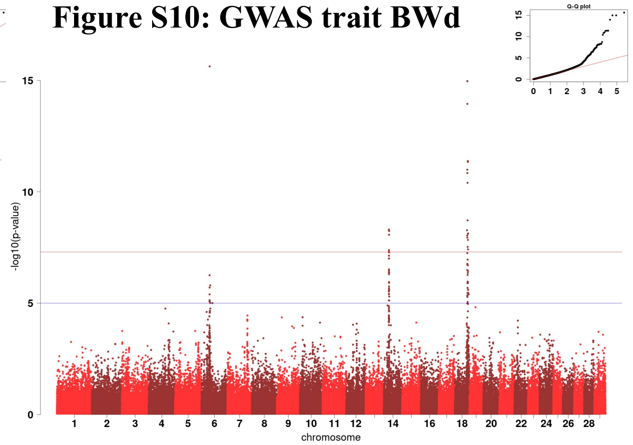

Figure S12: GWAS trait GLd

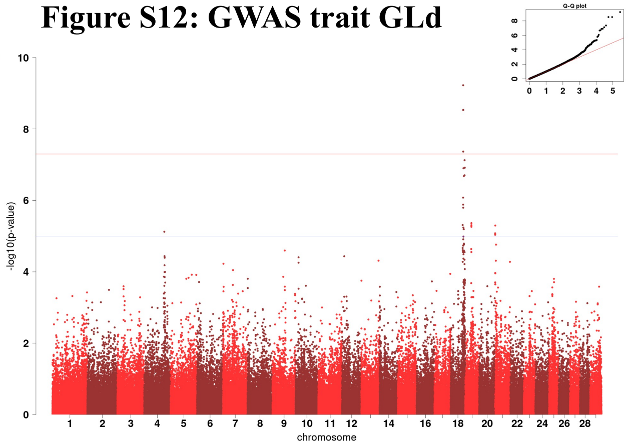

Figure S7: GWAS trait CEm

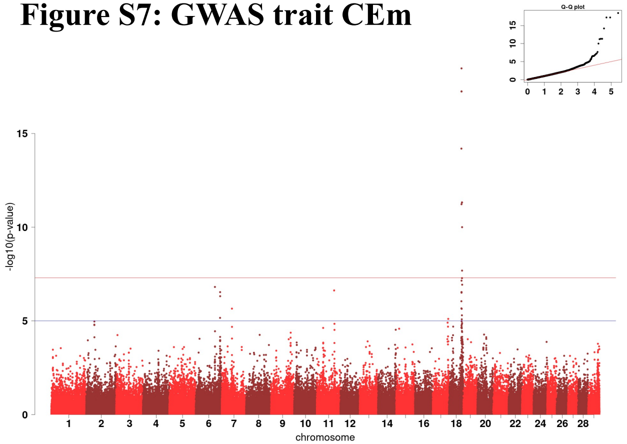

Figure S9: GWAS trait SBm

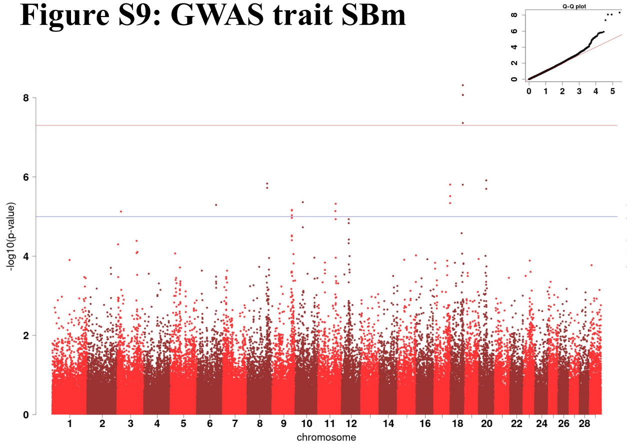

Figure S11: GWAS trait BWm

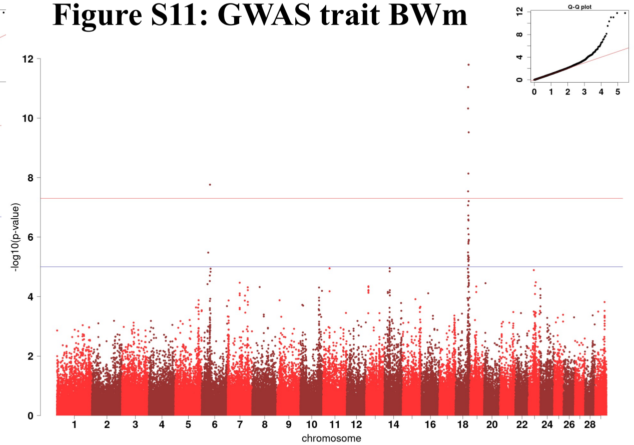

Figure S13: GWAS trait GLm

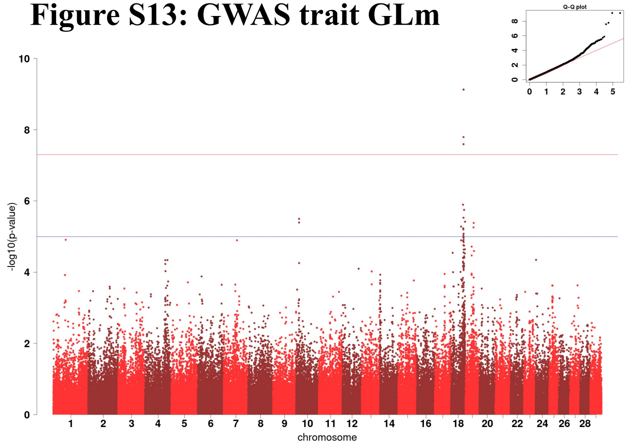

HO birth traits

Figure S14: GWAS trait MBd

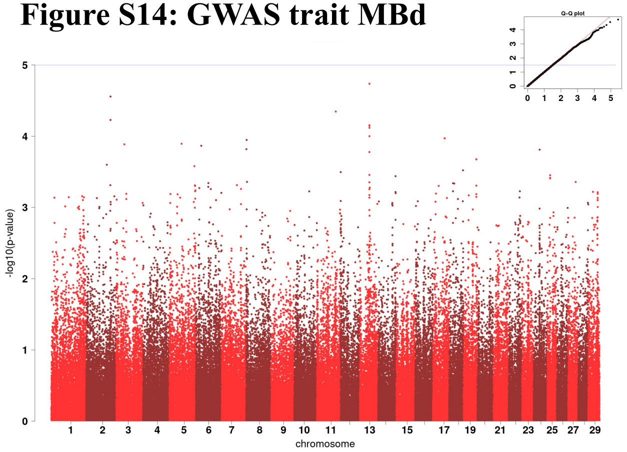

Figure S15: GWAS trait MBm

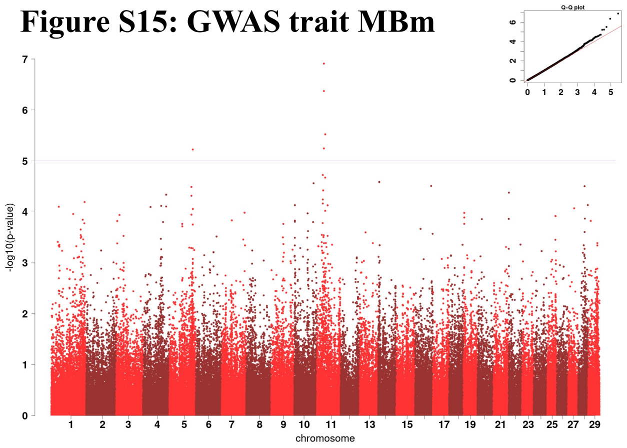

HO survival traits

Figure S16: GWAS trait P1

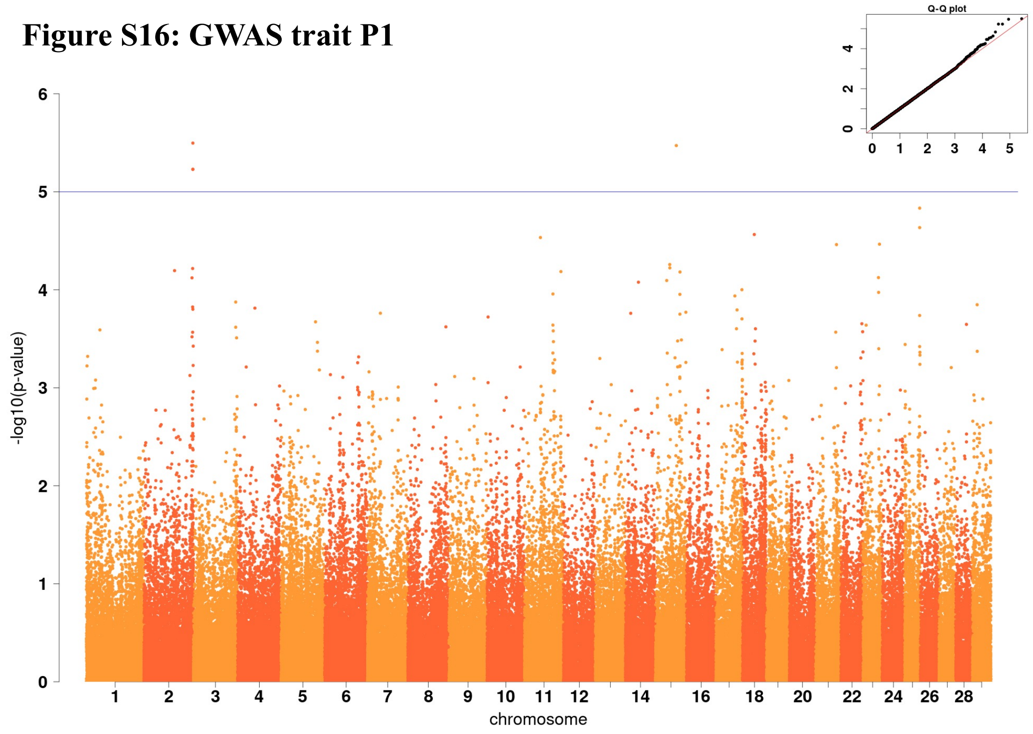

Figure S17: GWAS trait P1b

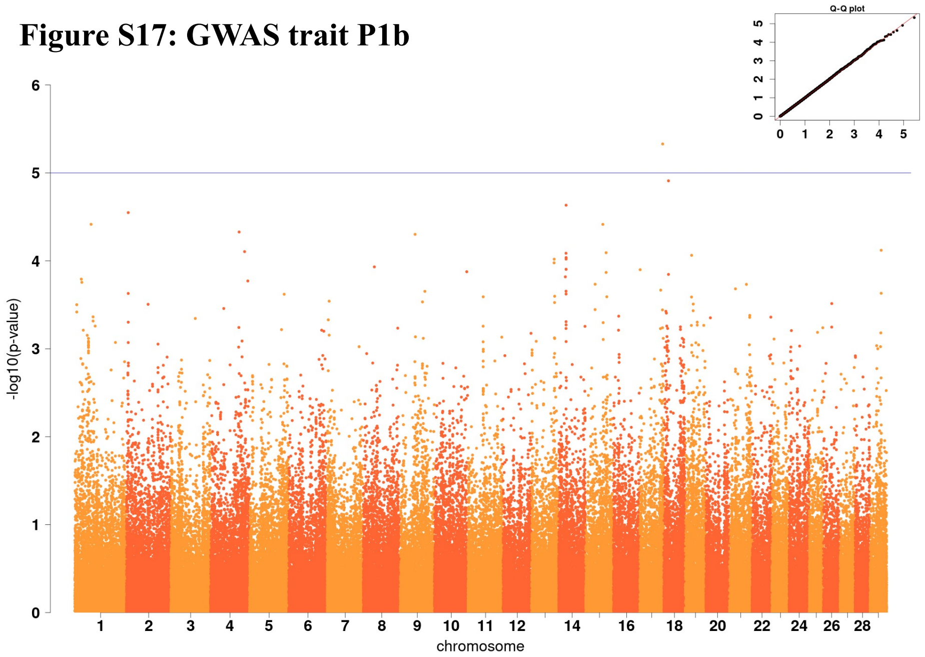

Figure S18: GWAS trait P1h

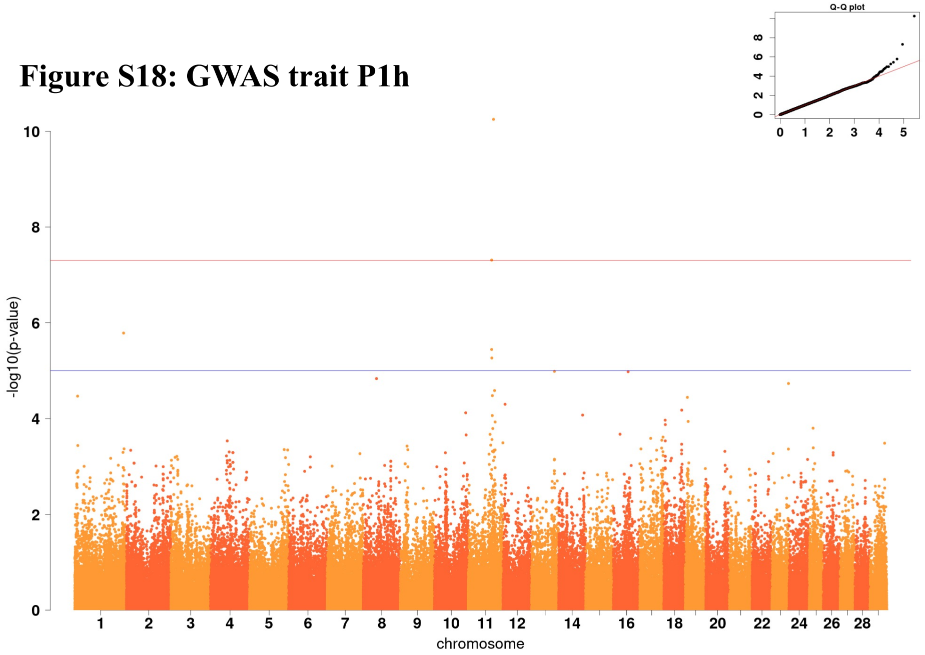

Figure S19: Screenshot of the genomic region surrounding the RIOX1 variant g.10:84938370:CTGGTGGAGGCGCAGACCCCGGCGGCACGCT_C, including genomes to be predicted homozygous wildtype, heterozygous variant and homozygous variant.

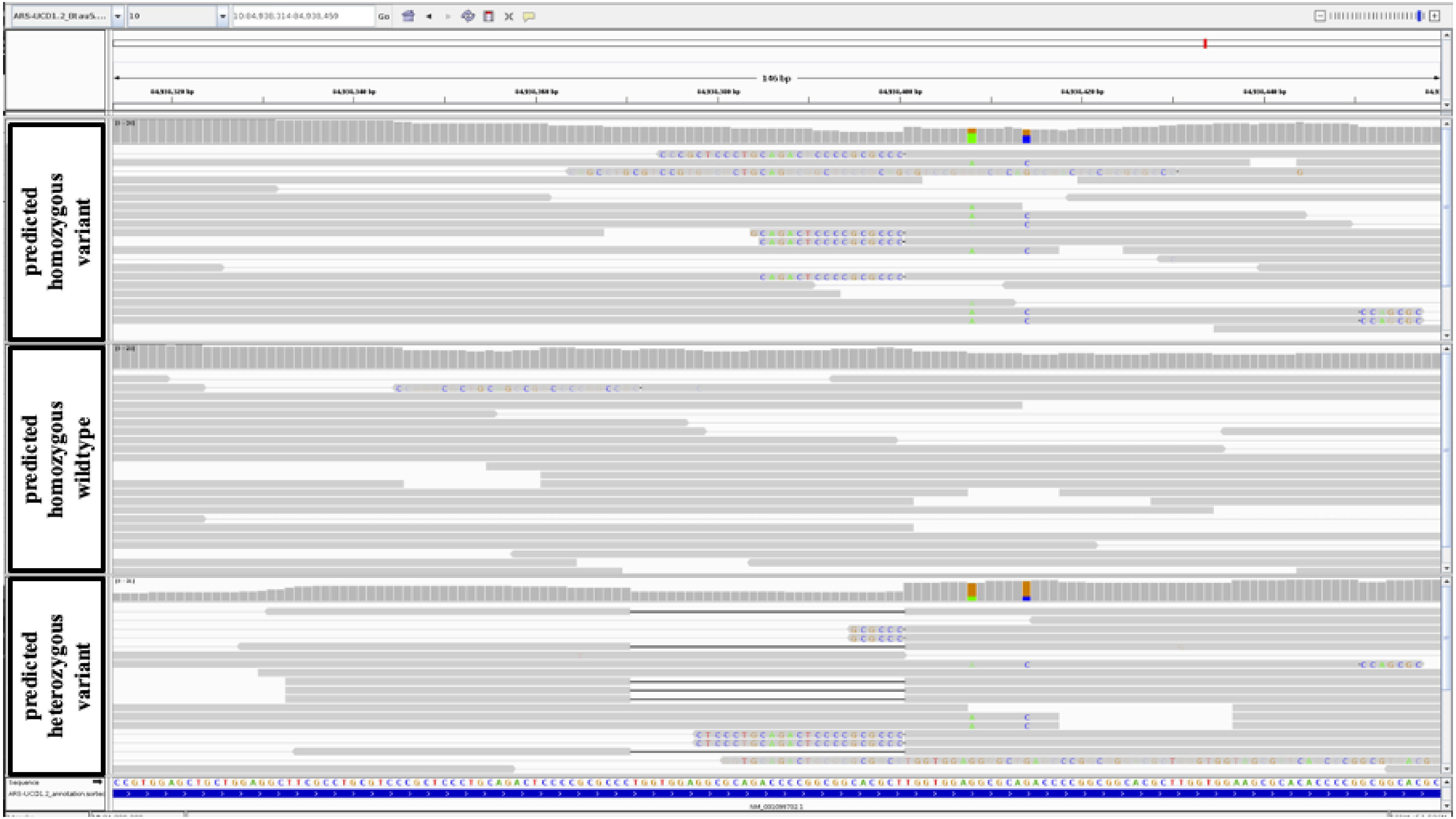

Supplementary Table S1: Previously identified haplotypes in different Holstein populations.

population first detected in	haplotype name	alternative name	associated disorder	chr	genomic region [a]	associated gene	OMIA / OMIM	publications
Danish	HH0	HBY (Fritz et al., 2018); 21-276, 21-301, 21-326 (Sahana et al., 2013)	brachyspina	21	20.1-22.6Mb	*FANCI*	000151-9913 / 611360	Agerholm et al., 2006; Charlier et al., 2012; Sahana et al., 2013; Cole et al., 2018; Fritz et al., 2018; Wu et al., 2019; Hozé et al., 2020
American	HH1	133.74 (VanRaden et al., 2011)	embryonic lethality	5	58-66Mb	*APAF1*	000001-9913 / 602233	VanRaden et al., 2011; Adams et al., 2016; Cole et al., 2018; Fritz et al., 2018; Hozé et al., 2020
American	HH2	21.337 (VanRaden et al., 2011)	abortion	1	92-97Mb	*IFT80*	001823-9913	VanRaden et al., 2011; McClure et al., 2014; Cole et al., 2018; Ortega et al. 2021
American	HH3	218.61 (VanRaden et al., 2011); 08-1276, 08-1301, 08-1326, 08-1351 (Sahana et al., 2013)	abortion	8	90-95Mb	*SMC2*	001824-9913 / 605576	VanRaden et al., 2011; Sahana et al., 2013; McClure et al., 2014; Cole et al., 2018; Wu et al., 2019; Hozé et al., 2020

American	175.5	07-126 (Sahana et al., 2013); HH20 (this study)	abortion	7	3-9Mb		001908-9913	VanRaden et al., 2011; Sahana et al., 2013
American	369.1		abortion	15	72-76Mb			VanRaden et al., 2011
French	HH4		abortion	1	0.1-3.3Mb	*GART*	001826-9913 / 138440	Fritz et al., 2013; Cole et al., 2018; Hozé et al., 2020
American	HH5		abortion	9	94.0-96.5Mb	*TFB1M*	001941-9913 / 607033	Schütz et al., 2016; Cole et al., 2018; Fritz et al., 2018; Hozé et al., 2020
German	CDH		cholesterol deficiency; juvenile mortality	11	77.3-78.2Mb	*APOB*	001965-9913 / 107730	Kipp et al., 2016; Menzi et al., 2016; Schütz et al., 2016; Cole et al., 2018
American	HHB		bovine leukocyte adhesion deficiency	1	139-145Mb	*ITGB2*	000595-9913 / 600065	Shuster et al., 1992; Cole et al., 2018
Danish	HHC	HH6 (Fritz et al., 2013)	complex vertebral malformation	3	42-44Mb-52.6Mb	*SLC35A3*	001340-9913/ 605632	Agerholm et al., 2001; Thomsen et al., 2006; Fritz et al., 2013; Cole et al., 2018
American	HHD		Deficiency of uridine monophosphate synthase	1	55-67Mb	*UMPS*	000262-9913 / 613891	Shanks et al., 1984; Schwenger et al., 1993; Cole et al., 2018
French	HH6		embryonic lethality	16	26-31.9Mb	*SDE2*	002149-9913	Fritz et al., 2018; Hozé et al., 2020
French	HH7		embryonic lethality	27	13.0-14.3Mb	*CENPU*	001830-9913 / 611511	Hozé et al., 2020
French	HH7		reduced homozygosity	6	51.6-52.6Mb			Fritz et al., 2013
French	HH8		reduced homozygosity	7	76.6-77.8Mb		001831-9913	Fritz et al., 2013
French	HH9		reduced homozygosity	10	74.5-76.7Mb		001832-9913	Fritz et al., 2013
French	HH10		reduced homozygosity	11	31.6-33.4Mb		001834-9913	Fritz et al., 2013

French	HH11	reduced homozygosity	12	20.5-36.4Mb	001833-9913	Fritz et al., 2013
French	HH12	reduced homozygosity	15	76.5-78.3Mb	001835-9913	Fritz et al., 2013
French	HH13	reduced homozygosity	18	55.9-59.2Mb	001836-9913	Fritz et al., 2013
French	HH14	reduced homozygosity	19	42.1-43.3Mb	001837-9913	Fritz et al., 2013
French	HH15	reduced homozygosity	20	58.4-59.5Mb	001838-9913	Fritz et al., 2013
French	HH16	reduced homozygosity	26	10.4-12.8Mb	001839-9913	Fritz et al., 2013
French	HH17	reduced homozygosity	26	25.3-26.4Mb	001840-9913	Fritz et al., 2013
Nordic	05-826	reduced homozygosity	5	66.4-68.3Mb	001906-9913	Sahana et al., 2013
Nordic	05-1351	reduced homozygosity	5	106.2-107.1Mb	001907-9913	Sahana et al., 2013
Nordic	05-1476	reduced homozygosity	5	112.1-113.7Mb	001907-9913	Sahana et al., 2013
Nordic	07-501	reduced homozygosity	7	33.3-34.8Mb	001909-9913	Sahana et al., 2013
Nordic	11-926	reduced homozygosity	11	55.4-57.4Mb	001910-9913	Sahana et al., 2013
Nordic	11-976	reduced homozygosity	11	59.2-60.6Mb	001910-9913	Sahana et al., 2013
Nordic	11-1001	reduced homozygosity	11	60.7-62.2Mb	001910-9913	Sahana et al., 2013
Nordic	11-1026	reduced homozygosity	11	62.2-63.8Mb	001910-9913	Sahana et al., 2013
Nordic	19-151	reduced homozygosity	19	12.8-14.2Mb	001911-9913	Sahana et al., 2013
Nordic	02-36	embryonic lethality	2	35.4-36.1Mb		Wu et al., 2019
Nordic	05-7	embryonic lethality	5	6.9-7.8Mb		Wu et al., 2019
Nordic	03-70	absence of homozygosity	3	70.3-70.8Mb		Wu et al., 2019
Nordic	06-0	absence of homozygosity	6	0.4-0.8Mb		Wu et al., 2019
Nordic	09-24	absence of homozygosity	9	23.9-25.0Mb		Wu et al., 2019
Nordic	09-58	absence of homozygosity	9	57.6-58.6Mb		Wu et al., 2019
Nordic	10-27	absence of homozygosity	10	26.4-27.6Mb		Wu et al., 2019
Nordic	15-47	absence of homozygosity	15	47.1-47.5Mb		Wu et al., 2019

Nordic	17-33	absence of homozygosity	17	32.6-33.0Mb	Wu et al., 2019

[a] according to the reference sequence ARS-UCD1.2 (National Center for Biotechnology Information, 2018a)

Supplementary Table S2: Sample ids from whole-genome sequencing samples stored in the European Nucleotide Archive.

EBI Sample ID	avCov	insertSize	breed
SAMEA5159765	10.3	347.3	Simmental
SAMEA5159766	10.2	372.6	Simmental
SAMEA5159767	11.0	361.4	Original Braunvieh
SAMEA5159768	10.9	387.0	Original Braunvieh
SAMEA5159769	12.6	328.3	Brown Swiss
SAMEA5159770	10.2	352.2	Brown Swiss
SAMEA5159771	11.1	336.8	Brown Swiss
SAMEA5159772	12.2	338.4	Brown Swiss
SAMEA5159773	11.1	349.9	Brown Swiss
SAMEA5159774	11.8	344.4	Brown Swiss
SAMEA5159775	11.9	356.3	Brown Swiss
SAMEA5159776	11.1	347.6	Original Braunvieh
SAMEA5159777	11.4	320.9	Brown Swiss
SAMEA5159778	14.0	321.9	Brown Swiss
SAMEA5159779	11.3	333.1	Brown Swiss
SAMEA5159780	13.1	309.1	Brown Swiss
SAMEA5159781	13.3	356.2	Brown Swiss
SAMEA5159782	11.9	365.1	Brown Swiss
SAMEA5159783	12.4	364.9	Brown Swiss
SAMEA5159784	12.5	377.6	Brown Swiss
SAMEA5159785	12.5	351.1	Brown Swiss
SAMEA5159786	12.5	343.8	Brown Swiss
SAMEA5159787	14.5	369.9	Brown Swiss
SAMEA5159788	11.5	363.0	Brown Swiss
SAMEA5159789	10.9	359.9	Original Braunvieh
SAMEA5159790	11.2	361.9	Original Braunvieh
SAMEA5159791	10.8	373.2	Brown Swiss
SAMEA5159792	11.3	366.6	Brown Swiss
SAMEA5159793	12.7	356.7	Brown Swiss
SAMEA5159794	12.3	382.8	Original Braunvieh
SAMEA5159795	13.6	372.7	Original Braunvieh
SAMEA5159796	12.2	375.5	Original Braunvieh
SAMEA5159797	12.9	343.3	Brown Swiss
SAMEA5159798	14.0	370.1	Brown Swiss
SAMEA5159799	10.8	366.9	Brown Swiss
SAMEA5159800	14.2	357.8	Simmental
SAMEA5159801	13.5	374.5	Simmental
SAMEA5159802	13.5	367.2	Holstein
SAMEA5159803	12.1	372.2	Simmental
SAMEA5159804	13.1	389.2	Holstein
SAMEA5159805	11.6	357.6	Simmental
SAMEA5159806	11.0	382.2	Simmental
SAMEA5159807	11.4	384.4	Simmental

SAMEA5159808	11.7	376.2	Holstein
SAMEA5159809	11.6	354.2	Holstein
SAMEA5159810	11.8	368.0	Holstein
SAMEA5159811	11.9	365.4	Simmental
SAMEA5159812	11.9	378.1	Simmental
SAMEA5159813	11.2	376.1	Holstein
SAMEA5159814	12.2	374.5	Holstein
SAMEA5159815	12.8	374.9	Holstein
SAMEA5159816	11.7	382.2	Holstein
SAMEA5159817	11.4	352.8	Holstein
SAMEA5159818	11.9	359.5	Holstein
SAMEA5159819	12.8	364.5	Holstein
SAMEA5159820	11.5	357.3	Holstein
SAMEA5159821	11.4	376.3	Holstein
SAMEA5159822	12.1	379.3	Holstein
SAMEA5159823	11.9	386.1	Simmental
SAMEA5159824	10.2	377.5	Simmental
SAMEA5159825	11.8	349.2	Simmental
SAMEA5159826	10.2	349.5	Holstein
SAMEA5159827	10.7	359.5	Simmental
SAMEA5159828	12.1	342.0	Holstein
SAMEA5159760	17.4	266.5	Simmental
SAMEA5159761	17.5	240.1	Brown Swiss
SAMEA3706825	10.8	404.8	Angus
SAMEA5415491	19.5	388.3	Belgium Blue
SAMEA4051550	13.2	271.5	Galloway
SAMEA4644752	18.5	406.3	Shorthorn
SAMEA8565029	16.2	313.7	Original Braunvieh
SAMEA4644757	14.3	397.4	Brown Swiss
SAMEA4644727	18.2	387.7	Brown Swiss
SAMEA4644734	18.9	397.5	Original Braunvieh
SAMEA5714976	21.1	309.9	Brown Swiss
SAMEA19315168	15.9	453.6	Brown Swiss
SAMEA5415485	20.5	383.0	Brown Swiss
SAMEA6528890	17.1	400.7	Original Braunvieh
SAMEA4644764	13.7	419.4	Original Braunvieh
SAMEA5714974	25.5	491.1	Original Braunvieh
SAMEA8565036	21.5	350.5	Brown Swiss
SAMEA5159847	26.1	448.7	Brown Swiss
SAMEA5159886	37.0	483.1	Original Braunvieh
SAMEA4644728	18.5	407.2	Brown Swiss
SAMEA4644749	17.5	397.1	Original Braunvieh
SAMEA6528886	23.5	393.5	Original Braunvieh
SAMEA8565035	23.3	350.1	Original Braunvieh
SAMEA8565034	21.0	337.3	Original Braunvieh
SAMEA4644750	18.6	352.6	Original Braunvieh
SAMEA6528894	18.4	404.2	Original Braunvieh
SAMEA8565033	21.0	329.0	Brown Swiss

SAMEA6528892	20.7	386.9	Original Braunvieh
SAMEA4644762	18.2	430.7	Brown Swiss
SAMEA4644739	17.4	401.6	Brown Swiss
SAMEA4644755	17.3	406.2	Brown Swiss
SAMEA5415486	24.2	375.9	Brown Swiss
SAMEA5564716	16.8	442.6	Brown Swiss
SAMEA5564728	19.8	368.0	Original Braunvieh
SAMEA19312918	11.1	399.3	Brown Swiss
SAMEA5415489	23.0	428.1	Brown Swiss
SAMEA4644763	14.6	392.8	Brown Swiss
SAMEA4644766	21.4	415.7	Brown Swiss
SAMEA19323418	28.9	414.2	Brown Swiss
SAMEA5714979	28.0	453.6	Brown Swiss
SAMEA6528889	18.1	392.4	Original Braunvieh
SAMEA19313668	17.2	399.0	Brown Swiss
SAMEA4644769	21.8	460.9	Brown Swiss
SAMEA8565098	21.2	337.2	Brown Swiss
SAMEA19314418	17.7	447.3	Brown Swiss
SAMEA4644754	19.1	393.7	Brown Swiss
SAMEA5159853	31.7	467.9	Brown Swiss
SAMEA4644765	16.0	413.8	Brown Swiss
SAMEA4644768	17.4	429.4	Original Braunvieh
SAMEA5415488	22.9	360.4	Brown Swiss
SAMEA6528891	23.6	381.6	Original Braunvieh
SAMEA8565097	21.0	346.7	Brown Swiss
SAMEA5714971	20.1	377.6	Brown Swiss
SAMEA8565096	14.7	416.1	Original Braunvieh
SAMEA6528888	17.1	370.1	Original Braunvieh
SAMEA4644741	19.4	405.1	Original Braunvieh
SAMEA4827664	12.6	405.1	Original Braunvieh
SAMEA4827655	12.7	426.7	Original Braunvieh
SAMEA4644743	19.3	408.9	Brown Swiss
SAMEA4827671	12.4	433.2	Original Braunvieh
SAMEA4827649	13.1	425.8	Original Braunvieh
SAMEA5159875	22.8	440.7	Brown Swiss
SAMEA4827653	10.8	425.9	Original Braunvieh
SAMEA4827657	9.4	426.2	Original Braunvieh
SAMEA4827663	12.5	410.4	Original Braunvieh
SAMEA4827661	10.8	418.4	Original Braunvieh
SAMEA4827662	19.2	399.8	Original Braunvieh
SAMEA4827658	10.9	429.3	Original Braunvieh
SAMEA7690216	17.4	335.3	Original Braunvieh
SAMEA4827646	14.6	417.7	Original Braunvieh
SAMEA4827659	10.7	420.3	Original Braunvieh
SAMEA4827652	11.8	421.4	Original Braunvieh
SAMEA4827656	13.2	425.3	Original Braunvieh
SAMEA4827669	14.0	427.1	Original Braunvieh
SAMEA4827651	16.5	426.1	Original Braunvieh

SAMEA4827645	16.3	418.2	Original Braunvieh
SAMEA4827660	11.2	414.1	Original Braunvieh
SAMEA4827650	11.7	421.2	Original Braunvieh
SAMEA4644758	16.4	392.7	Brown Swiss
SAMEA4827666	12.2	423.1	Original Braunvieh
SAMEA5159861	24.9	448.6	Brown Swiss
SAMEA5159862	27.0	443.4	Brown Swiss
SAMEA4827647	16.7	420.0	Original Braunvieh
SAMEA4827674	13.7	416.9	Original Braunvieh
SAMEA4827667	12.0	426.1	Original Braunvieh
SAMEA5059742	3.6	426.4	Original Braunvieh
SAMEA4827654	12.0	426.6	Original Braunvieh
SAMEA4827665	14.1	417.6	Original Braunvieh
SAMEA4827670	13.3	428.9	Original Braunvieh
SAMEA4644740	19.5	397.3	Original Braunvieh
SAMEA4644742	19.4	391.6	Brown Swiss
SAMEA4827648	12.2	426.4	Original Braunvieh
SAMEA4827668	11.8	423.5	Original Braunvieh
SAMEA4827673	11.3	423.0	Original Braunvieh
SAMEA5159849	27.0	448.7	Original Braunvieh
SAMEA4827672	11.4	433.0	Original Braunvieh
SAMEA5159867	26.5	446.3	Brown Swiss
SAMEA5059748	4.8	419.9	Original Braunvieh
SAMEA19318918	11.5	407.6	Brown Swiss
SAMEA5159869	27.6	451.8	Brown Swiss
SAMEA6272108	21.0	315.7	Brown Swiss
SAMEA5159870	32.6	451.3	Brown Swiss
SAMEA5059741	4.4	431.8	Original Braunvieh
SAMEA5415498	21.9	367.3	Brown Swiss
SAMEA8565028	20.9	328.7	Brown Swiss
SAMEA5059750	3.7	424.8	Original Braunvieh
SAMEA5059751	5.3	427.5	Original Braunvieh
SAMEA5059747	4.4	414.7	Original Braunvieh
SAMEA5059759	3.8	428.7	Original Braunvieh
SAMEA5159850	28.4	453.0	Original Braunvieh
SAMEA5059743	21.4	423.6	Original Braunvieh
SAMEA5059754	3.3	420.3	Original Braunvieh
SAMEA5059749	5.0	429.5	Original Braunvieh
SAMEA5159872	28.0	449.3	Brown Swiss
SAMEA5059758	4.9	422.8	Original Braunvieh
SAMEA5059752	3.7	425.3	Original Braunvieh
SAMEA5059745	4.1	429.1	Original Braunvieh
SAMEA5159885	25.3	487.6	Brown Swiss
SAMEA5059757	5.5	420.7	Original Braunvieh
SAMEA5059746	4.1	424.2	Original Braunvieh
SAMEA5159873	24.2	447.9	Brown Swiss
SAMEA5059756	6.4	409.4	Original Braunvieh
SAMEA5059744	3.8	424.9	Original Braunvieh

SAMEA5059755	7.2	417.1	Original Braunvieh
SAMEA4644756	18.0	416.5	Brown Swiss
SAMEA5159871	34.4	449.8	Brown Swiss
SAMEA5059753	4.0	424.6	Original Braunvieh
SAMEA5159848	23.0	450.5	Original Braunvieh
SAMEA6528893	14.2	398.1	Original Braunvieh
SAMEA5159868	26.8	455.9	Brown Swiss
SAMEA4644730	18.0	384.2	Original Braunvieh
SAMEA5159837	29.5	491.6	Original Braunvieh
SAMEA5159843	23.9	499.1	Original Braunvieh
SAMEA5714972	32.2	443.6	Original Braunvieh
SAMEA7015108	17.9	379.0	Original Braunvieh
SAMEA6528895	15.1	396.8	Original Braunvieh
SAMEA5714975	22.6	382.8	Original Braunvieh
SAMEA6528887	21.0	323.7	Original Braunvieh
SAMEA5564726	19.2	375.0	Original Braunvieh
SAMEA5564727	19.0	378.1	Original Braunvieh
SAMEA5415490	22.1	367.5	Brown Swiss
SAMEA5159874	33.7	450.6	Brown Swiss
SAMEA5159865	26.0	434.6	Brown Swiss
SAMEA5159863	23.7	441.2	Brown Swiss
SAMEA5159866	25.1	441.4	Brown Swiss
SAMEA4644735	15.1	375.4	Brown Swiss
SAMEA5159864	25.0	447.7	Brown Swiss
SAMEA19846918	14.7	313.8	Holstein
SAMEA7690217	17.6	342.5	Charolais
SAMEA33668668	19.3	412.4	Chianina
SAMEA7690197	18.2	346.4	Chianina
SAMEA7690231	17.7	357.9	Chianina
SAMEA5159835	38.4	456.5	Chianina
SAMEA8565052	20.7	351.2	Chianina
SAMEA8565051	21.5	334.3	Chianina
SAMEA8565050	22.5	349.1	Chianina
SAMEA7690198	17.9	349.4	Chianina
SAMEA32999668	21.6	414.4	Chianina
SAMEA7690230	19.3	363.2	Chianina
SAMEA8565023	20.9	329.6	Chianina
SAMEA32998168	29.1	394.0	Chianina
SAMEA7690196	17.3	338.8	Belgian Blue x Holstein
SAMEA32989918	8.4	304.6	Cika
SAMEA4560538	22.1	259.7	Eringer
SAMEA19847668	19.3	262.9	Brown Swiss
SAMEA19848418	15.8	276.3	Holstein
SAMEA19849168	11.4	392.6	Eringer
SAMEA5714967	28.6	471.2	Eringer
SAMEA6528879	16.4	386.9	Eringer
SAMEA6272106	16.0	312.0	Brown Swiss
SAMEA6272107	9.7	252.4	Original Braunvieh

SAMEA6272109	7.5	259.8	Original Braunvieh
SAMEA6272110	7.5	256.4	Original Braunvieh
SAMEA6272111	29.8	334.2	Brown Swiss
SAMEA6272112	8.9	254.2	Original Braunvieh
SAMEA6272113	9.9	256.9	Original Braunvieh
SAMEA6272114	7.6	256.6	Original Braunvieh
SAMEA6272115	8.1	257.6	Original Braunvieh
SAMEA6272116	72.8	366.3	Brown Swiss
SAMEA6272118	19.1	389.3	Original Braunvieh
SAMEA6272119	20.1	390.4	Original Braunvieh
SAMEA6272120	19.0	396.5	Original Braunvieh
SAMEA6272121	17.0	389.5	Original Braunvieh
SAMEA6272122	18.8	397.0	Original Braunvieh
SAMEA6272123	17.3	384.6	Original Braunvieh
SAMEA6272124	19.2	395.6	Original Braunvieh
SAMEA6272125	23.9	423.3	Original Braunvieh
SAMEA6272126	63.7	382.5	Original Braunvieh
SAMEA6272127	55.7	436.8	Original Braunvieh
SAMEA6163175	11.3	489.5	Brown Swiss
SAMEA6163176	11.6	493.5	Brown Swiss
SAMEA6163177	10.5	483.2	Brown Swiss
SAMEA6163178	7.2	481.1	Brown Swiss
SAMEA6163179	8.8	476.5	Brown Swiss
SAMEA6163180	11.4	482.4	Brown Swiss
SAMEA6163181	10.0	497.5	Brown Swiss
SAMEA6163182	10.4	511.5	Brown Swiss
SAMEA6163183	9.9	499.7	Brown Swiss
SAMEA6163184	9.9	512.4	Brown Swiss
SAMEA6163186	17.6	297.3	Brown Swiss
SAMEA6163187	27.2	341.0	Brown Swiss
SAMEA6163188	33.4	354.7	Brown Swiss
SAMEA6163189	23.4	339.1	Brown Swiss
SAMEA6163190	16.1	322.0	Brown Swiss
SAMEA6163191	15.7	320.9	Brown Swiss
SAMEA6163192	15.4	299.9	Brown Swiss
SAMEA6163193	17.5	319.5	Brown Swiss
SAMEA6163194	17.4	312.9	Brown Swiss
SAMEA6163195	22.1	327.7	Brown Swiss
SAMEA6272082	19.0	436.8	Original Braunvieh
SAMEA6272083	33.2	488.7	Original Braunvieh
SAMEA6272084	12.2	423.5	Original Braunvieh
SAMEA6272085	7.6	260.2	Original Braunvieh
SAMEA6272086	11.8	257.2	Original Braunvieh
SAMEA6272087	10.9	253.5	Original Braunvieh
SAMEA6272088	10.3	259.3	Original Braunvieh
SAMEA6272089	7.7	253.5	Original Braunvieh
SAMEA6272090	10.0	255.9	Original Braunvieh
SAMEA6272091	9.0	258.0	Original Braunvieh

SAMEA6272092	8.8	251.0	Original Braunvieh
SAMEA6272093	10.6	258.4	Original Braunvieh
SAMEA6272094	7.8	257.0	Original Braunvieh
SAMEA6272095	26.1	318.3	Brown Swiss
SAMEA6272096	21.0	328.9	Brown Swiss
SAMEA6272097	11.0	317.7	Brown Swiss
SAMEA6272098	15.4	309.0	Brown Swiss
SAMEA6272099	12.6	335.3	Brown Swiss
SAMEA6272100	8.1	257.9	Original Braunvieh
SAMEA6272101	10.6	321.4	Brown Swiss
SAMEA6272102	6.8	261.1	Original Braunvieh
SAMEA6272103	8.6	252.5	Original Braunvieh
SAMEA6272104	13.0	305.0	Brown Swiss
SAMEA6272105	34.8	326.2	Brown Swiss
SAMEA5714968	22.2	444.3	Evolène
SAMEA5714969	22.9	444.8	Evolène
SAMEA7015107	10.6	387.8	Evolène
SAMEA4051548	14.5	252.5	Brown Swiss
SAMEA19849918	15.8	250.4	Scotish Highland
SAMEA7015111	17.3	385.9	Hereford
SAMEA5159890	27.8	470.3	Holstein
SAMEA8565093	21.8	342.0	Holstein
SAMEA8565094	20.7	325.4	Holstein
SAMEA8565095	22.1	347.8	Holstein
SAMEA7690215	20.0	337.7	Holstein
SAMEA3682654	14.7	409.0	Holstein
SAMEA8565032	22.7	336.3	Swiss Fleckvieh
SAMEA8565030	21.7	340.0	Holstein
SAMEA4644737	17.4	399.6	Holstein
SAMEA7690214	17.7	342.3	Holstein
SAMEA4644729	18.8	399.7	Holstein
SAMEA7690232	18.6	310.0	Holstein
SAMEA4644732	18.3	397.0	Holstein
SAMEA4644731	17.6	401.3	Holstein
SAMEA19325668	22.7	402.9	Holstein
SAMEA5159854	33.3	467.4	Holstein
SAMEA6528904	23.1	383.9	Holstein
SAMEA6528896	11.8	433.8	Swiss Fleckvieh
SAMEA4644746	20.4	405.9	Holstein
SAMEA8565016	24.7	460.1	Holstein
SAMEA4644736	20.9	389.7	Holstein
SAMEA5415500	18.4	368.0	Holstein
SAMEA4560543	9.5	258.5	Holstein
SAMEA5159879	27.7	446.2	Holstein
SAMEA4644753	17.0	390.7	Holstein
SAMEA3682653	17.0	417.8	Holstein
SAMEA5415483	30.9	376.1	Holstein
SAMEA5415484	24.1	364.4	Holstein

SAMEA4644738	17.9	385.4	Holstein
SAMEA7690213	17.3	301.2	Holstein
SAMEA7015110	21.1	375.0	Holstein
SAMEA5159857	25.6	466.5	Holstein
SAMEA4644748	17.1	395.7	Holstein
SAMEA8565005	25.0	465.8	Holstein
SAMEA5159855	39.2	461.0	Holstein
SAMEA19309918	20.7	415.9	Holstein
SAMEA5415503	24.3	445.9	Holstein
SAMEA5159859	25.6	433.3	Holstein
SAMEA5159860	17.6	425.0	Holstein
SAMEA4644733	18.7	391.2	Holstein
SAMEA4644760	14.4	441.4	Holstein
SAMEA8565025	20.8	337.4	Holstein
SAMEA5159840	26.9	483.7	Holstein
SAMEA5415501	19.6	377.3	Holstein
SAMEA5415502	15.1	370.8	Holstein
SAMEA19310668	17.0	414.4	Holstein
SAMEA8565027	35.3	331.2	Holstein
SAMEA6528905	24.7	379.3	Holstein
SAMEA7690265	16.8	315.1	Holstein
SAMEA4644761	17.6	408.2	Holstein
SAMEA7690212	17.6	342.8	Holstein
SAMEA5714977	20.8	314.1	Holstein
SAMEA5714978	17.2	311.0	Holstein
SAMEA8565064	21.6	351.2	Holstein
SAMEA7690259	17.6	318.0	Holstein
SAMEA8565067	21.4	349.0	Holstein
SAMEA7690256	17.7	305.5	Holstein
SAMEA7690258	21.3	343.6	Holstein
SAMEA8565079	24.0	344.0	Holstein
SAMEA8565040	22.5	354.9	Holstein
SAMEA8565071	22.1	360.7	Holstein
SAMEA8565058	20.2	334.7	Holstein
SAMEA7690254	18.3	309.6	Holstein
SAMEA7690261	19.8	305.0	Holstein
SAMEA7690253	17.1	300.4	Holstein
SAMEA8565076	21.1	345.2	Holstein
SAMEA7690255	18.4	319.6	Holstein
SAMEA8565091	16.1	319.5	Holstein
SAMEA8565061	22.6	347.7	Holstein
SAMEA7690260	18.1	308.5	Holstein
SAMEA8565073	21.1	333.6	Holstein
SAMEA7690257	17.3	315.5	Holstein
SAMEA19322668	13.8	432.4	Holstein
SAMEA7690264	16.9	293.9	Holstein
SAMEA7690262	16.8	282.6	Holstein
SAMEA8565080	19.7	333.7	Holstein

SAMEA8565038	21.2	351.9	Holstein
SAMEA7690266	19.4	287.0	Holstein
SAMEA8565042	22.5	325.4	Holstein
SAMEA8565074	22.5	332.6	Holstein
SAMEA7690267	17.9	284.3	Holstein
SAMEA8565092	21.3	329.1	Holstein
SAMEA8565041	22.4	340.8	Holstein
SAMEA8565062	20.6	327.5	Holstein
SAMEA8565057	20.0	363.0	Holstein
SAMEA8565077	24.2	324.1	Holstein
SAMEA8565084	22.3	336.8	Holstein
SAMEA8565065	22.2	336.7	Holstein
SAMEA8565059	14.4	322.9	Holstein
SAMEA7690234	17.8	348.2	Holstein
SAMEA7690223	17.8	343.8	Holstein
SAMEA6528907	23.8	380.3	Holstein
SAMEA6528908	18.8	372.1	Holstein
SAMEA7690200	18.5	339.1	Holstein
SAMEA7690238	18.4	302.3	Holstein
SAMEA7015113	22.3	376.6	Holstein
SAMEA7015115	17.1	385.3	Holstein
SAMEA8565014	20.0	467.2	Holstein
SAMEA8565000	22.8	446.2	Holstein
SAMEA6528909	22.1	366.1	Holstein
SAMEA33004168	16.5	399.9	Holstein
SAMEA5159878	31.4	446.7	Holstein
SAMEA5159884	25.6	450.7	Holstein
SAMEA7690239	18.1	297.8	Holstein
SAMEA7690235	22.0	332.3	Holstein
SAMEA7015112	17.6	384.5	Holstein
SAMEA7690228	18.4	338.9	Holstein
SAMEA19318168	16.2	452.5	Holstein
SAMEA6528906	27.0	380.4	Holstein
SAMEA19317418	16.9	459.6	Holstein
SAMEA5159842	23.5	460.8	Holstein
SAMEA8565009	23.1	470.1	Holstein
SAMEA7690201	19.6	332.8	Holstein
SAMEA7690263	17.2	277.2	Holstein
SAMEA8565053	22.1	342.9	Holstein
SAMEA33000418	23.4	401.7	Holstein
SAMEA5160152	28.1	439.2	Holstein
SAMEA5159839	28.3	470.3	Holstein
SAMEA5159841	33.1	449.5	Holstein
SAMEA5159844	26.5	480.6	Holstein
SAMEA6528899	16.0	395.8	Holstein
SAMEA7690208	17.4	350.1	Holstein
SAMEA7690210	19.4	348.5	Holstein
SAMEA7690236	17.8	329.9	Holstein

SAMEA7690237	19.6	274.4	Holstein
SAMEA7690241	17.6	284.2	Holstein
SAMEA7690245	18.7	288.2	Holstein
SAMEA7690249	19.6	307.6	Holstein
SAMEA7690251	17.4	364.2	Holstein
SAMEA7690252	18.0	310.6	Holstein
SAMEA8565060	16.6	262.1	Holstein
SAMEA8565063	20.5	338.0	Holstein
SAMEA8565085	20.8	320.0	Holstein
SAMEA19311418	12.6	418.8	Holstein
SAMEA19316668	14.9	458.5	Holstein
SAMEA19320418	22.7	401.5	Holstein
SAMEA19321168	14.5	330.3	Holstein
SAMEA19321918	11.9	425.0	Holstein
SAMEA4644759	17.8	425.4	Holstein
SAMEA4644751	12.3	422.6	Holstein
SAMEA7690222	18.1	311.8	Holstein
SAMEA7690242	19.3	298.4	Holstein
SAMEA7690243	18.5	304.6	Holstein
SAMEA7690244	19.8	322.2	Holstein
SAMEA7690246	20.2	334.7	Holstein
SAMEA7690247	18.9	326.2	Holstein
SAMEA7690248	20.4	328.2	Holstein
SAMEA8565068	22.2	339.1	Holstein
SAMEA8565069	22.5	329.6	Holstein
SAMEA8565070	25.7	345.8	Holstein
SAMEA8565075	20.6	288.2	Holstein
SAMEA8565078	22.0	301.0	Holstein
SAMEA8565081	21.7	349.7	Holstein
SAMEA8565082	22.3	306.3	Holstein
SAMEA8565086	19.6	326.2	Holstein
SAMEA8565089	22.5	296.4	Holstein
SAMEA19312168	10.6	406.1	Holstein
SAMEA8565026	17.0	306.3	Holstein
SAMEA5415492	18.3	376.8	Holstein
SAMEA5415494	16.8	379.8	Holstein
SAMEA5415496	17.9	363.6	Holstein
SAMEA8565039	19.8	322.6	Holstein
SAMEA8565056	21.8	322.7	Holstein
SAMEA8565066	24.7	339.5	Holstein
SAMEA8565072	24.2	342.5	Holstein
SAMEA8565087	21.5	330.0	Holstein
SAMEA8565088	20.3	261.2	Holstein
SAMEA8565090	19.1	224.9	Holstein
SAMEA6528897	18.1	396.6	Holstein
SAMEA6528902	25.8	342.0	Holstein
SAMEA6528903	20.6	363.9	Holstein
SAMEA7690209	18.9	347.2	Holstein

SAMEA7690211	17.2	344.0	Holstein
SAMEA7690225	18.5	281.0	Holstein
SAMEA7690226	19.3	345.1	Holstein
SAMEA7690227	19.8	315.6	Holstein
SAMEA7690250	18.8	375.9	Holstein
SAMEA7690207	17.5	343.5	Holstein
SAMEA5159880	33.5	450.0	Holstein
SAMEA5159833	23.0	452.8	Holstein
SAMEA7690224	17.5	338.7	Holstein
SAMEA7690233	18.6	305.9	Holstein
SAMEA5159882	29.6	446.9	Holstein
SAMEA5159881	20.8	446.2	Holstein
SAMEA5159883	28.3	441.9	Holstein
SAMN08612501	27.1	454.9	Jersey
SAMN08612533	13.2	509.1	Jersey
SAMEA5159763	17.8	425.5	Limousin
SAMEA7690229	17.5	332.2	Limousin
SAMEA8565043	21.3	333.1	Limousin
SAMEA7690204	17.6	333.2	Limousin
SAMEA7690205	17.8	342.6	Limousin
SAMEA7690206	17.6	343.6	Limousin
SAMEA3390167	15.4	189.9	Montbeliarde
SAMEA3390170	17.6	299.3	Montbeliarde
SAMEA32980918	17.3	407.6	Brown Swiss
SAMEA32981668	15.7	420.4	Brown Swiss
SAMEA32982418	13.5	412.1	Brown Swiss
SAMEA3390189	22.3	233.8	Normande
SAMEA3390191	26.8	249.6	Normande
SAMEA33001168	17.1	401.4	Normande
SAMEA2422242	13.1	351.0	Romagnola
SAMEA5159836	27.7	488.6	Piemontese
SAMEA19324918	14.2	384.1	Piemontese
SAMEA33001918	13.8	388.1	Piemontese
SAMEA5159845	32.8	447.7	Pinzgauer
SAMEA19852168	18.0	244.7	Simmental
SAMEA19852918	37.2	219.2	Simmental
SAMEA19853668	19.6	284.2	Simmental
SAMEA3706829	12.1	400.0	Danish Red Dairy
SAMEA3706828	14.8	387.1	Danish Red Dairy
SAMEA4644767	16.7	429.8	Holstein
SAMEA8565007	20.3	472.4	Holstein
SAMEA8565001	24.7	452.7	Holstein
SAMEA8565015	17.9	458.7	Holstein
SAMEA8565011	20.5	484.4	Holstein
SAMEA8565002	22.6	472.6	Holstein
SAMEA8565012	17.9	471.3	Holstein
SAMEA8565013	18.8	476.3	Holstein
SAMEA8564999	20.7	448.5	Holstein

SAMEA5160021	21.3	464.9	Holstein
SAMEA8565008	18.1	464.9	Holstein
SAMEA8565010	31.4	472.9	Holstein
SAMEA4644726	18.7	402.5	Holstein
SAMEA8564998	41.6	450.4	Holstein
SAMEA8565006	26.9	475.8	Holstein
SAMEA8565003	31.7	466.5	Holstein
SAMEA8565004	20.9	468.5	Holstein
SAMEA19864168	12.7	327.0	Holstein
SAMEA19864918	10.1	315.8	Brown Swiss
SAMEA19865668	16.3	299.3	Holstein
SAMEA19866418	12.4	254.1	Limousin x Holstein
SAMEA19867168	10.1	265.5	Holstein
SAMEA19867918	11.5	300.3	Limousin
SAMEA19868668	15.1	288.7	Simmental
SAMEA19869418	12.5	355.2	Simmental
SAMEA19870168	13.6	351.8	Simmental
SAMEA19871668	12.1	345.9	Holstein
SAMEA3209361	21.4	248.1	Pezzata Rossa Italiana
SAMEA3113485	19.1	375.8	Holstein
SAMEA19874668	18.8	364.2	Holstein
SAMEA32983168	14.8	378.6	Holstein
SAMEA32983918	13.3	396.5	Holstein
SAMEA32984668	18.0	375.3	Holstein
SAMEA33669418	36.0	365.3	Simmental
SAMEA19875418	12.5	372.7	Simmental
SAMEA32985418	14.6	383.9	Limousin
SAMEA19877668	13.8	337.8	Simmental
SAMEA32988418	25.2	416.2	Romagnola
SAMEA32990668	18.1	364.6	Holstein
SAMEA32992918	15.0	363.8	Limousin x Brown Swiss
SAMEA32986918	16.7	404.4	Holstein
SAMEA32991418	13.6	362.3	Holstein
SAMEA32992168	14.2	388.6	Holstein
SAMEA3706814	14.2	351.6	Holstein
SAMEA3706815	14.9	420.4	Holstein
SAMEA3706816	15.1	421.2	Holstein
SAMEA32987668	9.6	455.9	Romagnola
SAMEA32989168	18.9	433.9	Romagnola
SAMEA32995168	22.1	363.7	Holstein
SAMEA32995918	28.2	388.2	Holstein
SAMEA32996668	26.6	414.5	Holstein
SAMEA2821387	13.1	247.9	Charolais
SAMEA2821386	15.5	332.0	Hereford
SAMEA32997418	15.7	409.4	Brown Swiss
SAMEA3706830	15.1	392.2	Holstein
SAMEA3706827	14.2	392.9	Danish Red Dairy
SAMEA8565047	21.5	344.4	Romagnola

SAMEA8565022	18.5	333.0	Romagnola
SAMEA8565021	22.0	327.6	Romagnola
SAMEA6528884	24.5	378.6	Romagnola
SAMEA8565049	21.6	340.2	Romagnola
SAMEA8565020	20.8	328.3	Romagnola
SAMEA6528883	22.7	373.5	Romagnola
SAMEA7690202	17.9	326.0	Romagnola
SAMEA7015114	18.1	381.4	Romagnola
SAMEA8565046	17.8	301.6	Romagnola
SAMEA6528882	20.9	374.4	Romagnola
SAMEA8565017	25.5	332.7	Romagnola
SAMEA8565019	17.0	333.8	Romagnola
SAMEA6528881	21.1	371.8	Romagnola
SAMEA7690203	19.2	326.1	Romagnola
SAMEA8565018	17.7	328.0	Romagnola
SAMEA6528885	22.5	384.0	Romagnola
SAMEA8565024	20.8	294.5	Romagnola
SAMEA8565048	21.8	323.2	Romagnola
SAMEA7690195	17.4	328.5	Romagnola
SAMN10598557	40.1	380.9	Hereford
SAMN10598558	34.6	406.0	Hereford
SAMEA5714970	18.9	378.0	Scotish Highland
SAMEA5159831	17.4	461.1	Scotish Highland
SAMEA5159830	24.2	469.5	Scotish Highland
SAMEA5159829	16.0	457.9	Scotish Highland
SAMEA7690199	20.8	328.9	Simmental
SAMEA5564718	18.6	376.0	Simmental
SAMEA3706826	11.0	416.8	Simmental
SAMEA7690220	19.0	350.1	Simmental
SAMEA5564717	20.3	381.7	Simmental
SAMEA5566449	22.8	380.1	Simmental
SAMEA8565045	21.7	337.8	Simmental
SAMEA8565031	21.5	348.6	Simmental
SAMEA5564719	24.7	376.8	Simmental
SAMEA8565037	23.4	352.6	Simmental
SAMEA5159856	28.7	428.3	Holstein
SAMEA5564720	26.7	386.6	Simmental
SAMEA7690219	17.5	343.2	Simmental
SAMEA5159762	17.2	406.2	Simmental x Holstein
SAMEA5159858	25.2	382.9	Holstein
SAMEA5564722	23.6	362.5	Simmental
SAMEA5564725	23.4	376.4	Simmental
SAMEA7690240	19.0	305.4	Simmental
SAMEA5160153	33.9	458.0	Simmental
SAMEA6528880	17.5	463.5	Simmental
SAMEA4644747	15.4	414.3	Holstein
SAMEA5564715	25.3	444.8	Simmental
SAMEA5564713	19.1	362.2	Simmental

SAMEA5564714	18.2	373.5	Simmental
SAMEA5415499	15.8	371.5	Simmental
SAMEA5159832	35.0	467.9	Holstein
SAMEA5564711	20.8	440.9	Simmental
SAMEA5564710	17.3	437.0	Simmental
SAMEA5564724	22.5	370.2	Simmental
SAMEA8565055	7.6	328.2	Simmental
SAMEA5564723	26.7	371.8	Simmental
SAMEA5564721	21.7	373.4	Simmental
SAMEA7015109	18.9	385.4	Simmental
SAMEA6528910	21.4	383.7	Simmental
SAMEA8565044	22.0	341.0	Simmental
SAMEA4644744	22.1	406.4	Simmental
SAMEA5564712	22.2	484.0	Simmental
SAMEA4644745	18.7	374.7	Simmental
SAMEA5159876	28.2	450.1	Simmental
SAMEA5159877	30.3	449.3	Simmental
SAMEA5415487	35.0	355.4	Simmental
SAMEA33004918	21.4	410.2	Simmental
SAMEA19309168	14.7	416.1	Simmental
SAMEA19876168	11.2	444.1	Romagnola
SAMEA19876918	13.7	415.2	Holstein
SAMEA3682652	18.3	375.9	Tyrolean Grey
SAMEA5159889	24.1	424.7	Tyrolean Grey
SAMEA7015117	18.1	387.3	Grey cattle
SAMEA5716181	17.4	427.4	Tyrolean Grey
SAMEA5564737	22.6	358.0	Tyrolean Grey
SAMEA7015121	15.6	380.9	Grey cattle
SAMEA5159888	24.9	425.7	Tyrolean Grey
SAMEA7015116	18.8	383.1	Grey cattle
SAMEA5564730	25.5	447.8	Tyrolean Grey
SAMEA5714980	18.8	429.1	Tyrolean Grey
SAMEA5564731	19.4	418.2	Tyrolean Grey
SAMEA5564729	18.8	453.8	Tyrolean Grey
SAMEA5564735	15.8	370.0	Tyrolean Grey
SAMEA5159887	25.3	419.3	Tyrolean Grey
SAMEA5564734	24.4	369.3	Tyrolean Grey
SAMEA7015118	16.4	371.6	Grey cattle
SAMEA7015119	25.2	392.8	Grey cattle
SAMEA7015120	19.2	384.8	Grey cattle
SAMEA5564732	23.4	452.1	Tyrolean Grey
SAMEA5564733	18.9	435.2	Tyrolean Grey
SAMEA5714981	17.4	430.7	Tyrolean Grey
SAMEA5564736	18.9	434.1	Tyrolean Grey
SAMEA2357050	12.7	279.2	Tyrolean Grey
SAMEA7589755	23.4	389.6	Grey cattle
SAMEA7589756	21.3	436.0	Grey cattle
SAMEA7589757	24.5	421.6	Grey cattle

SAMEA7589758	26.1	427.9	Grey cattle
SAMEA7589759	24.6	422.6	Grey cattle
SAMEA7589760	26.6	472.4	Grey cattle
SAMEA7589761	20.0	431.3	Grey cattle
SAMEA7589762	27.4	398.7	Grey cattle
SAMEA7589763	24.3	426.5	Grey cattle
SAMEA5159846	43.8	443.2	Tux-Zillertal
SAMN08473804	48.7	426.1	Angus
SAMN09379712	25.7	550.4	Charolais
SAMN09510423	39.0	497.9	Norwegian Red
SAMEA8565083	21.4	339.5	unknown
SAMEA8565054	20.3	343.1	Holstein x unknown
SAMEA6528901	8.9	392.9	Danish Red Dairy
SAMEA5415493	21.2	374.2	Belgium Blue x Holstein
SAMEA5415495	24.2	357.8	Belgium Blue x Holstein
SAMEA5415497	21.5	366.6	Belgium Blue x Holstein
SAMEA5714973	17.6	448.5	Angus x Simmental
SAMEA3706824	13.0	410.1	Simmental x Angus
SAMEA7690218	19.0	343.0	Limousin x Simmental
SAMEA19872418	28.5	384.5	Piemontese x Normande
SAMEA7690221	17.6	346.3	Limousin x Simmental
SAMEA5159851	25.7	461.1	Australian Lowline
SAMEA6528898	19.0	388.5	Belgian Blue x Holstein
SAMEA6528900	22.4	379.9	Danish Red Dairy x Holstein

Supplementary Table S3: Comprehensive list including all haplotypes of interest and detailed information.

name[a]	analysis	chr	start[b]	end[b]	N SNPs	N unique haplotypes	N genotyped animals	observed number of homozygous carrier	expected number of homozygous carrier	N heterozygous observed (expeccted)
**HH18**	pgp	1	103019622	104375908	50	1989	52423	7	30	2488 (2442)
**HH19**[c]	pgp	1	139873827	140642688	50	2078	52547	12	47	3133 (3062)
**HH20**	pgp	2	134476837	135147756	50	4246	52071	1	14	1728 (1701)
HH21 (175.5 and 07.126) [d]	trio	7	7871925	10432630	50	3972	52577	0	157	5746 (5432)
	pgp	7	7871925	10432630	50	3972	52577	0	157	5746 (5432)
**HH22**	pgp	7	93581943	94642456	50	2546	52550	2	16	1812 (1785)
**HH23**	pgp	8	15561949	16862751	50	4124	52569	5	25	2298 (2257)
**HH24**	pgp	8	66699044	68005926	50	2449	52693	2	59	3533 (3418)
HH3 [e]	trio	8	90958661	92084831	50	1735	52662	0	10	1455 (1435)
	pgp	8	90958661	92084831	50	1735	52662	0	10	1455 (1435)
HH5 [f]	trio	9	90928417	91823561	50	2696	52304	0	30	2505 (2445)
	pgp	9	90928417	91823561	50	2696	52304	0	30	2505 (2445)
**HH25**	trio	10	86876435	87772797	50	2378	52623	5	20	2048 (2018)
**HH26**	pgp	11	4531428	5362251	50	3346	52669	7	34	2678 (2623)
**HH27**	pgp	13	7083081	8296886	50	2585	52336	2	17	1906 (1875)
**HH28**	pgp	14	24591331	24827448	50	735	52505	3	20	2068 (2033)
**HH29**	pgp	14	58128474	59238055	50	2844	52376	14	52	3259 (3184)
HH13 [g]	trio	18	60931980	62100899	50	2766	52778	1	17	1911 (1878)
	pgp	18	62070448	63044863	50	4891	52636	2	22	2162 (2121)
**HH30**	trio	21	7844296	8671178	50	2375	52720	1	24	2246 (2200)
**HH31**	pgp	22	59811442	60703550	50	3372	52208	3	17	1875 (1847)

**HH32**	pgp	23	32773236	33726454	50	3627	52393	1	13	1638 (1614)
	trio	23	33544569	34948131	50	4519	52402	0	13	1623 (1598)
**HH33**	pgp	24	44692769	45677988	50	2517	52496	14	42	2933 (2877)
**HH34**	pgp	25	36165207	37329782	50	3222	52619	0	18	1959 (1923)
	trio	25	36165207	37329782	50	3222	52619	0	18	1959 (1923)
**HH35**	pgp	26	3358717	4234871	50	2647	52604	7	31	2552 (2503)
**HH36**	pgp	28	28541274	29736156	50	3054	52358	2	15	1784 (1757)
	trio	28	29731696	30877496	50	2572	52345	2	26	2348 (2299)
**HH37**	pgp	28	40347735	41271169	50	3087	52508	6	66	3705 (3585)
**HH38**	trio	29	14242732	15316961	50	3290	52564	0	14	1740 (1711)

[a] HH meaning Holstein Haplotype

[b] according to the reference sequence ARS-UCD1.2 (National Center for Biotechnology Information, 2018a)

[c] haplotype co-localises with previously described haplotype HHB by Shuster *et al. (* 1992) and Cole *et al.* (2018)

[d] haplotype previously described by VanRaden *et al.* (2011) and Sahana *et al.* (2013)

[e] haplotype previously described by VanRaden *et al.* (2011), McClure *et al.* (2013), Sahana *et al.* (2013) and Wu *et al.* (2019)

[f] haplotype previously described by Schütz *et al.* (2016) and Fritz *et al.* (2018)

[g] haplotype previously described by Fritz *et al.* (2013)

allele frequency %	Haplotype	p-value	p-value adjusted to BY	length Mb
2.39	H=121221212111112121222112112211221211121212111121112	7.52E-07	1.78E-04	1.36
3.00	H=221111222212112212112212211221121221112111121212112	7.72E-10	4.08E-07	0.77
1.66	H=221211111222112211211121212211211211111212111122211	1.18E-05	1.98E-03	0.67
5.46	H=222122122121222222112122221221212122211122212222112	1.10E-72	9.96E-70	2.56
5.46	H=222122122121222222112122221221212122211122212222112	1.10E-72	2.51E-69	2.56
1.73	H=211222211112222222112122122211122122212211111112111	2.93E-05	4.03E-03	1.06
2.20	H=221211122211211222212212112122222222221111112211211	1.28E-06	2.82E-04	1.30
3.36	H=122222212212212212121121221111212211121211222122222	7.19E-24	1.04E-20	1.31
1.38	H=112211121121121221121111111221122122221211212211212	7.47E-05	2.10E-03	1.13
1.38	H=112211121121121221121111111221122122221211212211212	7.47E-05	7.84E-03	1.13
2.39	H=111111121222121121212122221222122222222222221221211	7.66E-14	2.86E-11	0.90
2.39	H=111111121222121121212122221222122222222222221221211	7.66E-14	5.84E-11	0.90
1.96	H=121211212212211112112122211222112112212122221221121	9.26E-05	2.44E-03	0.90
2.56	H=211112121211111122112221212122222111111111121112222	2.03E-08	6.87E-06	0.83
1.82	H=122111212112221222211111221122211221221222121211111	7.15E-06	1.28E-03	1.21
1.98	H=221212121122112222211222122212211212121222211112122	2.64E-06	5.53E-04	0.24
3.14	H=122212222111212122122212121222222222122222111212121	5.70E-10	3.05E-07	1.11
1.81	H=121211111112222121121121211211122212112121221121122	9.18E-07	5.61E-05	1.17
2.06	H=211111212122211112221111212121121221112211212222122	9.80E-08	2.96E-05	0.97
2.13	H=111222111121221212211211222222222211122212222121111	9.78E-10	1.99E-07	0.83
1.80	H=2221111212212111221212222122112122221222211122221212	5.96E-05	6.84E-03	0.89

1.57	H=11111212112112211111222112212222111121112212211222	5.20E-05	6.18E-03	0.95
1.55	H=12211222222212222212122212221112112112222121121212	4.84E-06	2.23E-04	1.40
2.82	H=22112221121221221221211112222111222121121211112112	6.29E-07	1.54E-04	0.99
1.86	H=11112221112221112121121222222112111212112122212222	1.43E-08	5.32E-06	1.16
1.86	H=11112221112221112121121222222112111212112122212222	1.43E-08	1.88E-06	1.16
2.44	H=12112211112221111112111122212222222222122122212122	2.01E-07	5.19E-05	0.88
1.71	H=12122112211122122121211222121212211122212121221212	6.41E-05	7.12E-03	1.19
2.25	H=12222122111121211121221212121122212112211122112122	1.61E-09	2.90E-07	1.15
3.54	H=21222221211211221221111221111221212112222112221121	8.79E-22	1.03E-18	0.92
1.66	H=21212211121111212121212221221121211211111112222112	6.95E-07	4.66E-05	1.07

Supplementary Table S4: Results of the haplotype effect estimation with fertility, reproduction and survival traits for the Swiss Holstein population.

name	haplotype	trait	trait description	freq	b-value	se	p-value
HH18	1-103019622-104375908	P2b	survival bull period 2	0.0100	-0.978	3.496	0.780
	1-103019622-104375908	BWd	birth weight	0.0088	-0.866	1.409	0.539
	1-103019622-104375908	BWm	birth weight	0.0085	-0.407	1.568	0.795
	1-103019622-104375908	P2h	survival heifer period 2	0.0102	-5.874	6.006	0.328
	1-103019622-104375908	SBd	percentage live births	0.0069	1.922	2.822	0.496
	1-103019622-104375908	SBm	percentage live births	0.0033	-4.211	2.374	0.076
	1-103019622-104375908	CEd	percentage normal births	0.0021	0.404	0.897	0.652
	1-103019622-104375908	CEm	percentage normal births	0.0027	1.704	1.417	0.229
	1-103019622-104375908	NRc	non-return rate cow	0.0018	0.528	1.537	0.731
	1-103019622-104375908	NRh	non-return rate heifer	0.0019	-0.564	2.123	0.791
	1-103019622-104375908	P1	survival period 1	0.0077	3.708	4.059	0.361
	1-103019622-104375908	DFS	interval calving to insemination	0.0015	1.774	1.498	0.236
	1-103019622-104375908	GLd	gestation length	0.0102	0.047	1.327	0.972
	1-103019622-104375908	GLm	gestation length	0.0094	1.225	1.575	0.437
	1-103019622-104375908	MBd	multiple birth	0.0096	1.199	1.865	0.520
	1-103019622-104375908	MBm	multiple birth	0.0080	3.233	2.354	0.170
	1-103019622-104375908	IFLc	interval first to last insemination cow	0.0014	2.394	1.413	0.090
	1-103019622-104375908	IFLh	interval first to last insemination heifer	0.0049	0.171	2.548	0.946
HH19	1-139873827-140642688	P2b	survival bull period 2	0.0100	-1.003	3.555	0.778
	1-139873827-140642688	BWd	birth weight	0.0093	1.407	1.345	0.296
	1-139873827-140642688	BWm	birth weight	0.0085	0.048	1.530	0.975
	1-139873827-140642688	P2h	survival heifer period 2	0.0102	-2.722	5.959	0.648
	1-139873827-140642688	SBd	percentage live births	0.0086	2.783	2.494	0.264
	1-139873827-140642688	SBm	percentage live births	0.0039	-1.708	2.162	0.429
	1-139873827-140642688	CEd	percentage normal births	0.0026	-0.859	0.802	0.284
	1-139873827-140642688	CEm	percentage normal births	0.0034	1.622	1.260	0.198
	1-139873827-140642688	NRc	non-return rate cow	0.0020	0.321	1.417	0.821
	1-139873827-140642688	NRh	non-return rate heifer	0.0023	1.495	1.909	0.434
	1-139873827-140642688	P1	survival period 1	0.0073	-5.187	4.170	0.214
	1-139873827-140642688	DFS	interval calving to insemination	0.0019	0.671	1.331	0.614
	1-139873827-140642688	GLd	gestation length	0.0113	2.599	1.258	**0.039**
	1-139873827-140642688	GLm	gestation length	0.0111	2.122	1.454	0.144
	1-139873827-140642688	MBd	multiple birth	0.0076	-2.237	2.128	0.293
	1-139873827-140642688	MBm	multiple birth	0.0063	0.552	2.620	0.833
	1-139873827-140642688	IFLc	interval first to last insemination cow	0.0018	0.408	1.256	0.745

	1-139873827-140642688	IFLh	interval first to last insemination heifer	0.0048	-0.205	2.568	0.936
HH25	10-86876435-87772797	P2b	survival bull period 2	0.0154	-2.983	3.252	0.359
	10-86876435-87772797	BWd	birth weight	0.0140	-0.867	1.321	0.511
	10-86876435-87772797	BWm	birth weight	0.0133	-0.226	1.494	0.880
	10-86876435-87772797	P2h	survival heifer period 2	0.0152	0.934	5.673	0.869
	10-86876435-87772797	SBd	percentage live births	0.0157	-2.126	2.209	0.336
	10-86876435-87772797	SBm	percentage live births	0.0177	-1.326	1.399	0.344
	10-86876435-87772797	CEd	percentage normal births	0.0144	0.046	0.482	0.925
	10-86876435-87772797	CEm	percentage normal births	0.0165	0.319	0.791	0.687
	10-86876435-87772797	NRc	non-return rate cow	0.0169	-1.196	0.698	0.087
	10-86876435-87772797	NRh	non-return rate heifer	0.0155	0.120	0.953	0.900
	10-86876435-87772797	P1	survival period 1	0.0158	-2.508	3.241	0.439
	10-86876435-87772797	DFS	interval calving to insemination	0.0156	1.637	0.668	**0.014**
	10-86876435-87772797	GLd	gestation length	0.0137	0.536	1.335	0.688
	10-86876435-87772797	GLm	gestation length	0.0138	1.887	1.533	0.218
	10-86876435-87772797	MBd	multiple birth	0.0166	-1.937	1.576	0.219
	10-86876435-87772797	MBm	multiple birth	0.0102	4.202	2.402	0.080
	10-86876435-87772797	IFLc	interval first to last insemination cow	0.0152	0.046	0.632	0.942
	10-86876435-87772797	IFLh	interval first to last insemination heifer	0.0093	-2.074	2.137	0.332
HH26	11-4531428-5362251	P2b	survival bull period 2	0.0063	-1.728	4.418	0.696
	11-4531428-5362251	BWd	birth weight	0.0058	0.961	1.695	0.570
	11-4531428-5362251	BWm	birth weight	0.0059	-1.635	1.844	0.375
	11-4531428-5362251	P2h	survival heifer period 2	0.0074	-3.060	7.055	0.664
	11-4531428-5362251	SBd	percentage live births	0.0059	1.207	2.976	0.685
	11-4531428-5362251	SBm	percentage live births	0.0040	-0.153	2.142	0.943
	11-4531428-5362251	CEd	percentage normal births	0.0029	-0.449	0.753	0.551
	11-4531428-5362251	CEm	percentage normal births	0.0034	1.601	1.255	0.202
	11-4531428-5362251	NRc	non-return rate cow	0.0030	0.618	1.210	0.610
	11-4531428-5362251	NRh	non-return rate heifer	0.0032	-0.598	1.675	0.721
	11-4531428-5362251	P1	survival period 1	0.0056	0.534	4.736	0.910
	11-4531428-5362251	DFS	interval calving to insemination	0.0025	0.662	1.177	0.574
	11-4531428-5362251	GLd	gestation length	0.0067	5.004	1.592	**0.002**
	11-4531428-5362251	GLm	gestation length	0.0062	3.484	1.913	0.069
	11-4531428-5362251	MBd	multiple birth	0.0057	-0.911	2.488	0.714
	11-4531428-5362251	MBm	multiple birth	0.0067	0.902	2.610	0.730
	11-4531428-5362251	IFLc	interval first to last insemination cow	0.0025	0.369	1.086	0.734
	11-4531428-5362251	IFLh	interval first to last insemination heifer	0.0042	0.058	2.803	0.983
	13-7083081-8296886	P2b	survival bull period 2	0.0068	-0.271	4.292	0.950

**HH7**	13-7083081-8296886	BWd	birth weight	0.0083	1.828	1.462	0.211
	13-7083081-8296886	BWm	birth weight	0.0077	-0.165	1.639	0.920
	13-7083081-8296886	P2h	survival heifer period 2	0.0074	-7.479	7.069	0.290
	13-7083081-8296886	SBd	percentage live births	0.0074	-0.954	2.713	0.725
	13-7083081-8296886	SBm	percentage live births	0.0054	-1.806	1.860	0.332
	13-7083081-8296886	CEd	percentage normal births	0.0045	-0.884	0.621	0.155
	13-7083081-8296886	CEm	percentage normal births	0.0052	-0.224	1.037	0.829
	13-7083081-8296886	NRc	non-return rate cow	0.0047	-0.896	0.972	0.356
	13-7083081-8296886	NRh	non-return rate heifer	0.0041	-1.238	1.476	0.402
	13-7083081-8296886	P1	survival period 1	0.0064	5.684	4.479	0.204
	13-7083081-8296886	DFS	interval calving to insemination	0.0042	-0.553	0.911	0.544
	13-7083081-8296886	GLd	gestation length	0.0088	0.241	1.425	0.866
	13-7083081-8296886	GLm	gestation length	0.0080	3.815	1.717	**0.026**
	13-7083081-8296886	MBd	multiple birth	0.0059	2.537	2.425	0.295
	13-7083081-8296886	MBm	multiple birth	0.0056	0.042	2.816	0.988
	13-7083081-8296886	IFLc	interval first to last insemination cow	0.0042	-0.291	0.852	0.733
	13-7083081-8296886	IFLh	interval first to last insemination heifer	0.0044	-2.066	2.742	0.451
**HH28**	14-24591331-24827448	P2b	survival bull period 2	0.0098	-1.327	3.528	0.707
	14-24591331-24827448	BWd	birth weight	0.0086	-0.301	1.414	0.831
	14-24591331-24827448	BWm	birth weight	0.0085	2.719	1.547	0.079
	14-24591331-24827448	P2h	survival heifer period 2	0.0116	0.737	5.555	0.894
	14-24591331-24827448	SBd	percentage live births	0.0088	5.122	2.479	**0.039**
	14-24591331-24827448	SBm	percentage live births	0.0060	3.964	1.758	**0.024**
	14-24591331-24827448	CEd	percentage normal births	0.0049	-0.007	0.601	0.990
	14-24591331-24827448	CEm	percentage normal births	0.0054	1.088	1.017	0.285
	14-24591331-24827448	NRc	non-return rate cow	0.0052	-0.708	0.921	0.442
	14-24591331-24827448	NRh	non-return rate heifer	0.0055	-0.080	1.274	0.950
	14-24591331-24827448	P1	survival period 1	0.0098	-2.435	3.570	0.495
	14-24591331-24827448	DFS	interval calving to insemination	0.0049	0.383	0.866	0.658
	14-24591331-24827448	GLd	gestation length	0.0086	0.413	1.414	0.770
	14-24591331-24827448	GLm	gestation length	0.0086	0.865	1.620	0.593
	14-24591331-24827448	MBd	multiple birth	0.0103	-0.373	1.827	0.838
	14-24591331-24827448	MBm	multiple birth	0.0067	-2.869	2.524	0.256
	14-24591331-24827448	IFLc	interval first to last insemination cow	0.0046	-0.205	0.821	0.803
	14-24591331-24827448	IFLh	interval first to last insemination heifer	0.0070	-1.805	2.131	0.397
	14-58128474-59238055	P2b	survival bull period 2	0.0096	-0.319	3.619	0.930
	14-58128474-59238055	BWd	birth weight	0.0087	0.016	1.407	0.991
	14-58128474-59238055	BWm	birth weight	0.0071	-1.691	1.693	0.318
	14-58128474-59238055	P2h	survival heifer period 2	0.0083	10.125	6.601	0.125

HH29	14-58128474-59238055	SBd	percentage live births	0.0076	1.238	2.646	0.640
	14-58128474-59238055	SBm	percentage live births	0.0031	5.298	2.402	**0.027**
	14-58128474-59238055	CEd	percentage normal births	0.0031	0.728	0.734	0.321
	14-58128474-59238055	CEm	percentage normal births	0.0031	2.625	1.315	**0.046**
	14-58128474-59238055	NRc	non-return rate cow	0.0020	0.384	1.456	0.792
	14-58128474-59238055	NRh	non-return rate heifer	0.0025	-2.481	1.859	0.182
	14-58128474-59238055	P1	survival period 1	0.0081	1.991	3.958	0.615
	14-58128474-59238055	DFS	interval calving to insemination	0.0018	2.029	1.376	0.140
	14-58128474-59238055	GLd	gestation length	0.0087	0.825	1.421	0.562
	14-58128474-59238055	GLm	gestation length	0.0088	0.435	1.612	0.787
	14-58128474-59238055	MBd	multiple birth	0.0057	-2.184	2.438	0.370
	14-58128474-59238055	MBm	multiple birth	0.0050	-0.578	2.918	0.843
	14-58128474-59238055	IFLc	interval first to last insemination cow	0.0017	0.304	1.306	0.816
	14-58128474-59238055	IFLh	interval first to last insemination heifer	0.0037	-2.092	2.916	0.473
HH13	18-60931980-62100899	P2b	survival bull period 2	0.0063	-3.189	4.745	0.502
	18-60931980-62100899	BWd	birth weight	0.0077	4.769	1.654	**0.004**
	18-60931980-62100899	BWm	birth weight	0.0075	2.237	1.826	0.221
	18-60931980-62100899	P2h	survival heifer period 2	0.0051	5.964	9.002	0.508
	18-60931980-62100899	SBd	percentage live births	0.0137	-4.283	2.337	0.067
	18-60931980-62100899	SBm	percentage live births	0.0105	0.953	1.566	0.543
	18-60931980-62100899	CEd	percentage normal births	0.0099	-0.673	0.502	0.180
	18-60931980-62100899	CEm	percentage normal births	0.0103	0.557	0.865	0.519
	18-60931980-62100899	NRc	non-return rate cow	0.0089	0.086	0.812	0.916
	18-60931980-62100899	NRh	non-return rate heifer	0.0102	0.190	1.073	0.859
	18-60931980-62100899	P1	survival period 1	0.0079	8.882	4.323	**0.040**
	18-60931980-62100899	DFS	interval calving to insemination	0.0087	0.903	0.755	0.232
	18-60931980-62100899	GLd	gestation length	0.0083	2.504	1.627	0.124
	18-60931980-62100899	GLm	gestation length	0.0074	-0.989	1.925	0.608
	18-60931980-62100899	MBd	multiple birth	0.0050	0.741	2.715	0.785
	18-60931980-62100899	MBm	multiple birth	0.0013	-4.618	5.819	0.427
	18-60931980-62100899	IFLc	interval first to last insemination cow	0.0085	-0.164	0.712	0.818
	18-60931980-62100899	IFLh	interval first to last insemination heifer	0.0018	-4.983	4.358	0.253
	18-62070448-63044863	P2b	survival bull period 2	0.0047	6.285	5.096	0.218
	18-62070448-63044863	BWd	birth weight	0.0078	-0.601	1.506	0.690
	18-62070448-63044863	BWm	birth weight	0.0057	-1.050	1.896	0.580
	18-62070448-63044863	P2h	survival heifer period 2	0.0037	6.804	9.557	0.477
	18-62070448-63044863	SBd	percentage live births	0.0061	-3.248	3.003	0.279
	18-62070448-63044863	SBm	percentage live births	0.0027	-3.870	2.610	0.138
	18-62070448-63044863	CEd	percentage normal births	0.0023	1.014	0.858	0.237

**HH13**	18-62070448-63044863	CEm	percentage normal births	0.0026	-0.155	1.442	0.915
	18-62070448-63044863	NRc	non-return rate cow	0.0015	-1.857	1.636	0.256
	18-62070448-63044863	NRh	non-return rate heifer	0.0016	-2.398	2.337	0.305
	18-62070448-63044863	P1	survival period 1	0.0027	-0.754	6.601	0.909
	18-62070448-63044863	DFS	interval calving to insemination	0.0014	0.006	1.514	0.997
	18-62070448-63044863	GLd	gestation length	0.0077	0.083	1.518	0.956
	18-62070448-63044863	GLm	gestation length	0.0074	1.084	1.769	0.540
	18-62070448-63044863	MBd	multiple birth	0.0037	-0.936	2.995	0.755
	18-62070448-63044863	MBm	multiple birth	0.0035	1.080	3.453	0.754
	18-62070448-63044863	IFLc	interval first to last insemination cow	0.0016	0.141	1.338	0.916
	18-62070448-63044863	IFLh	interval first to last insemination heifer	0.0016	-4.736	4.219	0.262
**HH20**	2-134476837-135147756	P2b	survival bull period 2	0.0039	3.999	5.558	0.472
	2-134476837-135147756	BWd	birth weight	0.0042	-1.253	1.984	0.528
	2-134476837-135147756	BWm	birth weight	0.0035	-3.120	2.361	0.186
	2-134476837-135147756	P2h	survival heifer period 2	0.0037	1.285	9.639	0.894
	2-134476837-135147756	SBd	percentage live births	0.0029	1.859	4.160	0.655
	2-134476837-135147756	SBm	percentage live births	0.0012	6.560	3.697	0.076
	2-134476837-135147756	CEd	percentage normal births	0.0012	-1.051	1.148	0.360
	2-134476837-135147756	CEm	percentage normal births	0.0012	3.763	2.042	0.065
	2-134476837-135147756	NRc	non-return rate cow	0.0008	0.507	2.233	0.820
	2-134476837-135147756	NRh	non-return rate heifer	0.0009	-0.438	2.979	0.883
	2-134476837-135147756	P1	survival period 1	0.0037	-3.545	5.767	0.539
	2-134476837-135147756	DFS	interval calving to insemination	0.0007	2.229	2.126	0.294
	2-134476837-135147756	GLd	gestation length	0.0044	1.535	1.949	0.431
	2-134476837-135147756	GLm	gestation length	0.0041	0.475	2.306	0.837
	2-134476837-135147756	MBd	multiple birth	0.0031	-0.663	3.315	0.841
	2-134476837-135147756	MBm	multiple birth	0.0024	5.183	4.180	0.215
	2-134476837-135147756	IFLc	interval first to last insemination cow	0.0007	0.965	1.979	0.626
	2-134476837-135147756	IFLh	interval first to last insemination heifer	0.0018	1.637	4.058	0.687
**30**	21-7844296-8671178	P2b	survival bull period 2	0.0040	4.689	5.496	0.394
	21-7844296-8671178	BWd	birth weight	0.0044	-4.072	1.957	**0.037**
	21-7844296-8671178	BWm	birth weight	0.0041	-2.270	2.207	0.304
	21-7844296-8671178	P2h	survival heifer period 2	0.0028	12.672	#####	0.255
	21-7844296-8671178	SBd	percentage live births	0.0035	6.314	3.868	0.103
	21-7844296-8671178	SBm	percentage live births	0.0012	-4.643	3.746	0.215
	21-7844296-8671178	CEd	percentage normal births	0.0012	2.811	1.168	**0.016**
	21-7844296-8671178	CEm	percentage normal births	0.0013	4.793	2.003	**0.017**
	21-7844296-8671178	NRc	non-return rate cow	0.0007	-1.182	2.449	0.629
	21-7844296-8671178	NRh	non-return rate heifer	0.0007	3.742	3.396	0.271

HH[illegible]	21-7844296-8671178	P1	survival period 1	0.0029	0.922	6.526	0.888
	21-7844296-8671178	DFS	interval calving to insemination	0.0005	1.193	2.517	0.635
	21-7844296-8671178	GLd	gestation length	0.0049	-0.110	1.896	0.954
	21-7844296-8671178	GLm	gestation length	0.0046	-1.649	2.235	0.461
	21-7844296-8671178	MBd	multiple birth	0.0024	4.538	3.723	0.223
	21-7844296-8671178	MBm	multiple birth	0.0019	0.300	4.655	0.949
	21-7844296-8671178	IFLc	interval first to last insemination cow	0.0005	1.898	2.291	0.407
	21-7844296-8671178	IFLh	interval first to last insemination heifer	0.0009	2.002	5.669	0.724
HH30	22-59811442-60703550	P2b	survival bull period 2	0.0047	2.523	5.041	0.617
	22-59811442-60703550	BWd	birth weight	0.0052	-0.157	1.774	0.929
	22-59811442-60703550	BWm	birth weight	0.0055	-2.056	1.891	0.277
	22-59811442-60703550	P2h	survival heifer period 2	0.0037	-8.642	9.650	0.370
	22-59811442-60703550	SBd	percentage live births	0.0045	4.786	3.400	0.159
	22-59811442-60703550	SBm	percentage live births	0.0021	0.733	2.902	0.800
	22-59811442-60703550	CEd	percentage normal births	0.0014	0.140	1.070	0.896
	22-59811442-60703550	CEm	percentage normal births	0.0017	2.992	1.745	0.086
	22-59811442-60703550	NRc	non-return rate cow	0.0009	-0.534	2.112	0.801
	22-59811442-60703550	NRh	non-return rate heifer	0.0010	-2.848	2.785	0.307
	22-59811442-60703550	P1	survival period 1	0.0042	2.880	5.434	0.596
	22-59811442-60703550	DFS	interval calving to insemination	0.0009	2.138	1.892	0.259
	22-59811442-60703550	GLd	gestation length	0.0061	3.284	1.672	**0.050**
	22-59811442-60703550	GLm	gestation length	0.0058	4.356	1.961	**0.026**
	22-59811442-60703550	MBd	multiple birth	0.0048	4.492	2.655	0.091
	22-59811442-60703550	MBm	multiple birth	0.0024	-5.100	4.171	0.221
	22-59811442-60703550	IFLc	interval first to last insemination cow	0.0008	0.430	1.805	0.811
	22-59811442-60703550	IFLh	interval first to last insemination heifer	0.0022	4.432	3.742	0.236
HH32	23-32773236-33726454	P2b	survival bull period 2	0.0000	-2.464	4.744	0.603
	23-32773236-33726454	BWd	birth weight	0.0000	-3.708	1.804	**0.040**
	23-32773236-33726454	BWm	birth weight	0.0000	-1.444	2.024	0.476
	23-32773236-33726454	P2h	survival heifer period 2	0.0000	-3.161	9.011	0.726
	23-32773236-33726454	SBd	percentage live births	0.0000	4.422	3.686	0.230
	23-32773236-33726454	SBm	percentage live births	0.0000	2.775	3.306	0.401
	23-32773236-33726454	CEd	percentage normal births	0.0000	0.857	1.204	0.476
	23-32773236-33726454	CEm	percentage normal births	0.0000	4.495	1.873	**0.016**
	23-32773236-33726454	NRc	non-return rate cow	0.0000	2.901	2.229	0.193
	23-32773236-33726454	NRh	non-return rate heifer	0.0000	0.905	3.028	0.765
	23-32773236-33726454	P1	survival period 1	0.0000	4.209	5.259	0.423
	23-32773236-33726454	DFS	interval calving to insemination	0.0000	1.944	2.186	0.374

	23-32773236-33726454	GLd	gestation length	0.0000	0.851	1.699	0.617
	23-32773236-33726454	GLm	gestation length	0.0000	0.544	2.019	0.787
	23-32773236-33726454	MBd	multiple birth	0.0000	1.850	2.725	0.497
	23-32773236-33726454	MBm	multiple birth	0.0000	-4.466	4.132	0.280
	23-32773236-33726454	IFLc	interval first to last insemination cow	0.0000	3.321	1.954	0.089
	23-32773236-33726454	IFLh	interval first to last insemination heifer	0.0000	0.023	3.293	0.995
**HH32**	23-33544569-34948131	P2b	survival bull period 2	0.0000	-1.652	4.704	0.725
	23-33544569-34948131	BWd	birth weight	0.0000	-2.656	1.767	0.133
	23-33544569-34948131	BWm	birth weight	0.0000	-0.388	1.971	0.844
	23-33544569-34948131	P2h	survival heifer period 2	0.0000	5.614	8.713	0.519
	23-33544569-34948131	SBd	percentage live births	0.0000	2.499	3.595	0.487
	23-33544569-34948131	SBm	percentage live births	0.0000	4.826	3.182	0.129
	23-33544569-34948131	CEd	percentage normal births	0.0000	0.995	1.188	0.402
	23-33544569-34948131	CEm	percentage normal births	0.0000	4.835	1.807	**0.007**
	23-33544569-34948131	NRc	non-return rate cow	0.0000	3.087	2.124	0.146
	23-33544569-34948131	NRh	non-return rate heifer	0.0000	0.817	2.908	0.779
	23-33544569-34948131	P1	survival period 1	0.0000	7.390	5.205	0.156
	23-33544569-34948131	DFS	interval calving to insemination	0.0000	3.064	2.120	0.148
	23-33544569-34948131	GLd	gestation length	0.0000	3.460	1.639	**0.035**
	23-33544569-34948131	GLm	gestation length	0.0000	3.326	1.995	0.095
	23-33544569-34948131	MBd	multiple birth	0.0000	3.735	2.619	0.154
	23-33544569-34948131	MBm	multiple birth	0.0000	4.091	4.006	0.307
	23-33544569-34948131	IFLc	interval first to last insemination cow	0.0000	3.750	1.900	**0.048**
	23-33544569-34948131	IFLh	interval first to last insemination heifer	0.0000	-0.524	3.341	0.875
**HH33**	24-44692769-45677988	P2b	survival bull period 2	0.0112	-0.031	3.345	0.993
	24-44692769-45677988	BWd	birth weight	0.0113	0.918	1.246	0.461
	24-44692769-45677988	BWm	birth weight	0.0098	1.953	1.446	0.177
	24-44692769-45677988	P2h	survival heifer period 2	0.0143	4.129	5.087	0.417
	24-44692769-45677988	SBd	percentage live births	0.0077	6.026	2.624	**0.022**
	24-44692769-45677988	SBm	percentage live births	0.0048	0.733	2.011	0.716
	24-44692769-45677988	CEd	percentage normal births	0.0034	-0.337	0.718	0.639
	24-44692769-45677988	CEm	percentage normal births	0.0040	1.528	1.189	0.198
	24-44692769-45677988	NRc	non-return rate cow	0.0034	-0.650	1.156	0.574
	24-44692769-45677988	NRh	non-return rate heifer	0.0039	-1.053	1.560	0.500
	24-44692769-45677988	P1	survival period 1	0.0104	-0.394	3.510	0.911
	24-44692769-45677988	DFS	interval calving to insemination	0.0029	1.954	1.126	0.083
	24-44692769-45677988	GLd	gestation length	0.0115	1.981	1.245	0.112
	24-44692769-45677988	GLm	gestation length	0.0112	1.630	1.449	0.261
	24-44692769-45677988	MBd	multiple birth	0.0120	0.366	1.700	0.829

	24-44692769-45677988	MBm	multiple birth	0.0104	-0.312	2.040	0.879
	24-44692769-45677988	IFLc	interval first to last insemination cow	0.0028	0.347	1.049	0.741
	24-44692769-45677988	IFLh	interval first to last insemination heifer	0.0070	-0.802	2.149	0.709
**HH34**	25-36165207-37329782	P2b	survival bull period 2	0.0110	-2.621	3.461	0.449
	25-36165207-37329782	BWd	birth weight	0.0124	-0.383	1.289	0.766
	25-36165207-37329782	BWm	birth weight	0.0115	-0.283	1.424	0.843
	25-36165207-37329782	P2h	survival heifer period 2	0.0125	-5.333	5.597	0.341
	25-36165207-37329782	SBd	percentage live births	0.0132	0.844	2.173	0.698
	25-36165207-37329782	SBm	percentage live births	0.0103	0.717	1.397	0.608
	25-36165207-37329782	CEd	percentage normal births	0.0097	0.351	0.440	0.424
	25-36165207-37329782	CEm	percentage normal births	0.0107	0.669	0.753	0.374
	25-36165207-37329782	NRc	non-return rate cow	0.0095	-0.586	0.693	0.398
	25-36165207-37329782	NRh	non-return rate heifer	0.0103	-0.352	0.953	0.712
	25-36165207-37329782	P1	survival period 1	0.0114	-1.496	3.435	0.663
	25-36165207-37329782	DFS	interval calving to insemination	0.0095	-0.419	0.626	0.504
	25-36165207-37329782	GLd	gestation length	0.0131	-1.335	1.277	0.296
	25-36165207-37329782	GLm	gestation length	0.0127	-2.915	1.473	**0.048**
	25-36165207-37329782	MBd	multiple birth	0.0109	0.755	1.811	0.677
	25-36165207-37329782	MBm	multiple birth	0.0110	-0.020	2.081	0.992
	25-36165207-37329782	IFLc	interval first to last insemination cow	0.0096	-1.062	0.578	0.066
	25-36165207-37329782	IFLh	interval first to last insemination heifer	0.0112	-2.768	1.781	0.120
**HH35**	26-3358717-4234871	P2b	survival bull period 2	0.0149	2.643	3.300	0.423
	26-3358717-4234871	BWd	birth weight	0.0135	2.201	1.369	0.108
	26-3358717-4234871	BWm	birth weight	0.0127	1.430	1.519	0.347
	26-3358717-4234871	P2h	survival heifer period 2	0.0092	-9.612	6.949	0.167
	26-3358717-4234871	SBd	percentage live births	0.0177	-1.453	2.184	0.506
	26-3358717-4234871	SBm	percentage live births	0.0158	-3.363	1.423	**0.018**
	26-3358717-4234871	CEd	percentage normal births	0.0147	0.529	0.460	0.250
	26-3358717-4234871	CEm	percentage normal births	0.0156	-0.046	0.774	0.953
	26-3358717-4234871	NRc	non-return rate cow	0.0142	0.147	0.712	0.836
	26-3358717-4234871	NRh	non-return rate heifer	0.0141	-0.251	0.956	0.793
	26-3358717-4234871	P1	survival period 1	0.0116	3.088	3.660	0.399
	26-3358717-4234871	DFS	interval calving to insemination	0.0138	-0.721	0.670	0.282
	26-3358717-4234871	GLd	gestation length	0.0151	2.537	1.342	0.059
	26-3358717-4234871	GLm	gestation length	0.0144	0.334	1.540	0.828
	26-3358717-4234871	MBd	multiple birth	0.0105	-0.985	1.929	0.610
	26-3358717-4234871	MBm	multiple birth	0.0028	0.606	4.119	0.883
	26-3358717-4234871	IFLc	interval first to last insemination cow	0.0136	-0.264	0.630	0.675

	26-3358717-4234871	IFLh	interval first to last insemination heifer	0.0046	-0.257	2.893	0.929
HH36	28-28541274-29736156	P2b	survival bull period 2	0.0060	8.188	4.631	0.077
	28-28541274-29736156	BWd	birth weight	0.0054	2.074	1.802	0.250
	28-28541274-29736156	BWm	birth weight	0.0045	-1.877	2.133	0.379
	28-28541274-29736156	P2h	survival heifer period 2	0.0051	1.671	8.383	0.842
	28-28541274-29736156	SBd	percentage live births	0.0041	0.378	3.563	0.916
	28-28541274-29736156	SBm	percentage live births	0.0016	1.069	3.274	0.744
	28-28541274-29736156	CEd	percentage normal births	0.0014	0.453	1.074	0.673
	28-28541274-29736156	CEm	percentage normal births	0.0014	4.873	1.919	**0.011**
	28-28541274-29736156	NRc	non-return rate cow	0.0010	1.589	2.029	0.434
	28-28541274-29736156	NRh	non-return rate heifer	0.0013	3.651	2.538	0.150
	28-28541274-29736156	P1	survival period 1	0.0050	5.673	5.092	0.265
	28-28541274-29736156	DFS	interval calving to insemination	0.0007	-0.299	2.110	0.887
	28-28541274-29736156	GLd	gestation length	0.0054	-0.013	1.809	0.994
	28-28541274-29736156	GLm	gestation length	0.0055	-0.363	2.055	0.860
	28-28541274-29736156	MBd	multiple birth	0.0057	-3.041	2.494	0.223
	28-28541274-29736156	MBm	multiple birth	0.0041	0.558	3.444	0.871
	28-28541274-29736156	IFLc	interval first to last insemination cow	0.0007	2.249	1.992	0.259
	28-28541274-29736156	IFLh	interval first to last insemination heifer	0.0027	-1.378	3.422	0.687
HH36	28-29731696-30877496	P2b	survival bull period 2	0.0093	-1.324	3.834	0.730
	28-29731696-30877496	BWd	birth weight	0.0089	0.240	1.464	0.870
	28-29731696-30877496	BWm	birth weight	0.0090	2.799	1.594	0.079
	28-29731696-30877496	P2h	survival heifer period 2	0.0074	-6.137	7.129	0.389
	28-29731696-30877496	SBd	percentage live births	0.0078	0.941	2.763	0.734
	28-29731696-30877496	SBm	percentage live births	0.0054	1.944	1.864	0.297
	28-29731696-30877496	CEd	percentage normal births	0.0044	-0.348	0.629	0.580
	28-29731696-30877496	CEm	percentage normal births	0.0048	1.947	1.078	0.071
	28-29731696-30877496	NRc	non-return rate cow	0.0046	-0.417	0.971	0.667
	28-29731696-30877496	NRh	non-return rate heifer	0.0045	1.544	1.398	0.269
	28-29731696-30877496	P1	survival period 1	0.0081	-3.359	4.104	0.413
	28-29731696-30877496	DFS	interval calving to insemination	0.0041	-0.418	0.921	0.650
	28-29731696-30877496	GLd	gestation length	0.0096	0.979	1.433	0.495
	28-29731696-30877496	GLm	gestation length	0.0088	2.218	1.709	0.194
	28-29731696-30877496	MBd	multiple birth	0.0116	0.735	1.823	0.687
	28-29731696-30877496	MBm	multiple birth	0.0074	4.502	2.586	0.082
	28-29731696-30877496	IFLc	interval first to last insemination cow	0.0038	-0.326	0.877	0.710
	28-29731696-30877496	IFLh	interval first to last insemination heifer	0.0044	0.171	2.789	0.951
	28-40347735-41271169	P2b	survival bull period 2	0.0098	5.278	3.552	0.137

**HH37**	28-40347735-41271169	BWd	birth weight	0.0097	-0.538	1.343	0.689
	28-40347735-41271169	BWm	birth weight	0.0084	-0.583	1.563	0.709
	28-40347735-41271169	P2h	survival heifer period 2	0.0051	3.884	8.472	0.647
	28-40347735-41271169	SBd	percentage live births	0.0076	7.988	2.652	**0.003**
	28-40347735-41271169	SBm	percentage live births	0.0026	2.618	2.662	0.325
	28-40347735-41271169	CEd	percentage normal births	0.0018	-0.166	0.961	0.863
	28-40347735-41271169	CEm	percentage normal births	0.0024	4.490	1.503	**0.003**
	28-40347735-41271169	NRc	non-return rate cow	0.0005	-1.039	2.760	0.706
	28-40347735-41271169	NRh	non-return rate heifer	0.0008	-1.581	3.126	0.613
	28-40347735-41271169	P1	survival period 1	0.0048	-7.852	5.078	0.122
	28-40347735-41271169	DFS	interval calving to insemination	0.0005	4.561	2.569	0.076
	28-40347735-41271169	GLd	gestation length	0.0120	3.437	1.225	**0.005**
	28-40347735-41271169	GLm	gestation length	0.0107	4.268	1.482	**0.004**
	28-40347735-41271169	MBd	multiple birth	0.0052	2.007	2.543	0.430
	28-40347735-41271169	MBm	multiple birth	0.0050	6.868	2.977	**0.021**
	28-40347735-41271169	IFLc	interval first to last insemination cow	0.0005	1.977	2.369	0.404
	28-40347735-41271169	IFLh	interval first to last insemination heifer	0.0020	-12.309	3.942	**0.002**
**HH38**	29-14242732-15316961	P2b	survival bull period 2	0.0065	-0.873	4.379	0.842
	29-14242732-15316961	BWd	birth weight	0.0051	0.113	1.825	0.951
	29-14242732-15316961	BWm	birth weight	0.0052	0.791	1.984	0.690
	29-14242732-15316961	P2h	survival heifer period 2	0.0046	-13.860	8.731	0.112
	29-14242732-15316961	SBd	percentage live births	0.0042	-1.896	3.566	0.595
	29-14242732-15316961	SBm	percentage live births	0.0017	-0.151	3.320	0.964
	29-14242732-15316961	CEd	percentage normal births	0.0012	-0.089	1.181	0.940
	29-14242732-15316961	CEm	percentage normal births	0.0014	3.911	1.966	**0.047**
	29-14242732-15316961	NRc	non-return rate cow	0.0007	0.063	2.386	0.979
	29-14242732-15316961	NRh	non-return rate heifer	0.0009	0.744	2.988	0.803
	29-14242732-15316961	P1	survival period 1	0.0052	-4.491	4.904	0.360
	29-14242732-15316961	DFS	interval calving to insemination	0.0006	2.400	2.402	0.318
	29-14242732-15316961	GLd	gestation length	0.0061	0.947	1.719	0.582
	29-14242732-15316961	GLm	gestation length	0.0059	-0.337	1.989	0.865
	29-14242732-15316961	MBd	multiple birth	0.0055	-1.001	2.499	0.689
	29-14242732-15316961	MBm	multiple birth	0.0030	-1.427	3.720	0.701
	29-14242732-15316961	IFLc	interval first to last insemination cow	0.0006	1.845	2.246	0.412
	29-14242732-15316961	IFLh	interval first to last insemination heifer	0.0024	-4.446	3.575	0.214
	7-7867806-10428419	P2b	survival bull period 2	0.1543	-2.555	1.190	**0.032**
	7-7867806-10428419	BWd	birth weight	0.1502	0.594	0.474	0.210
	7-7867806-10428419	BWm	birth weight	0.1508	0.818	0.519	0.115
	7-7867806-10428419	P2h	survival heifer period 2	0.1530	-1.704	2.041	0.404

HH21	7-7867806-10428419	SBd	percentage live births	0.1393	-1.241	0.862	0.150
	7-7867806-10428419	SBm	percentage live births	0.1473	0.494	0.523	0.345
	7-7867806-10428419	CEd	percentage normal births	0.1445	0.028	0.164	0.866
	7-7867806-10428419	CEm	percentage normal births	0.1493	0.323	0.286	0.258
	7-7867806-10428419	NRc	non-return rate cow	0.1522	-0.438	0.254	0.084
	7-7867806-10428419	NRh	non-return rate heifer	0.1456	-0.611	0.358	0.088
	7-7867806-10428419	P1	survival period 1	0.1537	-1.277	1.205	0.289
	7-7867806-10428419	DFS	interval calving to insemination	0.1520	-0.359	0.230	0.119
	7-7867806-10428419	GLd	gestation length	0.1495	0.867	0.479	0.070
	7-7867806-10428419	GLm	gestation length	0.1491	0.993	0.546	0.069
	7-7867806-10428419	MBd	multiple birth	0.1635	0.441	0.594	0.458
	7-7867806-10428419	MBm	multiple birth	0.1599	0.320	0.720	0.657
	7-7867806-10428419	IFLc	interval first to last insemination cow	0.1523	-0.383	0.214	0.073
	7-7867806-10428419	IFLh	interval first to last insemination heifer	0.1543	-0.581	0.631	0.357
HH21	7-7871925-10432630	P2b	survival bull period 2	0.0388	-1.866	1.886	0.322
	7-7871925-10432630	BWd	birth weight	0.0410	-0.314	0.693	0.651
	7-7871925-10432630	BWm	birth weight	0.0409	0.093	0.755	0.902
	7-7871925-10432630	P2h	survival heifer period 2	0.0467	1.506	2.984	0.614
	7-7871925-10432630	SBd	percentage live births	0.0478	0.108	1.144	0.925
	7-7871925-10432630	SBm	percentage live births	0.0571	0.484	0.647	0.454
	7-7871925-10432630	CEd	percentage normal births	0.0563	-0.178	0.201	0.375
	7-7871925-10432630	CEm	percentage normal births	0.0578	-0.119	0.351	0.736
	7-7871925-10432630	NRc	non-return rate cow	0.0582	-0.298	0.313	0.341
	7-7871925-10432630	NRh	non-return rate heifer	0.0559	-0.287	0.449	0.523
	7-7871925-10432630	P1	survival period 1	0.0409	0.072	1.866	0.969
	7-7871925-10432630	DFS	interval calving to insemination	0.0586	-0.034	0.283	0.903
	7-7871925-10432630	GLd	gestation length	0.0399	0.578	0.710	0.416
	7-7871925-10432630	GLm	gestation length	0.0405	0.289	0.809	0.721
	7-7871925-10432630	MBd	multiple birth	0.0356	0.594	1.026	0.562
	7-7871925-10432630	MBm	multiple birth	0.0314	1.507	1.243	0.225
	7-7871925-10432630	IFLc	interval first to last insemination cow	0.0578	-0.025	0.263	0.923
	7-7871925-10432630	IFLh	interval first to last insemination heifer	0.0364	0.332	0.995	0.738
	7-93581943-94642456	P2b	survival bull period 2	0.0096	4.373	3.722	0.240
	7-93581943-94642456	BWd	birth weight	0.0092	0.303	1.431	0.833
	7-93581943-94642456	BWm	birth weight	0.0082	-1.220	1.654	0.461
	7-93581943-94642456	P2h	survival heifer period 2	0.0074	2.097	7.396	0.777
	7-93581943-94642456	SBd	percentage live births	0.0064	2.189	3.060	0.474
	7-93581943-94642456	SBm	percentage live births	0.0025	1.121	2.888	0.698
	7-93581943-94642456	CEd	percentage normal births	0.0016	-0.041	1.061	0.969

HH22	7-93581943-94642456	CEm	percentage normal births	0.0021	-0.171	1.690	0.919
	7-93581943-94642456	NRc	non-return rate cow	0.0008	3.191	2.308	0.167
	7-93581943-94642456	NRh	non-return rate heifer	0.0009	2.958	3.156	0.349
	7-93581943-94642456	P1	survival period 1	0.0071	-2.619	4.462	0.557
	7-93581943-94642456	DFS	interval calving to insemination	0.0008	-0.753	2.209	0.733
	7-93581943-94642456	GLd	gestation length	0.0099	2.164	1.387	0.119
	7-93581943-94642456	GLm	gestation length	0.0099	2.146	1.583	0.175
	7-93581943-94642456	MBd	multiple birth	0.0098	-0.929	1.953	0.634
	7-93581943-94642456	MBm	multiple birth	0.0080	0.627	2.549	0.806
	7-93581943-94642456	IFLc	interval first to last insemination cow	0.0008	3.196	1.994	0.109
	7-93581943-94642456	IFLh	interval first to last insemination heifer	0.0038	-5.181	3.060	0.090
HH23	8-15561949-16862751	P2b	survival bull period 2	0.0072	1.454	4.161	0.727
	8-15561949-16862751	BWd	birth weight	0.0079	0.473	1.481	0.749
	8-15561949-16862751	BWm	birth weight	0.0066	-2.337	1.747	0.181
	8-15561949-16862751	P2h	survival heifer period 2	0.0060	-0.921	7.663	0.904
	8-15561949-16862751	SBd	percentage live births	0.0067	2.719	2.866	0.343
	8-15561949-16862751	SBm	percentage live births	0.0039	-2.059	2.235	0.357
	8-15561949-16862751	CEd	percentage normal births	0.0029	-0.806	0.772	0.296
	8-15561949-16862751	CEm	percentage normal births	0.0035	1.644	1.273	0.196
	8-15561949-16862751	NRc	non-return rate cow	0.0026	0.506	1.305	0.698
	8-15561949-16862751	NRh	non-return rate heifer	0.0026	-1.550	1.857	0.404
	8-15561949-16862751	P1	survival period 1	0.0050	3.488	4.997	0.485
	8-15561949-16862751	DFS	interval calving to insemination	0.0022	1.173	1.260	0.352
	8-15561949-16862751	GLd	gestation length	0.0090	2.440	1.413	0.084
	8-15561949-16862751	GLm	gestation length	0.0084	2.147	1.675	0.200
	8-15561949-16862751	MBd	multiple birth	0.0050	0.437	2.607	0.867
	8-15561949-16862751	MBm	multiple birth	0.0058	7.089	2.746	**0.010**
	8-15561949-16862751	IFLc	interval first to last insemination cow	0.0021	-0.091	1.183	0.939
	8-15561949-16862751	IFLh	interval first to last insemination heifer	0.0037	-2.680	2.944	0.362
24	8-66699044-68005926	P2b	survival bull period 2	0.0128	-1.832	3.207	0.568
	8-66699044-68005926	BWd	birth weight	0.0129	1.025	1.189	0.389
	8-66699044-68005926	BWm	birth weight	0.0122	-2.775	1.325	**0.036**
	8-66699044-68005926	P2h	survival heifer period 2	0.0106	4.589	5.874	0.435
	8-66699044-68005926	SBd	percentage live births	0.0125	-0.004	2.088	0.998
	8-66699044-68005926	SBm	percentage live births	0.0096	0.015	1.350	0.991
	8-66699044-68005926	CEd	percentage normal births	0.0131	0.246	0.368	0.504
	8-66699044-68005926	CEm	percentage normal births	0.0107	1.585	0.711	**0.026**
	8-66699044-68005926	NRc	non-return rate cow	0.0089	-0.269	0.682	0.693
	8-66699044-68005926	NRh	non-return rate heifer	0.0117	-2.011	0.868	**0.020**

HH[illegible]	8-66699044-68005926	P1	survival period 1	0.0135	-0.564	3.144	0.858
	8-66699044-68005926	DFS	interval calving to insemination	0.0095	1.012	0.601	0.092
	8-66699044-68005926	GLd	gestation length	0.0130	0.111	1.198	0.926
	8-66699044-68005926	GLm	gestation length	0.0127	-1.470	1.390	0.290
	8-66699044-68005926	MBd	multiple birth	0.0085	-0.308	2.022	0.879
	8-66699044-68005926	MBm	multiple birth	0.0052	6.693	2.914	**0.022**
	8-66699044-68005926	IFLc	interval first to last insemination cow	0.0091	-0.169	0.567	0.766
	8-66699044-68005926	IFLh	interval first to last insemination heifer	0.0064	0.286	2.222	0.898
HH3	8-90958661-92084831	P2b	survival bull period 2	0.0103	-4.902	3.696	0.185
	8-90958661-92084831	BWd	birth weight	0.0098	0.417	1.481	0.778
	8-90958661-92084831	BWm	birth weight	0.0099	-1.201	1.623	0.460
	8-90958661-92084831	P2h	survival heifer period 2	0.0120	5.618	5.923	0.343
	8-90958661-92084831	SBd	percentage live births	0.0172	0.066	2.199	0.976
	8-90958661-92084831	SBm	percentage live births	0.0198	-0.275	1.424	0.847
	8-90958661-92084831	CEd	percentage normal births	0.0185	0.120	0.475	0.800
	8-90958661-92084831	CEm	percentage normal births	0.0194	0.360	0.778	0.644
	8-90958661-92084831	NRc	non-return rate cow	0.0201	-0.799	0.694	0.250
	8-90958661-92084831	NRh	non-return rate heifer	0.0180	-1.737	0.937	0.064
	8-90958661-92084831	P1	survival period 1	0.0116	-1.049	3.544	0.767
	8-90958661-92084831	DFS	interval calving to insemination	0.0199	-0.354	0.656	0.590
	8-90958661-92084831	GLd	gestation length	0.0104	-0.404	1.471	0.784
	8-90958661-92084831	GLm	gestation length	0.0096	-3.566	1.734	**0.040**
	8-90958661-92084831	MBd	multiple birth	0.0079	-4.162	2.150	0.053
	8-90958661-92084831	MBm	multiple birth	0.0067	4.195	2.686	0.118
	8-90958661-92084831	IFLc	interval first to last insemination cow	0.0199	-0.672	0.618	0.277
	8-90958661-92084831	IFLh	interval first to last insemination heifer	0.0081	1.032	2.146	0.631
HH5	9-90928417-91823561	P2b	survival bull period 2	0.0142	-1.899	3.329	0.568
	9-90928417-91823561	BWd	birth weight	0.0174	-1.480	1.284	0.249
	9-90928417-91823561	BWm	birth weight	0.0161	-1.009	1.430	0.481
	9-90928417-91823561	P2h	survival heifer period 2	0.0152	-6.484	5.629	0.249
	9-90928417-91823561	SBd	percentage live births	0.0212	3.631	2.161	0.093
	9-90928417-91823561	SBm	percentage live births	0.0209	-0.067	1.548	0.966
	9-90928417-91823561	CEd	percentage normal births	0.0185	0.605	0.529	0.252
	9-90928417-91823561	CEm	percentage normal births	0.0193	0.641	0.871	0.462
	9-90928417-91823561	NRc	non-return rate cow	0.0194	-1.184	0.778	0.128
	9-90928417-91823561	NRh	non-return rate heifer	0.0184	-3.973	0.985	######
	9-90928417-91823561	P1	survival period 1	0.0168	1.154	3.135	0.713
	9-90928417-91823561	DFS	interval calving to insemination	0.0175	1.289	0.767	0.093

9-90928417-91823561	GLd	gestation length	0.0168	-1.340	1.302	0.303
9-90928417-91823561	GLm	gestation length	0.0168	-0.900	1.487	0.545
9-90928417-91823561	MBd	multiple birth	0.0155	3.005	1.644	0.068
9-90928417-91823561	MBm	multiple birth	0.0100	-3.082	2.498	0.217
9-90928417-91823561	IFLc	interval first to last insemination cow	0.0172	-0.188	0.725	0.796
9-90928417-91823561	IFLh	interval first to last insemination heifer	0.0092	2.513	2.193	0.252

Supplementary Table S5: Significant (p<0.0000043) GWAS results in Swiss Holstein cattle.

trait	trait description	chr	pos	freq	b-value	var	p-value
IFLc	interval first to last insemination cow	1	69033809	0.8974	4.77944	4.20658	4.23E-07
IFLc	interval first to last insemination cow	1	69047241	0.1023	-4.76315	4.16728	5.02E-07
NRc	non-return rate cow	1	69047241	0.106	-4.73796	4.25449	3.29E-06
P2h	survival heifer period 2	1	149719167	0.0175	-46.6798	74.9974	1.64E-06
CEd	percentage normal births	2	107940480	0.2898	1.94556	1.55824	1.52E-06
P1	survival period 1	2	134467046	0.9416	16.2273	28.9816	3.18E-06
SBd	percentage live births	3	19337047	0.3526	7.33627	24.5726	8.68E-07
DFS	interval calving to insemination	5	88267557	0.1925	3.04614	2.88439	2.09E-07
DFS	interval calving to insemination	5	88360524	0.542	-3.12001	4.83292	3.83E-10
IFLc	interval first to last insemination cow	5	88360524	0.5443	-2.51609	3.14048	9.19E-08
DFS	interval calving to insemination	5	88412132	0.5381	-3.21249	5.13007	1.54E-11
IFLc	interval first to last insemination cow	5	88412132	0.5361	-2.13845	2.27456	1.92E-06
DFS	interval calving to insemination	5	88432158	0.5625	2.41095	2.86089	3.90E-07
DFS	interval calving to insemination	5	88527468	0.6533	-2.76449	3.46211	5.93E-08
DFS	interval calving to insemination	5	88602535	0.8915	3.45718	2.31189	1.99E-06
CEd	percentage normal births	5	105301690	0.1774	2.37815	1.6507	2.86E-06
CEd	percentage normal births	5	105408595	0.6768	-2.16924	2.05865	7.63E-08
CEd	percentage normal births	5	105515535	0.7425	-1.90211	1.38332	3.97E-06
CEd	percentage normal births	5	105650306	0.4855	2.36604	2.79673	3.67E-07
CEd	percentage normal births	5	105683327	0.4727	2.18739	2.3852	3.76E-06
CEd	percentage normal births	5	105708881	0.4717	2.24588	2.51392	1.49E-06
CEd	percentage normal births	5	105743393	0.1155	2.92756	1.75095	6.25E-08
CEd	percentage normal births	5	105756724	0.8644	-3.20507	2.40786	5.93E-10
IFLc	interval first to last insemination cow	5	105756724	0.8645	-3.20189	2.40132	1.96E-06
CEd	percentage normal births	5	105760018	0.8645	-3.23479	2.45217	4.13E-10
IFLc	interval first to last insemination cow	5	105760018	0.8647	-3.22061	2.42759	1.72E-06
CEd	percentage normal births	5	105771330	0.1341	3.35265	2.60991	8.23E-11
CEd	percentage normal births	5	105773809	0.135	3.23064	2.4369	4.38E-10
IFLc	interval first to last insemination cow	5	105773809	0.1347	3.27827	2.50551	1.14E-06
CEd	percentage normal births	5	105776475	0.1197	3.1708	2.11819	4.86E-09
CEd	percentage normal births	5	105779368	0.8645	-3.14902	2.32261	1.19E-09
IFLc	interval first to last insemination cow	5	105779368	0.8648	-3.23661	2.45034	1.56E-06

CEd	percentage normal births	5	105802469	0.8	-2.15009	1.47911	1.40E-06
CEd	percentage normal births	5	105804880	0.8001	-2.15509	1.48559	1.35E-06
SBd	percentage live births	5	108807160	0.0213	-16.6917	11.6289	3.54E-06
BWm	birth weight	6	29366667	0.6162	-3.90547	7.21441	3.32E-06
BWd	birth weight	6	36857907	0.8725	-5.62137	7.02941	5.57E-07
BWd	birth weight	6	36863269	0.873	-5.18038	5.95091	4.02E-06
BWd	birth weight	6	36871343	0.1275	5.31656	6.28777	1.99E-06
BWd	birth weight	6	37403795	0.9706	-16.0898	14.7714	2.37E-16
BWm	birth weight	6	37403795	0.9686	-11.9878	8.75224	1.73E-08
BWd	birth weight	6	39956254	0.9791	-10.2806	4.32666	1.60E-06
IFLc	interval first to last insemination cow	6	86860291	0.4506	-3.07327	4.67639	1.24E-09
DFS	interval calving to insemination	6	86860291	0.4437	-2.52963	3.15901	2.28E-06
IFLc	interval first to last insemination cow	6	86877334	0.5485	2.80286	3.89108	2.28E-08
DFS	interval calving to insemination	6	86924174	0.3099	2.78888	3.32691	4.03E-07
DFS	interval calving to insemination	6	86936145	0.6874	-2.55622	2.8084	1.56E-06
DFS	interval calving to insemination	6	86940863	0.5527	4.92791	12.0073	3.86E-19
IFLc	interval first to last insemination cow	6	86940863	0.5468	3.52077	6.14366	1.67E-11
CEm	percentage normal births	6	86940863	0.569	3.34844	5.49922	1.54E-07
DFS	interval calving to insemination	6	86989953	0.286	2.63626	2.83853	2.74E-06
DFS	interval calving to insemination	6	86992277	0.2852	2.74068	3.06247	1.21E-06
DFS	interval calving to insemination	6	87003480	0.6828	-3.12783	4.23789	3.61E-09
DFS	interval calving to insemination	6	87021332	0.6535	-2.96865	3.99121	1.76E-08
DFS	interval calving to insemination	6	87068809	0.4889	3.89563	7.5842	3.71E-13
IFLc	interval first to last insemination cow	6	87068809	0.4829	2.67017	3.56072	1.34E-07
DFS	interval calving to insemination	6	87090740	0.3193	2.7051	3.18115	3.69E-06
DFS	interval calving to insemination	6	87184768	0.4252	-5.02447	12.3403	8.80E-18
IFLc	interval first to last insemination cow	6	87184768	0.4312	-3.59539	6.34108	8.73E-11
DFS	interval calving to insemination	6	87213962	0.5	-3.28113	5.38291	5.84E-10
IFLc	interval first to last insemination cow	6	87213962	0.5043	-2.57098	3.30472	2.80E-07
DFS	interval calving to insemination	6	87224275	0.711	-2.81423	3.25445	8.40E-08
DFS	interval calving to insemination	6	87324678	0.5758	4.31928	9.11373	5.13E-14
IFLc	interval first to last insemination cow	6	87324678	0.5695	2.89274	4.10308	9.64E-08
NRc	non-return rate cow	6	98196576	0.9512	-4.73151	2.07661	3.35E-06
NRh	non-return rate heifer	6	101541974	0.7762	4.41131	6.76162	7.05E-10
NRh	non-return rate heifer	6	101564868	0.4729	-3.12493	4.86822	2.51E-07
NRh	non-return rate heifer	6	102017062	0.267	-3.41854	4.57423	7.67E-08
CEm	percentage normal births	6	110482099	0.6273	-2.82525	3.73219	2.92E-07
CEm	percentage normal births	6	110714073	0.1949	-3.68073	4.25172	4.81E-07

CEm	percentage normal births	7	46396334	0.6315	-3.69129	6.34177	2.21E-06
CEd	percentage normal births	7	90622467	0.9764	4.18709	0.80944	4.69E-07
CEd	percentage normal births	7	90851664	0.9136	3.18088	1.59754	4.23E-10
CEd	percentage normal births	7	90855265	0.0663	-4.42143	2.41881	3.74E-14
NRc	non-return rate cow	7	90855265	0.0698	-4.38992	2.50398	1.17E-07
IFLc	interval first to last insemination cow	7	90855265	0.0735	-3.55809	1.72317	2.22E-06
CEd	percentage normal births	7	90882079	0.8892	2.42317	1.15731	1.13E-07
CEd	percentage normal births	7	90887932	0.9336	4.43208	2.43508	2.56E-14
NRc	non-return rate cow	7	90887932	0.93	4.47941	2.61086	5.87E-08
IFLc	interval first to last insemination cow	7	90887932	0.9265	3.57993	1.74643	1.78E-06
CEd	percentage normal births	7	90966315	0.4206	-1.97243	1.8962	7.02E-07
SBm	percentage live births	8	90073503	0.0891	-7.8033	9.88	1.89E-06
SBm	percentage live births	8	90099064	0.9109	7.89931	10.1293	1.47E-06
NRh	non-return rate heifer	9	50198332	0.8559	5.60323	7.74657	1.71E-06
CEd	percentage normal births	10	12252829	0.9186	2.99722	1.34335	9.48E-08
CEd	percentage normal births	10	12271119	0.082	-3.04302	1.39387	4.85E-08
CEd	percentage normal births	10	12271909	0.918	3.04302	1.39387	4.85E-08
GLm	gestation length	10	13391218	0.5122	3.72298	6.92617	4.04E-06
GLm	gestation length	10	13433297	0.5101	3.77165	7.10975	3.18E-06
SBd	percentage live births	11	10060004	0.098	-9.15597	14.8188	2.05E-06
MBm	multiple birth	11	31004983	0.5313	5.25488	13.7527	1.23E-07
MBm	multiple birth	11	31056487	0.4956	-4.95885	12.2941	4.25E-07
MBm	multiple birth	11	37136773	0.8691	-6.3751	9.24598	3.00E-06
P2h	survival heifer period 2	11	72258221	0.9339	26.1006	84.1489	3.60E-06
P2h	survival heifer period 2	11	72280075	0.0542	-35.8673	131.856	4.90E-08
P2h	survival heifer period 2	11	77958994	0.0503	-43.6852	182.257	5.65E-11
CEm	percentage normal births	11	78311315	0.6201	2.87645	3.89824	2.37E-07
BWd	birth weight	14	22797143	0.0259	-9.11929	4.18935	2.94E-06
BWd	birth weight	14	22802830	0.9741	9.11929	4.18935	2.94E-06
BWd	birth weight	14	22830132	0.9809	10.3791	4.03605	2.77E-06
BWd	birth weight	14	22899088	0.0281	-10.9614	6.55315	5.28E-09
BWd	birth weight	14	22906023	0.9847	11.2178	3.79794	3.98E-06
BWd	birth weight	14	22916046	0.9635	8.0847	4.60277	1.12E-06
BWd	birth weight	14	22933624	0.0277	-11.0909	6.61752	4.99E-09
BWd	birth weight	14	22999212	0.0892	-5.9995	5.84863	3.54E-07
BWd	birth weight	14	23039779	0.9721	10.6759	6.18236	4.21E-08
BWd	birth weight	14	23043305	0.0267	-10.5456	5.78444	9.92E-08
BWd	birth weight	14	23047927	0.0267	-10.5456	5.78444	9.92E-08
BWd	birth weight	14	23060216	0.9792	10.0754	4.14035	3.77E-06
BWd	birth weight	14	23060870	0.9722	10.6777	6.16754	4.34E-08
BWd	birth weight	14	23081092	0.0211	-10.9456	4.95863	5.02E-07
BWd	birth weight	14	23084813	0.0212	-11.0035	5.02942	4.18E-07
BWd	birth weight	14	23086563	0.0212	-11.0035	5.02942	4.18E-07

BWd	birth weight	14	23088684	0.9786	11.07	5.12725	3.03E-07
BWd	birth weight	14	23095328	0.9726	10.5646	5.95466	7.38E-08
BWd	birth weight	14	23102372	0.0212	-11.0035	5.02942	4.18E-07
BWd	birth weight	14	23232498	0.07	-7.50141	7.32894	8.56E-09
CEd	percentage normal births	14	23232498	0.0316	3.91488	0.938341	1.57E-08
CEd	percentage normal births	14	23262536	0.015	4.93483	0.717373	2.75E-07
BWd	birth weight	14	23262536	0.0412	-7.50569	4.44892	2.65E-06
CEd	percentage normal births	14	23285130	0.0151	5.03727	0.752905	1.41E-07
BWd	birth weight	14	23285130	0.0417	-7.62025	4.64436	1.76E-06
BWd	birth weight	14	23321577	0.966	8.51867	4.76028	2.14E-06
BWd	birth weight	14	23389588	0.962	9.453	6.52659	5.09E-08
BWd	birth weight	14	23575960	0.9848	11.3247	3.83161	2.68E-06
BWd	birth weight	14	23617053	0.9797	10.6633	4.51765	4.34E-07
BWd	birth weight	14	23622754	0.0234	-9.58049	4.19861	2.38E-06
BWd	birth weight	14	23630896	0.0261	-10.5782	5.68701	5.23E-08
BWd	birth weight	14	23639458	0.9767	9.71799	4.30584	1.22E-06
BWd	birth weight	14	23652804	0.0224	-12.0853	6.39631	5.78E-09
P1	survival period 1	15	57184911	0.386	-8.09169	31.0346	3.38E-06
CEd	percentage normal births	17	67330305	0.9011	2.70764	1.3064	1.61E-06
CEd	percentage normal births	17	68274420	0.5043	-1.7355	1.50588	2.40E-06
CEd	percentage normal births	17	68292838	0.5632	1.82346	1.63592	1.89E-07
CEd	percentage normal births	17	68308036	0.5043	-1.73365	1.50265	2.44E-06
CEd	percentage normal births	17	68825904	0.9038	3.22505	1.80924	6.55E-07
CEd	percentage normal births	17	69163976	0.0962	-3.3708	1.97529	1.73E-07
IFLc	interval first to last insemination cow	17	69230601	0.4261	2.30624	2.60122	7.35E-07
DFS	interval calving to insemination	17	69259482	0.3612	2.82006	3.66989	1.01E-08
NRc	non-return rate cow	17	69259482	0.3614	2.34125	2.53004	2.89E-06
IFLc	interval first to last insemination cow	17	69259482	0.3615	2.15301	2.13982	3.60E-06
DFS	interval calving to insemination	17	69283790	0.3254	2.82387	3.50113	5.10E-08
IFLc	interval first to last insemination cow	17	69283790	0.326	2.53954	2.83414	2.14E-07
DFS	interval calving to insemination	17	69337705	0.6046	-2.34973	2.63969	2.90E-06
DFS	interval calving to insemination	17	69351312	0.41	2.59047	3.24661	2.39E-07
CEd	percentage normal births	17	69986216	0.8948	3.04802	1.74846	1.18E-07
SBm	percentage live births	17	72670466	0.7917	5.43382	9.73703	1.56E-06
SBm	percentage live births	17	72697904	0.203	-5.37397	9.34603	3.05E-06
DFS	interval calving to insemination	18	42598286	0.8559	2.86074	2.01857	7.89E-08
IFLc	interval first to last insemination cow	18	43854199	0.8454	2.78517	2.02739	3.59E-06
IFLc	interval first to last insemination cow	18	43887966	0.8428	2.91637	2.25392	8.38E-07
DFS	interval calving to insemination	18	44177327	0.8963	7.70306	11.0323	2.05E-21

IFLc	interval first to last insemination cow	18	44177327	0.8942	6.49561	7.9842	1.83E-17
DFS	interval calving to insemination	18	44323486	0.6044	-2.3653	2.67541	2.51E-07
DFS	interval calving to insemination	18	44383419	0.3242	-2.35892	2.43823	7.02E-07
DFS	interval calving to insemination	18	44724263	0.9002	3.79183	2.58362	4.84E-07
IFLc	interval first to last insemination cow	18	44724263	0.8974	3.53492	2.30196	5.84E-07
DFS	interval calving to insemination	18	44765510	0.9017	4.49897	3.58793	8.10E-09
IFLc	interval first to last insemination cow	18	44765510	0.8988	4.02189	2.94295	3.92E-08
DFS	interval calving to insemination	18	45060867	0.09	-5.99245	5.88127	1.15E-11
IFLc	interval first to last insemination cow	18	45060867	0.0929	-5.07989	4.34742	9.18E-10
IFLc	interval first to last insemination cow	18	45542297	0.0997	-5.42941	5.29141	4.32E-13
DFS	interval calving to insemination	18	45542297	0.0961	-5.41603	5.09592	1.52E-11
IFLc	interval first to last insemination cow	18	45697720	0.129	-3.56083	2.84899	2.54E-08
DFS	interval calving to insemination	18	45697720	0.1251	-3.43465	2.58192	4.53E-07
DFS	interval calving to insemination	18	45744538	0.3575	-2.30753	2.44611	1.14E-06
DFS	interval calving to insemination	18	46510336	0.625	2.9036	3.95198	1.79E-06
DFS	interval calving to insemination	18	46515971	0.6252	2.94422	4.06257	1.27E-06
DFS	interval calving to insemination	18	46523750	0.3741	-3.00799	4.23709	6.76E-07
DFS	interval calving to insemination	18	46524464	0.6261	2.98032	4.15887	8.18E-07
DFS	interval calving to insemination	18	47927237	0.8976	4.26643	3.34631	2.90E-07
GLm	gestation length	18	54816033	0.2689	4.74451	8.85177	1.27E-06
BWd	birth weight	18	55556538	0.8868	-7.33921	10.812	5.36E-09
CEd	percentage normal births	18	55556538	0.9459	3.75228	1.44073	5.81E-09
CEd	percentage normal births	18	56539007	0.7802	3.27459	3.67739	3.74E-16
BWd	birth weight	18	56539007	0.7196	-4.44415	7.96999	1.71E-07
GLd	gestation length	18	56539007	0.7205	-4.10962	6.8027	8.35E-07
CEd	percentage normal births	18	56559911	0.9155	6.14918	5.85247	2.04E-28
CEm	percentage normal births	18	56559911	0.9148	6.98	7.59159	6.42E-15
BWd	birth weight	18	56559911	0.8528	-7.2949	13.3607	1.03E-11
NRc	non-return rate cow	18	56559911	0.9111	4.0433	2.64877	2.03E-07
BWm	birth weight	18	56559911	0.8488	-5.91876	8.9922	5.17E-07
GLd	gestation length	18	56559911	0.8535	-5.08535	6.46675	1.31E-06
SBm	percentage live births	18	56559911	0.9119	7.73311	9.61164	1.58E-06
CEd	percentage normal births	18	56560944	0.8345	3.20183	2.83168	6.56E-13
BWd	birth weight	18	56560944	0.7957	-5.20535	8.80812	9.09E-09
CEd	percentage normal births	18	56567219	0.67	1.86704	1.54144	4.71E-08
BWd	birth weight	18	56567219	0.6093	-3.82354	6.96028	3.27E-07
CEm	percentage normal births	18	56567219	0.6651	2.73496	3.33198	8.94E-07
CEd	percentage normal births	18	56652412	0.194	-2.81204	2.47268	5.29E-12
CEm	percentage normal births	18	56652412	0.1992	-3.14505	3.15583	2.15E-06

CEd	percentage normal births	18	56679475	0.7184	-1.81635	1.33485	1.13E-06
CEd	percentage normal births	18	56716572	0.7247	2.28995	2.0925	1.10E-08
BWd	birth weight	18	56716572	0.7435	-4.6132	8.11763	5.68E-08
BWm	birth weight	18	56716572	0.7421	-4.37902	7.33929	3.26E-06
CEd	percentage normal births	18	56780040	0.3549	-2.70388	3.34764	1.49E-14
GLd	gestation length	18	56780040	0.3375	4.71681	9.94984	5.95E-10
BWd	birth weight	18	56780040	0.3366	4.42013	8.72525	1.13E-08
BWm	birth weight	18	56780040	0.3358	4.58401	9.37301	8.56E-08
CEd	percentage normal births	18	57032285	0.7809	3.29788	3.72122	5.44E-14
CEd	percentage normal births	18	57057494	0.9279	7.72536	7.98914	2.21E-40
CEm	percentage normal births	18	57057494	0.9259	6.47736	5.75699	5.95E-12
BWd	birth weight	18	57057494	0.8553	-7.42343	13.6394	1.44E-11
BWm	birth weight	18	57057494	0.8525	-6.73744	11.414	2.90E-08
SBm	percentage live births	18	57057494	0.9221	9.25134	12.2974	4.36E-08
IFLc	interval first to last insemination cow	18	57057494	0.927	3.70929	1.86174	9.54E-07
NRc	non-return rate cow	18	57057494	0.9244	4.03142	2.27142	1.04E-06
GLd	gestation length	18	57057494	0.855	-5.152	6.5819	1.60E-06
GLm	gestation length	18	57057494	0.855	-5.67123	7.97412	2.96E-06
CEd	percentage normal births	18	57077568	0.0657	-10.5384	13.6372	2.75E-57
CEm	percentage normal births	18	57077568	0.0668	-9.48568	11.219	3.38E-19
BWd	birth weight	18	57077568	0.1224	9.50616	19.4204	1.14E-14
BWm	birth weight	18	57077568	0.126	8.92199	17.5346	4.74E-11
IFLc	interval first to last insemination cow	18	57077568	0.0675	-5.32075	3.56296	4.15E-10
SBm	percentage live births	18	57077568	0.0698	-11.162	16.173	4.84E-09
NRc	non-return rate cow	18	57077568	0.0695	-5.23659	3.54456	1.07E-08
GLm	gestation length	18	57077568	0.1219	7.7026	12.7032	1.61E-08
GLd	gestation length	18	57077568	0.1215	6.62554	9.37037	4.29E-08
SBd	percentage live births	18	57077568	0.0861	-12.8935	26.1747	2.87E-07
CEd	percentage normal births	18	57106651	0.6622	2.06308	1.90417	4.14E-08
CEd	percentage normal births	18	57127311	0.2669	-3.34799	4.38643	7.67E-18
BWd	birth weight	18	57127311	0.3466	3.85683	6.73767	1.26E-06
CEd	percentage normal births	18	57136340	0.2463	-3.83754	5.46824	9.56E-21
CEm	percentage normal births	18	57136340	0.2461	-3.36468	4.2005	3.13E-07
CEd	percentage normal births	18	57137406	0.2463	-3.82721	5.43829	1.27E-20
CEm	percentage normal births	18	57137406	0.246	-3.37446	4.22474	2.91E-07
CEd	percentage normal births	18	57138872	0.2463	-3.83023	5.44708	1.19E-20
CEm	percentage normal births	18	57138872	0.246	-3.37446	4.22474	2.91E-07
CEd	percentage normal births	18	57139571	0.0658	-10.3493	13.1683	1.75E-56
CEm	percentage normal births	18	57139571	0.0669	-9.03916	10.1979	5.74E-18
BWd	birth weight	18	57139571	0.1227	9.8487	20.8825	1.09E-15
BWm	birth weight	18	57139571	0.126	9.25105	18.8547	9.06E-12
GLm	gestation length	18	57139571	0.122	8.37305	15.0203	7.47E-10
GLd	gestation length	18	57139571	0.1216	7.15903	10.9523	2.93E-09

IFLc	interval first to last insemination cow	18	57139571	0.0674	-4.90462	3.02615	5.81E-09
SBm	percentage live births	18	57139571	0.0699	-10.8628	15.3411	8.55E-09
NRc	non-return rate cow	18	57139571	0.0696	-4.55497	2.68573	4.88E-07
SBd	percentage live births	18	57139571	0.0863	-12.5063	24.66	5.75E-07
CEd	percentage normal births	18	57139575	0.9342	10.3493	13.1683	1.75E-56
CEm	percentage normal births	18	57139575	0.9331	9.03916	10.1979	5.74E-18
BWd	birth weight	18	57139575	0.8773	-9.8487	20.8825	1.09E-15
BWm	birth weight	18	57139575	0.874	-9.25105	18.8547	9.06E-12
GLm	gestation length	18	57139575	0.878	-8.37305	15.0203	7.47E-10
GLd	gestation length	18	57139575	0.8784	-7.15903	10.9523	2.93E-09
IFLc	interval first to last insemination cow	18	57139575	0.9326	4.90462	3.02615	5.81E-09
SBm	percentage live births	18	57139575	0.9301	10.8628	15.3411	8.55E-09
NRc	non-return rate cow	18	57139575	0.9304	4.55497	2.68573	4.88E-07
SBd	percentage live births	18	57139575	0.9137	12.5063	24.66	5.75E-07
CEd	percentage normal births	18	57151314	0.3313	-2.17489	2.0958	6.27E-10
GLm	gestation length	18	57151314	0.3621	4.69558	10.1861	2.55E-08
CEm	percentage normal births	18	57151314	0.3412	-3.06008	4.20958	7.12E-08
BWd	birth weight	18	57151314	0.3633	3.94976	7.21732	1.93E-07
BWm	birth weight	18	57151314	0.3655	3.86346	6.92337	4.18E-06
CEd	percentage normal births	18	57718863	0.566	1.69741	1.41553	1.43E-06
CEd	percentage normal births	18	57741397	0.7012	4.40735	8.13929	3.18E-28
BWd	birth weight	18	57741397	0.6826	-3.93108	6.69653	2.05E-06
CEd	percentage normal births	18	57780453	0.3544	2.33367	2.49214	4.49E-09
CEd	percentage normal births	18	57780512	0.6455	-2.41612	2.67163	1.68E-09
CEd	percentage normal births	18	57836752	0.6412	-2.28051	2.39312	1.21E-08
CEd	percentage normal births	18	57893557	0.7723	3.43072	4.13942	1.55E-16
BWd	birth weight	18	57893557	0.7313	-5.52327	11.9904	3.96E-11
CEm	percentage normal births	18	57893557	0.7667	3.11222	3.46473	2.26E-06
CEd	percentage normal births	18	57916024	0.7243	2.2844	2.08397	7.16E-09
BWd	birth weight	18	57916024	0.691	-3.68176	5.78826	3.49E-06
BWd	birth weight	18	57916335	0.3891	3.55419	6.00526	1.26E-06
CEd	percentage normal births	18	57967581	0.5946	1.87905	1.70216	1.21E-06
CEd	percentage normal births	18	57993345	0.5959	2.80096	3.77831	1.47E-13
CEd	percentage normal births	18	58065996	0.6082	3.0994	4.57835	6.02E-14
BWd	birth weight	18	58065996	0.6012	-4.64987	10.3675	1.93E-09
CEd	percentage normal births	18	58156599	0.7244	2.78698	3.10109	4.93E-11
GLd	gestation length	18	58156599	0.7315	-4.43257	7.71803	1.26E-07
BWm	birth weight	18	58156599	0.7305	-4.95511	9.66747	1.88E-07
BWd	birth weight	18	58156599	0.7325	-4.29457	7.22752	5.41E-07
CEd	percentage normal births	18	58213698	0.7242	2.82298	3.18332	2.68E-11
BWd	birth weight	18	58213698	0.7351	-4.54467	8.04375	1.12E-07
BWm	birth weight	18	58213698	0.7341	-4.89516	9.35373	2.57E-07
CEd	percentage normal births	18	58303038	0.7184	-2.55516	2.64132	3.46E-08

CEd	percentage normal births	18	58328337	0.6403	3.03431	4.24129	1.74E-12
BWm	birth weight	18	58328337	0.6416	-5.20981	12.4831	7.31E-09
BWd	birth weight	18	58328337	0.6444	-4.47628	9.18325	2.98E-08
CEd	percentage normal births	18	58337557	0.5727	2.18611	2.33906	2.20E-08
BWd	birth weight	18	58337557	0.5601	-3.71572	6.80348	1.07E-06
BWm	birth weight	18	58337557	0.5573	-4.06836	8.16702	1.66E-06
CEd	percentage normal births	18	58672622	0.3874	-2.19922	2.29559	1.72E-08
BWm	birth weight	18	58672622	0.3818	4.16917	8.2051	1.46E-06
BWd	birth weight	18	58672622	0.3789	3.60072	6.10262	3.66E-06
CEd	percentage normal births	18	58968919	0.6382	2.55223	3.00825	9.06E-10
BWm	birth weight	18	58968919	0.6182	-4.5484	9.76566	2.77E-07
BWd	birth weight	18	58968919	0.6185	-4.01134	7.59386	4.37E-07
CEd	percentage normal births	18	59051322	0.6479	2.87713	3.77681	9.98E-14
CEd	percentage normal births	18	59186046	0.2687	2.34612	2.16302	1.37E-07
CEd	percentage normal births	18	59319065	0.22	-3.89435	5.20436	1.27E-19
CEm	percentage normal births	18	59319065	0.2282	-3.74157	4.93191	5.24E-08
BWd	birth weight	18	59319065	0.3121	4.36411	8.17727	1.98E-07
BWm	birth weight	18	59319065	0.3146	4.59457	9.10337	8.07E-07
CEd	percentage normal births	18	59319876	0.8434	5.49078	7.96301	2.06E-29
BWm	birth weight	18	59319876	0.7811	-7.36296	18.5368	1.60E-12
BWd	birth weight	18	59319876	0.7823	-6.48116	14.3076	4.24E-12
CEm	percentage normal births	18	59319876	0.8392	5.37711	7.80527	4.79E-12
IFLc	interval first to last insemination cow	18	59319876	0.8362	3.59126	3.53318	1.44E-08
NRc	non-return rate cow	18	59319876	0.8373	3.57774	3.48832	1.61E-07
CEd	percentage normal births	18	59319899	0.1566	-5.49078	7.96301	2.06E-29
BWm	birth weight	18	59319899	0.2189	7.36296	18.5368	1.60E-12
BWd	birth weight	18	59319899	0.2177	6.48116	14.3076	4.24E-12
CEm	percentage normal births	18	59319899	0.1608	-5.37711	7.80527	4.79E-12
IFLc	interval first to last insemination cow	18	59319899	0.1638	-3.59126	3.53318	1.44E-08
NRc	non-return rate cow	18	59319899	0.1627	-3.57774	3.48832	1.61E-07
CEd	percentage normal births	18	59355012	0.2118	3.98515	5.30323	8.38E-12
CEd	percentage normal births	18	59502602	0.5733	2.54231	3.16229	8.19E-11
CEd	percentage normal births	18	59658540	0.8714	4.15934	3.87793	1.81E-15
BWd	birth weight	18	59658540	0.8331	-5.8348	9.46644	1.44E-08
CEm	percentage normal births	18	59658540	0.8724	4.66406	4.84336	2.07E-08
BWm	birth weight	18	59658540	0.8276	-6.11119	10.6571	6.20E-08
CEd	percentage normal births	18	59723348	0.0633	4.47874	2.37861	9.65E-11
CEd	percentage normal births	18	59772517	0.1322	-5.95853	8.14662	4.86E-27
BWd	birth weight	18	59772517	0.1733	7.2403	15.0222	4.58E-12
CEm	percentage normal births	18	59772517	0.1311	-5.60267	7.15266	9.99E-11
BWm	birth weight	18	59772517	0.1787	7.28515	15.5755	3.00E-10
IFLc	interval first to last insemination cow	18	59772517	0.1345	-3.7576	3.28749	1.47E-07

GLd	gestation length	18	59772517	0.1714	5.35066	8.13102	2.09E-07
GLm	gestation length	18	59772517	0.1734	5.54695	8.81936	1.78E-06
CEd	percentage normal births	18	60109062	0.5364	2.43188	2.94139	6.33E-09
BWd	birth weight	18	60109062	0.4857	-3.78034	7.13963	1.22E-06
CEd	percentage normal births	18	60111726	0.3313	2.43397	2.62507	9.88E-07
CEd	percentage normal births	18	60115619	0.4545	-2.43333	2.93603	7.43E-09
BWd	birth weight	18	60115619	0.5106	3.97172	7.88372	3.63E-07
CEd	percentage normal births	18	60122922	0.4653	-2.43198	2.94304	5.32E-09
BWd	birth weight	18	60122922	0.5173	3.67143	6.73165	2.51E-06
CEd	percentage normal births	18	60122998	0.5347	2.42458	2.9251	5.81E-09
BWd	birth weight	18	60122998	0.4827	-3.67143	6.73165	2.51E-06
CEd	percentage normal births	18	60123658	0.7034	2.8158	3.30857	1.13E-10
BWd	birth weight	18	60123658	0.617	-4.66815	10.2996	7.48E-09
BWm	birth weight	18	60123658	0.6102	-4.34913	8.99795	1.24E-06
CEd	percentage normal births	18	60124094	0.2966	-2.82874	3.33857	9.90E-11
BWd	birth weight	18	60124094	0.383	4.66815	10.2996	7.48E-09
BWm	birth weight	18	60124094	0.3898	4.34913	8.99795	1.24E-06
CEd	percentage normal births	18	60124165	0.2966	-2.81661	3.31025	1.18E-10
BWd	birth weight	18	60124165	0.3831	4.66721	10.2962	7.51E-09
BWm	birth weight	18	60124165	0.3899	4.33661	8.94693	1.32E-06
CEd	percentage normal births	18	60126494	0.7036	2.81557	3.30638	1.10E-10
BWd	birth weight	18	60126494	0.6173	-4.4466	9.34224	3.85E-08
CEd	percentage normal births	18	60130599	0.535	2.47337	3.04381	2.87E-09
BWd	birth weight	18	60130599	0.4828	-3.6705	6.72829	2.50E-06
CEd	percentage normal births	18	60132042	0.8364	4.21279	4.85683	1.44E-19
CEm	percentage normal births	18	60132042	0.8365	3.94127	4.24807	1.17E-07
BWd	birth weight	18	60132042	0.742	-4.07166	6.34773	3.40E-06
CEd	percentage normal births	18	62084711	0.5358	-2.34679	2.73961	1.02E-09
CEd	percentage normal births	18	62162015	0.5227	-1.7436	1.51695	3.34E-06
CEd	percentage normal births	18	63178062	0.0547	-4.42717	2.02704	8.07E-12
GLd	gestation length	18	63178062	0.1288	5.86189	7.71111	1.98E-07
BWd	birth weight	18	63178062	0.1279	5.31102	6.29117	3.94E-06
GLd	gestation length	18	63232298	0.8582	-5.67504	7.83773	7.47E-08
CEd	percentage normal births	18	63404726	0.7697	1.88441	1.25888	7.39E-07
GLd	gestation length	18	64017719	0.0987	6.70922	8.00619	1.21E-07
GLm	gestation length	18	64017719	0.0994	6.61768	7.84269	3.83E-06
CEd	percentage normal births	18	64875728	0.1838	2.03423	1.24132	2.73E-06
SBd	percentage live births	18	65572003	0.707	6.68407	18.5092	1.79E-06
GLm	gestation length	19	37519829	0.9703	9.95095	5.69901	4.15E-06
SBm	percentage live births	20	33809571	0.8816	7.83013	12.801	1.22E-06
SBm	percentage live births	20	33860963	0.226	5.23819	9.60006	2.00E-06
DFS	interval calving to insemination	20	34800041	0.154	-3.39651	3.00624	5.71E-07
IFLc	interval first to last insemination cow	20	34800041	0.1566	-3.08087	2.5067	1.41E-06
CEd	percentage normal births	21	8085052	0.4026	-1.86249	1.6686	1.31E-07

DFS	interval calving to insemination	21	46641405	0.2262	-2.85098	2.84538	7.36E-07
DFS	interval calving to insemination	21	46654373	0.3078	-2.46262	2.58402	1.18E-06
DFS	interval calving to insemination	21	53671014	0.9561	4.95284	2.05733	7.49E-08
DFS	interval calving to insemination	21	54729058	0.0594	-3.83215	1.63978	2.62E-06
DFS	interval calving to insemination	21	55195263	0.9167	3.46425	1.83267	4.87E-07
DFS	interval calving to insemination	21	55268653	0.973	8.72399	3.99235	1.08E-12
IFLc	interval first to last insemination cow	21	55268653	0.9716	6.09288	2.04771	9.67E-08
DFS	interval calving to insemination	21	55464173	0.0687	-3.66715	1.72174	1.32E-06
DFS	interval calving to insemination	21	56056064	0.9329	3.41779	1.46246	2.06E-06
DFS	interval calving to insemination	21	58564557	0.9728	5.79749	1.78179	8.76E-08
NRh	non-return rate heifer	23	45311468	0.9262	-4.63753	2.94111	2.37E-06
CEd	percentage normal births	29	48027873	0.7943	-1.89702	1.17592	3.37E-07
CEd	percentage normal births	29	48411786	0.8767	2.04869	0.907531	4.13E-06
NRc	non-return rate cow	29	48621040	0.3913	-2.02971	1.96254	1.26E-06
CEd	percentage normal births	29	49234278	0.625	1.59205	1.18812	8.14E-08
CEd	percentage normal births	29	49646800	0.7658	2.22174	1.77039	1.13E-10
SBd	percentage live births	29	49646800	0.763	7.26634	19.0962	2.44E-07
NRc	non-return rate cow	29	49667536	0.1743	-2.31383	1.54121	3.93E-06
CEd	percentage normal births	29	50618364	0.1016	-2.66216	1.29352	5.80E-08
CEd	percentage normal births	29	50642202	0.9018	2.37772	1.00132	5.89E-07
SBd	percentage live births	29	50642202	0.9166	9.80218	14.6898	2.65E-06

Supplementary Table S6: Comprehensive list of potential candidate causative variants and their segregation in different dataset and populations

name	haplotype information											
	**chrom**	**start[a]**	**end[a]**	**haplotype frequency**	**observed homozygous carrier**	**expected homozygous carrier**	**approach**	**chr**	**position**	**reference allele**	**variant allele**	**gene**
HH21[c]	7	7871925	10432630	0.055	0	157	trio & pgp	7	7913459	CTTG	C	*NOTCH3*
HH3[de]	8	90958661	92084831	0.014	0	10	trio & pgp	8	93753358	T	C	*SMC2*
HH25	10	86876435	87772797	0.020	5	20	trio	10	84938370	CTGGTGGAGGC GCAGACCCCGG CGGCACGCT	C	*RIOX1*
HH13[f]	18	60931980	62100899	0.018	1	17	trio & pgp	18	62758881	G	A	*KIR2DS1*
HH35	26	3358717	4234871	0.024	7	31	pgp	26	5325675	C	G	*PCDH15*

[a] according to the reference sequence ARS-UCD1.2 (National Center for Biotechnology Information, 2018a)
[b] according to the NCBI Annotation Release 106 (National Center for Biotechnology Information, 2018b)
[c] haplotype described before as 07-126 by VanRaden et al. (2011) and Sahana et al. (2013)
[d] haplotype previously described by VanRaden *et al.* (2011), McClure *et al.* (2013), Sahana *et al.* (2013) and Wu *et al.* (2019)
[e] variant previously detected by McClure *et al.* (2014)
[f] haplotype previously described by Fritz *et al.* (2013)

candidate variant					
**gene name**	**associated disorder / gene function**	**OMIM / OMIA**	**effect**	**transcirpt[b]**	**base change[b]**
notch receptor 3	myofibrimatosis / cerebral arteriopathy with subcortical infarcts and leukoencephalopathy, type 1 (CADASIL1)	600276 + 125310	disruptive inframe deletion	XM_003586246.3	c.129_131delTTG
structural maintenance of chromosomes 2	embryonic lethality	605576 / 001824-9913	missense variant	XM_015472668.2	c.3404T>C
ribosomal oxygenase 1	chromatin organisation, negative regulation of osteoblast differentiation, negative regulation of transcription, DNA-templated	611919	disruptive inframe deletion	NM_001099702.1	c.396_425delGGCGC AGACCCCGGCGGC ACGCTTGGTGGA
killer cell immunoglobulin like receptor, two Ig domains and short cytoplasmic tail 1	function in placentation and during pregnancy, integral component of membrane	604952	stop gain variant	NM_001097567.1	c.475C>T
protocadherin related 15	Usher syndrome, deafness	605514	missense variant	XM_015460562.2	c.2599C>G

					WGS information 691 genomes						SWISSCOW informa				
**amino acid change**	**siftScore**	**siftScore prediction (cutoff=-2.5)**	**average PhyloP**	**average phastCons**	**allele frequency**	**LD**	**ref hom**	**var het**	**var hom**	**missing**	**allele frequency LD**	**LD**	**ref hom**	**var het**	**var hom**
p.Cys44del	-6.75	deleterious	2.61	0.984	0.014	0.49	657	8	0	26					
p.Phe1135Ser	-7.67	deleterious	7.74	1	0.014	1.00	679	11	0	1	0.017	0.85	5447	147	0
p.Ala133_Glu142del	0.659	neutral	NA	NA	0.016	1.00	603	25	1	62					
p.Gln159*			-0.517	0.0176	NA	NA	666	22	0	3	0.018	0.49	5030	557	0
p.Leu867Val	-0.507	neutral	2.08	1	0.014	1.00	663	27	1	0					

ation Holstein population				SWISSCOW				1kBulls information (run 8; 4109 genomes)				
expected var hom	missing	allele frequeny	pHWE (Chi-square)	ref hom	var het	var hom	missing	ref hom	var het	var hom	missing	breed occurence
								3743	3	0	363	Holstein, Salers, Shorthorn
1	9	0.013	1	8628	1	0	9	4018	59	0	32	Holstein
								3480	97	16	516	Holstein, Angus, Belgian Blue, Bohuskulla, Brown Swiss, Charolais, Chianina, DSB, Fjäll, Hereford, Jersey, Limousin, Montbeliarde, Normande, Norwegian Red, Ringamako, Rölla, Romagnola, Shorthorn, Simmental, Tarentaise, Ukrainian Grey, Yakut, Yanbian
12	16	0.050	7E-07	8611	8	0	19	3963	132	0	14	Holstein, Angus, Ayshire, Charolais, Danish Red Dairy, DSB, Fjäll, Maine Anjou, Meuse Rhine Ysel, Norwegian Red, Original Braunvieh, Podolian, Ringamako, Romagnola, Shorthorn, Simmental, Swedish Red, Väko
								3992	91	7	19	Holstein, Ayrshire, Norwegian Red
